# Bioorthogonal Azide–Thioalkyne Cycloaddition Catalyzed by Photoactivatable Ruthenium(II) Complexes

**DOI:** 10.1002/anie.202103645

**Published:** 2021-06-16

**Authors:** Alejandro Gutiérrez‐González, Paolo Destito, José R. Couceiro, Cibran Pérez‐González, Fernando López, José L. Mascareñas

**Affiliations:** ^1^ Centro Singular de Investigación en Química Biolóxica e Materiais Moleculares (CiQUS) and Departamento de Química Orgánica Universidade de Santiago de Compostela 15782 Santiago de Compostela Spain; ^2^ Misión Biológica de Galicia Consejo Superior de Investigaciones Científicas (CSIC) 36080 Pontevedra Spain

**Keywords:** biocompatible reactions, bioorthogonal reactions, click chemistry, ruthenium, thioalkynes

## Abstract

Tailored ruthenium sandwich complexes bearing photoresponsive arene ligands can efficiently promote azide–thioalkyne cycloaddition (RuAtAC) when irradiated with UV light. The reactions can be performed in a bioorthogonal manner in aqueous mixtures containing biological components. The strategy can also be applied for the selective modification of biopolymers, such as DNA or peptides. Importantly, this ruthenium‐based technology and the standard copper‐catalyzed azide–alkyne cycloaddition (CuAAC) proved to be compatible and mutually orthogonal.

## Graphical abstract

Tailored cationic Ru^II^ complexes are highly effective precatalysts for ruthenium‐catalyzed azide–thioalkyne cycloaddition (see scheme). These reactions can now be carried out under very dilute conditions and in complex biorelevant media; they are also compatible with and mutually orthogonal to CuAAC. Moreover, ruthenium–arene sandwich complexes function as photoactivatable precatalysts, providing for temporal control of the reactivity.

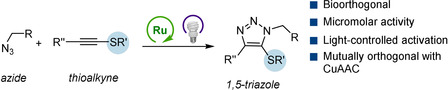

## Introduction

Bioorthogonal reactions, by enabling the covalent modification of specific reactants or biomolecular targets in complex biological environments, have brought a paradigm shift on the potential of chemistry for interrogating or/and altering biology.[^1^, ^2^] Within the “toolbox” of bioorthogonal reactions, those that are catalyzed by transition metals are especially attractive, owing to their intrinsic metal‐dependent characteristics, and the possibility of tuning the reactivity by adjusting the characteristics of the catalyst.^[3]^ However, progress in this field has been slow, in great part because of the notion that transition metal reagents are incompatible with aqueous and biological milieu, and that they can be easily inactivated by biological components. Moreover, while there has been an increasing number of reports on bioorthogonal metal‐catalyzed reactions, they usually present low catalytic efficiencies, especially under the diluted conditions usually required for biological applications.[^3^, ^4^]

**Figure 1:**
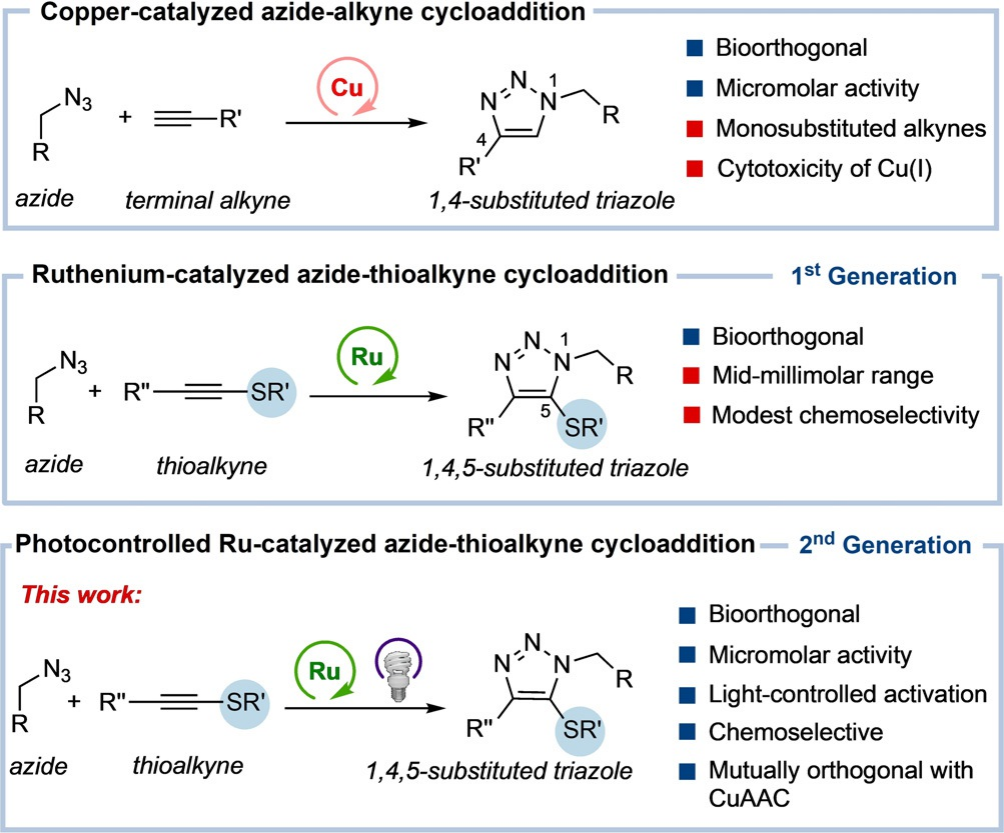
Metal‐catalyzed azide–(thio)alkyne cycloaddition reactions. Advantages are marked with blue squares, and limitations with red.

Among all transition‐metal‐mediated bioorthogonal reactions, there is one that stands out, namely, the copper(I)‐promoted azide–alkyne cycloaddition (CuAAC).[^5^, ^6^] The reaction engages organic azides and alkynes, which are ideal chemical entities in terms of biological orthogonality, and tends to exhibit very good rates. However, this transformation still presents important limitations such as its low compatibility with thiols, its restriction to terminal alkynes or the side reactivity and toxicity of copper (I) ions in biological contexts.^[7]^ Furthermore, to reach efficient conversions under typically diluted conditions, the reactive copper(I) species need to be generated in situ using excess amounts of a copper(II) source and sodium ascorbate, a reductant which is not innocent in biological contexts (Figure 1).^[8]^ Therefore, there is a clear need to discover new, robust and aqueous‐compatible metal‐catalyzed annulations as alternatives to the CuAAC.[^9^, ^10^]

In this context, in 2017, we reported the first examples of a ruthenium‐catalyzed azide–alkyne cycloaddition that takes place in aqueous and in biologically relevant milieu.^[11]^ The method makes key use of thioalkynes as reaction partners, and of the commercially available complex [Cp*RuCl(COD)] (**Ru1**) as catalytic reagent (Figure 1). Following our report, other groups developed alternative conditions to achieve related cycloadditions in water, using nickel,^[12]^ rhodium^[13]^ or iridium catalysts,^[14]^ albeit only the latter has been shown to operate in biological mixtures.

Importantly, all these new metal‐catalyzed azide–alkyne cycloaddition reactions, including our ruthenium‐catalyzed process, have demonstrated effectivity only when the concentration of reagents is in the mid to high millimolar range. This represents an important limitation in terms of developing biological applications, which usually require very diluted samples. An additional challenge in this field has to do with the possibility of controlling the reactivity using external stimuli, as this could open new opportunities for biological regulation.

Herein, we demonstrate that cationic Ru^II^ complexes such as [Cp*Ru(MeCN)_3_]PF_6_ (**Ru2**) are excellent precatalysts to perform efficient, formal cycloadditions between thioalkynes and azides in aqueous media. Contrary to [Cp*RuCl(COD)] (**Ru1**), this cationic complex (**Ru2**) is very effective under diluted micromolar conditions, even in PBS and biologically complex media, such as DMEM, or HeLa cells lysates. More importantly, we also show that the azide–thioalkyne cycloaddition can be catalyzed by [Cp*Ru^II^arene] sandwich complexes,^[15]^ provided that they are photoactivated by a short‐time irradiation with a LED lamp at 365 nm (Figure 1). The possibility of controlling the generation of the catalytically active Ru^II^ species with light opens interesting perspectives in optobiology.^[16]^ Finally, we also demonstrate that this technology is fully orthogonal with the CuAAC and, moreover, it can be used for the chemoselective modifications of small peptides and ssDNAs, for instance, for the introduction of fluorogenic tags.

## Results and Discussion

**Figure 2:**
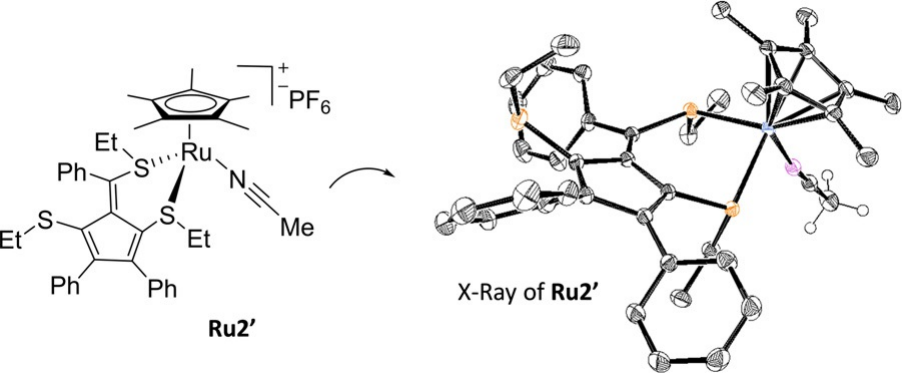
Structure and X‐ray crystallographic analysis of **Ru2′** (specific hydrogen atoms and the counterion (PF_6_^−^) are omitted for clarity).

Our first experiments were carried out with the anthracenyl azide **1 a** and the thioalkyne **2 a**, because the resulting triazole product (**3 aa**) is fluorescent and, thus, the reaction can be readily monitored. In consonance with previous observations, the neutral Ru^II^ complex [Cp*RuCl(COD)] (**Ru1**) was significantly more efficient than [Cp*Ru(MeCN)_3_]PF_6_ (**Ru2**), when the reaction was carried out under anhydrous conditions in CH_2_Cl_2_ (75 mM).^[17]^ Specifically, the reaction gave a 99 % yield of the product after 0.5 h with **Ru1** (Table 1, entry 1), but just a 15 % yield with **Ru2**, after 1 h (30 % yield after 6 h, entry 2). Several control experiments and careful analysis by NMR and ESI‐MS allowed to discover that the poorer performance of **Ru2** was likely due to the formation of secondary ruthenium‐containing products. In particular, we could identify **Ru2′**, which results from an unprecedented ruthenium‐promoted trimerization of thioalkynes, a process that generates a chelating dithiofulvene ligand (Figure 2).^[18]^ The performance of **Ru2** in the RuAtAC could be partially improved using Et_3_NCl as additive (5 mol %), probably by favoring the in situ formation of a neutral Cp*–ruthenium(II) chloride species (entry 3), which might hamper the thioalkyne‐to‐fulvene trimerization.^[19]^

**Table 1:** Viability of the RuAtAC with the Ru^II^ cationic complex **Ru2**.^[a]^ 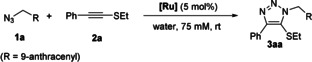

Entry	**[Ru]** (%)	Solvent	*t* [h]	Yield [%]^[b]^
1	**Ru1** (5)	CH_2_Cl_2_	0.5	99
2	**Ru2** (5)	CH_2_Cl_2_	1	15 (30)^[c,d]^
3^[e]^	**Ru2** (5)	CH_2_Cl_2_	1	28 (72)^[c]^
4^[e]^	**Ru2** (5)	H_2_O	0.5	99
5	**Ru2** (5)	H_2_O	0.5	4

[a] Reaction conditions: **2 a** (150 μmol), **1 a** (75 μmol), solvent (1 mL) and the ruthenium catalyst (5 mol %) were added to a vial under air, and the mixture stirred for the indicated time. [b] Yield determined by ^1^H NMR spectroscopy of the reaction crude mixture using 1,3,5‐trimethoxybenzene as an internal standard. [c] The yield after 6 h is indicated in parenthesis. [d] The complex **Ru2′** was detected in the reaction mixture (NMR and ESI‐MS).^[18]^ [e] The Ru complex **Ru2** and Et_4_NCl (5 mol %) were premixed in the corresponding solvent for 5 min. Note: Reaction mixtures in water can be considered as suspensions rather than solutions.

Remarkably, when the reaction catalyzed by **Ru2** was carried out in water, in the presence of this chloride source, the cycloadduct **3 aa** was obtained in 99 % yield, after only 0.5 h (entry 4). We later found that the use of Et_3_NCl is not needed, as the reaction provided the same yield without any additive (entry 5). Most likely, the higher activity of **Ru2** in water is partially related to the formation of active Ru^II^ aquo or oxo derivatives, which favor the desired annulation over alternative pathways.^[20]^ Indeed, we have detected by ESI‐MS several ruthenium‐oxygenated species in the aqueous solutions of **Ru2**.^[21]^

**Scheme 1:**
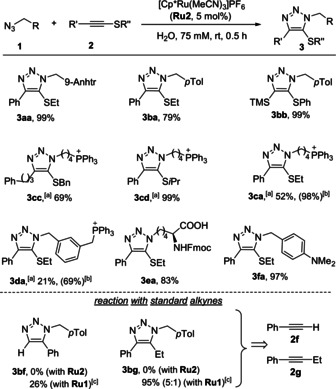
Scope of the annulation using the complex **Ru2**. [a] The counterion is probably bromide, as in the starting material. [b] Yield of the reaction carried out using a H_2_O/DMSO (9:1) mixture is shown in parenthesis. [c] Carried out using **Ru1** instead of **Ru2**. Fmoc=fluorenylmethoxycarbonyl.

Importantly, using these conditions, we could promote the annulation of a variety of azides and thioalkynes (Scheme 1). Thioalkynes bearing an ethyl group at the sulfur atom were particularly reactive, but other alkyl groups like benzyl (**2 c**), isopropyl (**2 d**) or aromatic substituents (**2 b**) are also tolerated, providing in all cases the expected products in good yields. The other substituent of the thioalkyne can also be modified without compromising the yields of the desired triazoles.

**Ru2** proved to be much more selective than the previously described catalyst **Ru1** with respect to the type of alkyne partner used. Indeed, **Ru1** promotes the reaction of regular alkynes lacking the thioether, such as the **2 f** and **2 g**, to give the corresponding triazoles in moderate to good yields (**3 bf**, **3 bg**, Scheme 1). However, the cationic reagent **Ru2** failed to induce any conversion with these alkynes, even after 7 h at rt (Scheme 1). Therefore, **Ru2** not only allows to carry out the annulation in aqueous media in an efficient manner, but also introduces a level of chemoselectivity that was previously unattainable with **Ru1**, allowing to fully distinguish thioalkynes from alkynes (both mono‐ and disubstituted).

Considering this chemoselectivity, we next explored the orthogonality of the RuAtAC and the CuAAC. When a mixture of azide **1 b** (1 equiv.) and alkynes **2 a** and **2 h** (2 equiv. of each one) was treated under standard CuAAC conditions [that is, CuSO_4_⋅5 H_2_O (5 mol %) and sodium ascorbate (NaAsc, 10 mol %)] the triazole **3 bh**, resulting from the reaction of the terminal alkyne **1 h** was exclusively formed in high yield (Table 2, entry 1). If, after this reaction has been completed (2 h), we add a second equivalent of the azide, and the catalyst **Ru2** (5 mol %), the thioether containing adduct **3 ba** is formed in a good 78 % yield (entry 2). Even more relevant, when the initial mixture of the azide **1 b** and alkynes **2 a** and **2 h** is first treated with **Ru2**, only the sulfur‐containing triazole **3 ba** is observed (79 % yield, entry 3), whereas the subsequent addition of **1 a** and the Cu catalyst leads to **3 bh** in 95 % yield (entry 4). Furthermore, when azide **1 b** was mixed with both **2 a** and **2 h** (1 equiv. each) in the presence of both Cu and Ru catalysts (5 mol % of each one), an almost equimolar mixture of **3 ba** and **3 bh** is obtained, in an excellent yield (94 % yield, entry 5). Overall, these results confirm a striking chemoselectivity and mutual orthogonality between both methods and indicate that both cycloadditions share similar kinetic profiles under these conditions. This mutual orthogonality promises relevant applications, such as for the dual tagging of biomolecules.^[22]^

**Table 2:** Orthogonality between RuAtAC and CuAAC annulations.^[a]^ 
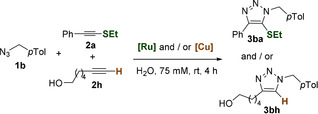

Entry	Catalyst (reaction time)	Yield [%]^[b]^	
		**3 ba**	**3 bh**
1	**[Cu]** (2 h)	0	78
2^[c]^	**[Cu]** (2 h); then **[Ru]** (2 h)^[c]^	79	78
3	**[Ru]** (2 h)	79	0
4^[c]^	**[Ru]** (2 h); then **[Cu]** (2 h)^[c]^	78	95
5^[d]^	**[Ru]** and **[Cu]** (2 h)	44	50

[a] Reaction conditions: A solution of **2 a** (2.0 equiv), **2 h** (2.0 equiv), **1 b** (1.0 equiv; 0.75 mmolg^−1^) in DMSO was added to water (500 μL, 75 mM), followed by the corresponding catalyst: either **[Ru]** (corresponds to **Ru2,** 5 mol %) or **[Cu]** (corresponds to CuSO_4_⋅5 H_2_O, 5 mol %, NaAsc, 10 mol %), in a vial open to air. [b] Yield determined by ^1^H NMR spectroscopy using 1,3,5‐trimethoxybenzene as an internal standard. [c] After 2 h, a second equivalent of **1 b** was added, followed by the second catalyst. [d] Both **[Ru]** and **[Cu]** were present from the beginning.

In view of the excellent performance of **Ru2**, we explored its behavior under more diluted conditions. Gratifyingly, the annulation between **1 c** and **2 a** can be efficiently carried using azide concentrations of 1 mM (99 % yield), and even at 250 μM, without significant deterioration in the yield (86 % yield).^[23]^

At this point we questioned the possibility of engineering ruthenium derivatives that could be activated using external stimuli, owing to the ensuing possibilities for introducing temporal control on the activity. Considering that the precatalyst **Ru2** can be made from Ru^II^ arene sandwich complexes of type [Cp*Ru(arene)]X by UV irradiation in acetonitrile,[^15^, ^24^] we anticipated that the catalytic species resulting from mixing **Ru2** with water could be equally generated from these Ru^II^ sandwich complexes, provided that the arene ligand could be easily released with light under aqueous conditions. This idea was attractive not only as a means to control reactivity, but also because of the high stability of the ruthenium(II) sandwich precursors, which might be especially useful to avoid its deactivation in biological contexts.

We therefore synthetized the naphthalene derivative [Cp*Ru(naphthalene)]BPh_4_ (**Ru3**), and two analogs with pyrene ligands: [Cp*Ru(pyrene)]PF_6_ (**Ru4**) and [Cp*Ru(pyrene‐SO_3_Na)]PF_6_ (**Ru5**).^[24]^ Their potential as photoactivatable catalysts for the RuAtAC was tested in a model reaction using thioalkyne **2 a** and the *p*‐tolyl azide **1 b** (Table 3).^[25]^ Not surprisingly, treatment of **1 b** and **2 a** with 5 mol % of **Ru3**, in a 9:1 water:CH_3_CN mixture, did only provide traces of the triazole product (<3 % yield, Table 3, entry 1).^[23]^ However, we were glad to observe that when this mixture was irradiated with a 365 nm LED lamp for 10 min, the cycloaddition proceeded smoothly to afford the desired triazole **3 ba** in 90 % yield (entry 2). The related pyrene complex **Ru4** was slightly more efficient, providing a 99 % yield upon photoactivation (10 min/ 365 nm, entry 4). In absence of light there is no conversion (entry 3). The sulfonate derivative **Ru5** also works under irradiation, although it is less efficient.

**Table 3:** Viability of the cycloaddition with phototoactivated Cp*Ru arene complexes **Ru3**, **Ru4** and **Ru5**.^[a]^ 
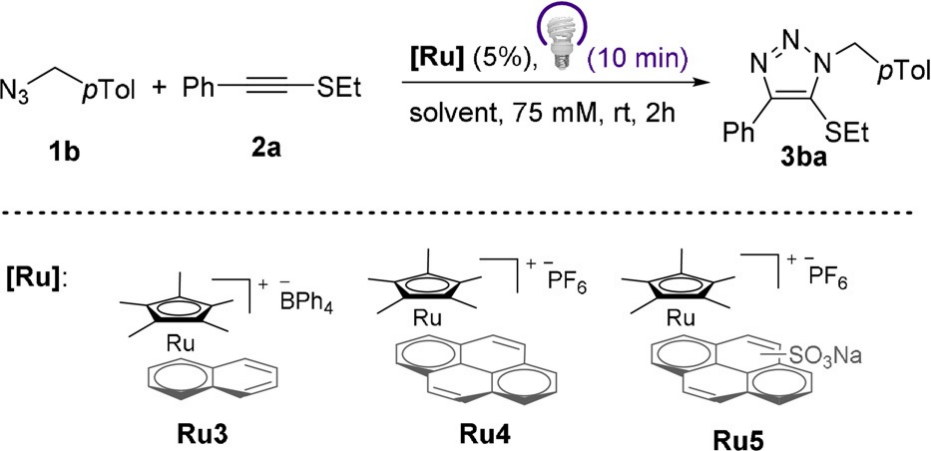

Entry	**[Ru]**	Solvent	Conv. [%]^[b]^	Yield [%]^[b]^
1	**Ru3**	H_2_O/MeCN (9:1)	3	3
2	**Ru3**+*hν*	H_2_O/MeCN (9:1)	90	90
3	**Ru4** ^[c]^	H_2_O/MeCN (9:1)	1	0
4	**Ru4**+*hν*	H_2_O/MeCN (9:1)	99	99
5	**Ru5**+*hν*	H_2_O/MeCN (9:1)	80	70

[a] Conditions for the reaction under irradiation: **2 a** (2.0 equiv), **1 b** (1.0 equiv), the solvent and **[Ru]** were sequentially added to a vial, which was closed and irradiated with a 365 nm LED lamp for 10 min, and the mixture stirred for 2 h. [b] Yield and conversion determined by ^1^H NMR spectroscopy using 1,3,5‐trimethoxybenzene as an internal standard. [c] The reaction was carried out without irradiation.

**Figure 3:**
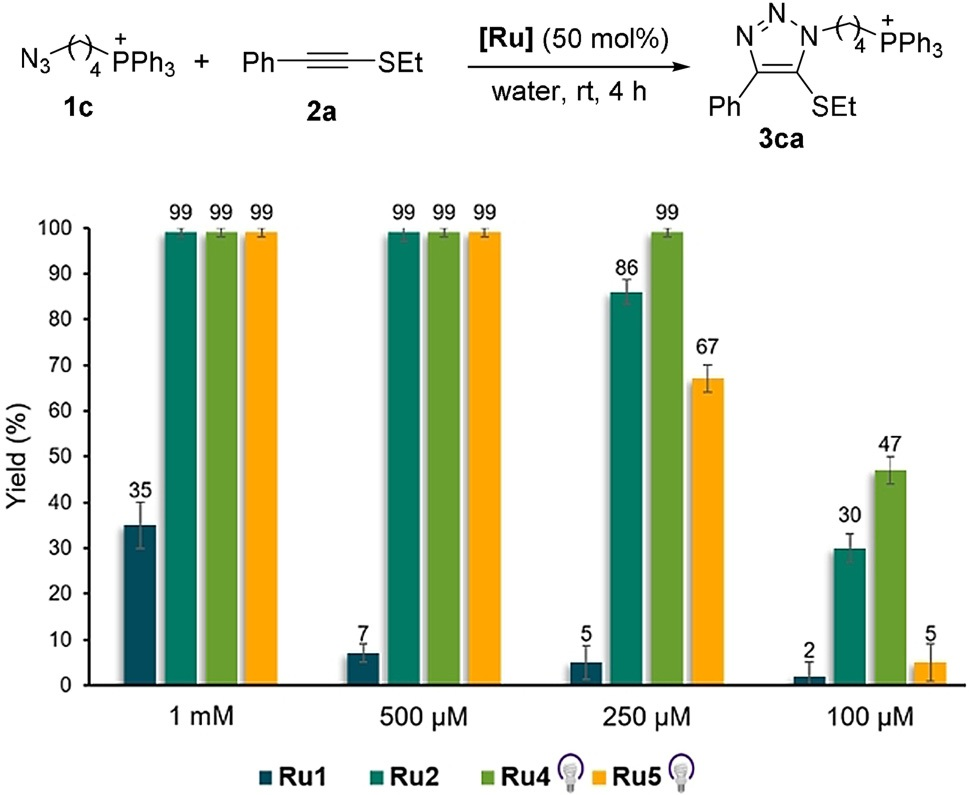
Catalyst performance in the micromolar range. Reactions were conducted in HPLC vials, and yields were determined by UHPLC–MS using coumarin as an internal standard. Results are the average of three different reactions. Reaction mixtures in the presence of **Ru4** and **Ru5** were irradiated for 15 min at 365 nm to activate the catalyst. Controls without irradiation for **Ru4** and **Ru5** provided yields <1 %. Note: The counterion in **1 c** is Br^−^.

With these results in hand, we next studied the behavior of precatalysts **Ru2, Ru4** and **Ru5** under more diluted conditions. To carry out these assays we used as substrate the water soluble triphenylphosphonium‐containing azide **1 c**, because the phosphonium tag facilitates a highly precise monitoring by LC–ESI‐MS. For comparative purposes, we also analyzed the performance of our first generation catalyst [Cp*RuCl(COD)] (**Ru1**), which had been poorly effective at the micromolar range. Reactions were carried out in water, at rt for 4 h, using different concentrations of the azide **1 c** (1 mM, 500 μM, 250 μM and 100 μM) and 50 mol % of the ruthenium complexes (Figure 3 and Figure S5). In the case of the photoactivatable complexes **Ru4** and **Ru5**, the reaction mixture in water, without any cosolvent,^[26]^ was irradiated for 15 min with a 365 nm LED. Gratifyingly, as can be seen in the Figure 3, the three ruthenium complexes provided quantitative yields of the product when using azide concentrations of 1 mM and 500 μM (after 4 h), whereas the chloride ruthenium complex **Ru1** provided only a modest 35 % yield with an azide concentration of 1 mM, or very poor yields at higher dilutions (2–7 % yield). The results at 250 μM showed that the pyrene Ru^II^ complex **Ru4** is the most active, producing a quantitative yield of **3 ca**, while the analogue complex bearing a sulfonate moiety (**Ru5**) led to a moderate 67 % yield. The tris(acetonitrile)ruthenium(II) catalyst (**Ru2**) and the photoactivatable pyrene complex **Ru4** were the most effective catalysts when the concentration of the azide was decreased down to 100 μM (30 and 47 % yield, respectively). The amount of **Ru2** and **Ru4** can be decreased up 15 mol % without significantly eroding the efficiency (52 and 84 % yield at 250 μM, respectively with **Ru2** and **Ru4**; see Figures S6 and S7).^[27]^

**Scheme 2:**
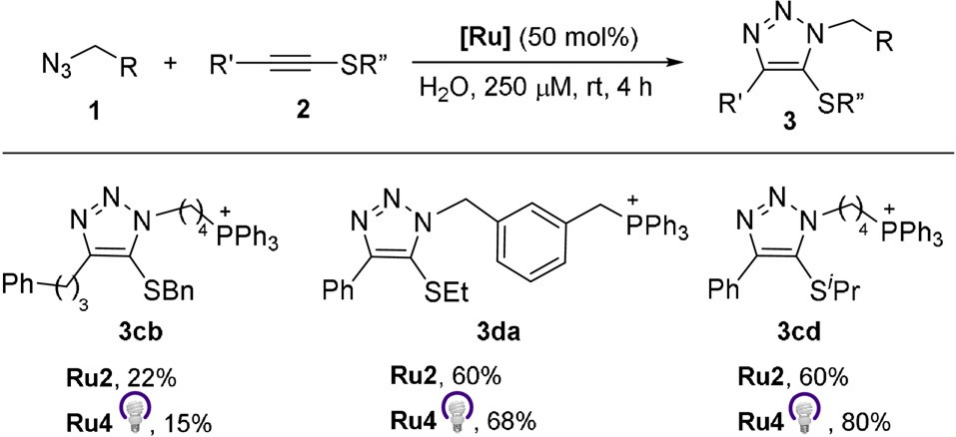
Scope of the reaction at micromolar concentrations (**1**: 250 μM; **2**: 500 μM). Reactions were conducted in HPLC vials open to air and yields were determined by UHPLC–MS, using coumarin as an internal standard. Results are the average of three different reactions. Reactions using **Ru4** were irradiated for 15 min at 365 nm.

As indicated in Scheme 2, the reaction under these diluted conditions is not limited to **1 c** and **2 a**; other azides and thioalkynes are also suitable reactants, confirming the potential of the methodology, either using the precatalyst **Ru2** or the light‐activatable precursor **Ru4** (Scheme 2).

**Figure 4:**
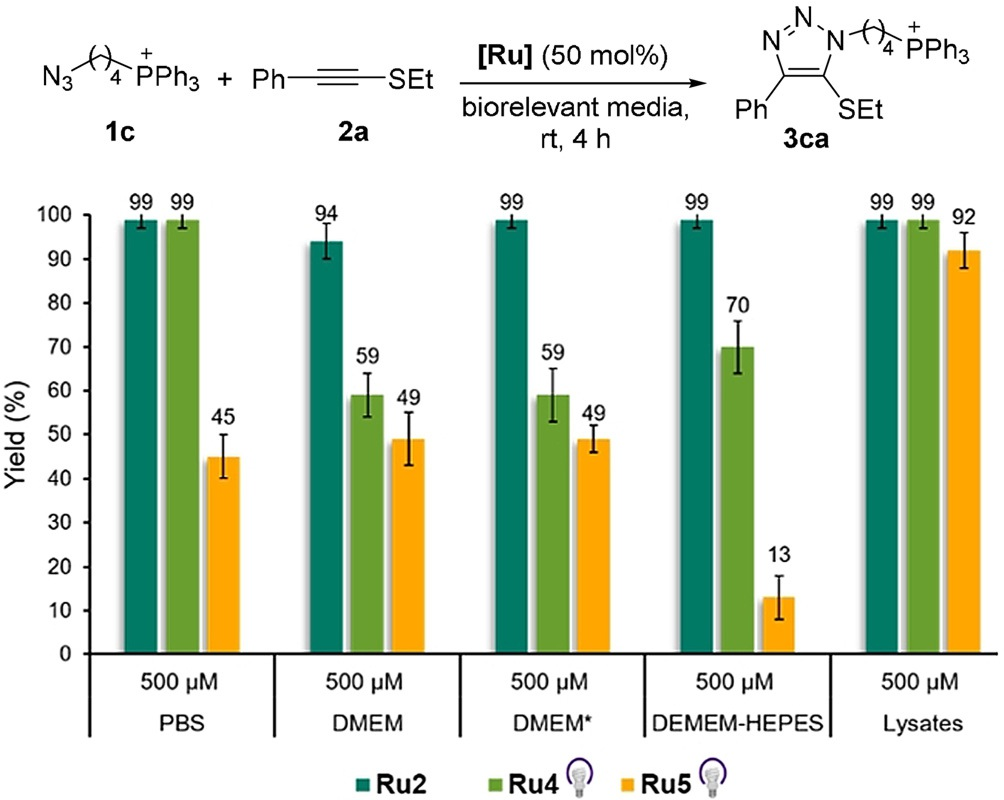
Reactivity of **Ru2**, **Ru4** and **Ru5** under biologically relevant conditions at 500 μM (**1 c**). Reactions were conducted in HPLC vials. Reaction yields were measured by UHPLC–MS using an internal standard (coumarin). Reactions with **Ru4** and **Ru5** were carried out by irradiating the mixtures for 15 min at 365 nm to activate the catalyst. PBS=phosphate buffer solution; DMEM=Dulbecco's Modified Eagle Medium; DMEM*=DMEM+10 % fetal bovine serum+1 % antibiotics; DEMEM‐HEPES=DMEM without phenol red and with HEPES (4‐(2‐hydroxyethyl)‐1‐piperazineethanesulfonic acid); Lysates=HeLa cell lysate 5 mg mL^−1^.

At this stage, we moved to more demanding, biologically relevant environments, using a 500 μM concentration of the azide and 50 mol % of the ruthenium complexes (Figure 4). Gratifyingly, the cationic complex **Ru2** provided very good results regardless of the biological media used. Thus, yields above 94 % were obtained in PBS, cell culture milieu (DMEM), both with and without Fetal Bovine Serum, and even in presence of HeLa cell lysates. The photoactivatable complex **Ru4** also showed an excellent performance under light irradiation, providing quantitative yields in PBS as well as in Hela cell lysates, and good yields, from 59 to 70 %, in cell culture media. Curiously, **Ru5** (with light) performed worse than **Ru4** and **Ru2**.

**Figure 5:**
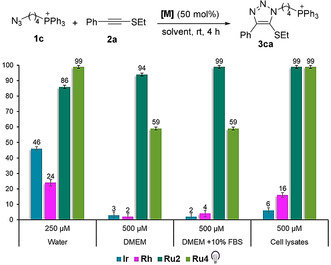
Comparison of different metal catalysts in the model reaction. In all examples, 50 mol % of the metal complex was used. **Ir** stands for [Ir(COD)Cl]_2_; **Rh** stands for [Rh(CO_2_)Cl]_2_. Reactions were performed using the optimal azide/thioalkyne ratios found for each metal catalyst (1.5:1 for **Ir** and 1:2 for **Rh** and **Ru** complexes). Yields were determined by UHPLC–MS using coumarin as an internal standard.

To better assess the potential of these catalytic systems, we compared their performance with that of Rhodium and Iridium complexes previously developed for related azide–alkyne annulations.[^13^, ^14^] Therefore, we tested the reaction of **2 a** and **1 c** in water (at 250 μM), as well as in the presence of cell culture media (at 500 μM), in both cases using 50 mol % of the metal complexes. Reactions with Rh and Ir complexes were carried out using the azide:thioalkyne ratio that had been identified as optimal for each of these metal catalysts (azide:thioalkyne=1.5:1 for Ir and 1:2 for Rh).^[23]^ As can be deduced from Figure 5, [Ir(COD)Cl]_2_ afforded the desired product in water in 46 % yield, whereas [Rh(CO_2_)Cl]_2_ only provided a 24 % yield of **3 ca**. Unfortunately, the performances of these metals dropped dramatically when used in biologically complex media such as DMEM or HeLa Cell lysates (Figure 5 and Figure S10). Therefore, our ruthenium complexes are clearly superior in water and, especially under biologically demanding conditions.

**Scheme 3:**
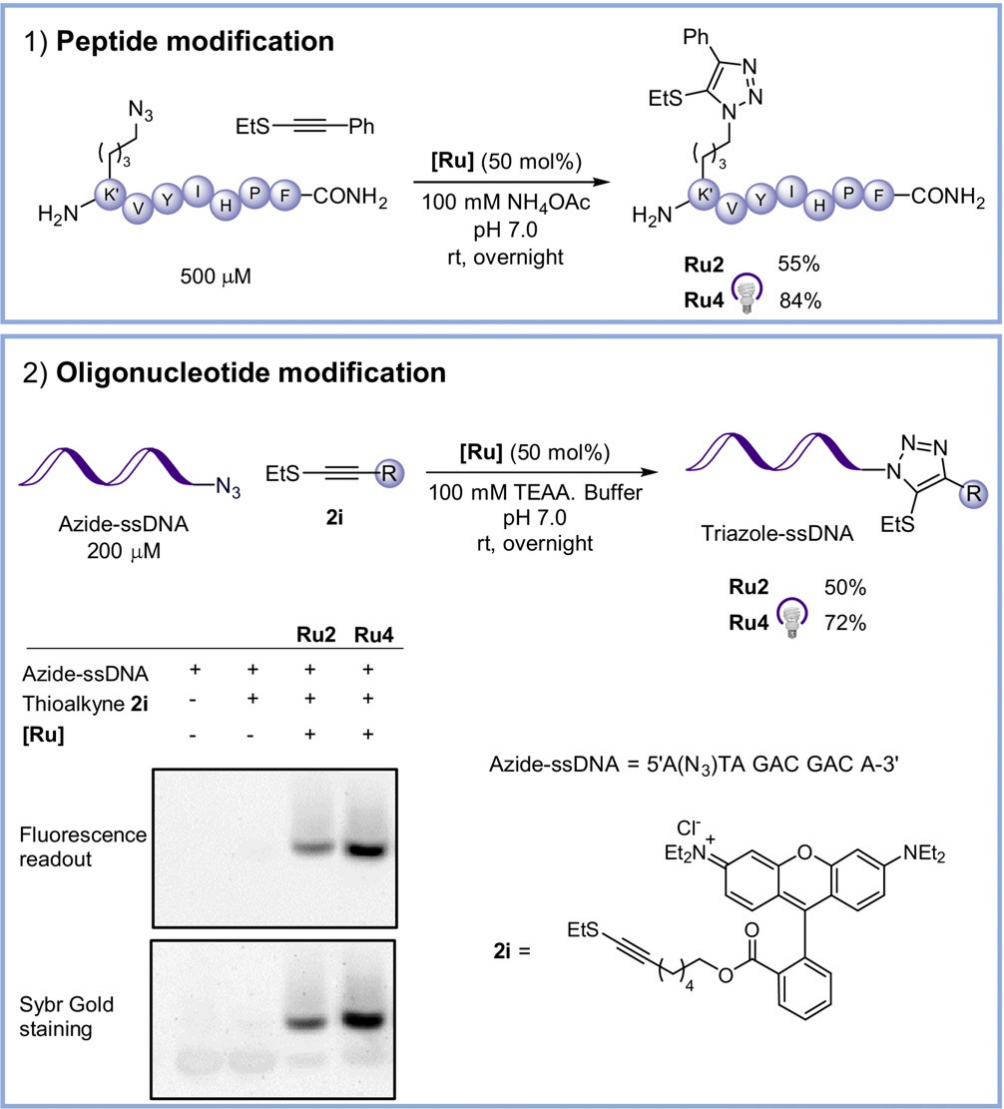
DNA and peptide labeling using **Ru2** and **Ru4** catalysts.

A particularly appealing application of bioorthogonal chemistry is related with the chemoselective modification of biopolymers. Therefore, we analyzed whether our catalysts could also be used for bioconjugation reactions with peptides or nucleic acids. As indicated in Scheme 3, the trisacetonitrile Ru^II^ complex **Ru2** is effective in promoting the cycloaddition between the thioalkyne **2 a** and an heptapeptide bearing a 6‐azidolysine at the N‐terminal position, to yield the corresponding peptide–triazole in 55 % yield [Scheme 3, Eq. (1)]. More importantly, when this reaction was carried out with the photoactivatable catalyst **Ru4**, under 15 min irradiation at 365 nm, the reaction proceeded even more efficiently, providing the desired peptide conjugate in an excellent 84 % yield. In the case of a ssDNA, we found that oligonucleotides containing an azide‐modified adenine at its 5′ end [Scheme 3, Eq. (2)], can be readily labelled with a thioalkyne derivative equipped with a Rhodamine tag, using either **Ru2** and **Ru4** (with irradiation), to give the expected products in 50 and 72 % yields, respectively. Therefore, again, the photoactivatable catalyst **Ru4** is the one presenting the best performance.

**Figure 6:**
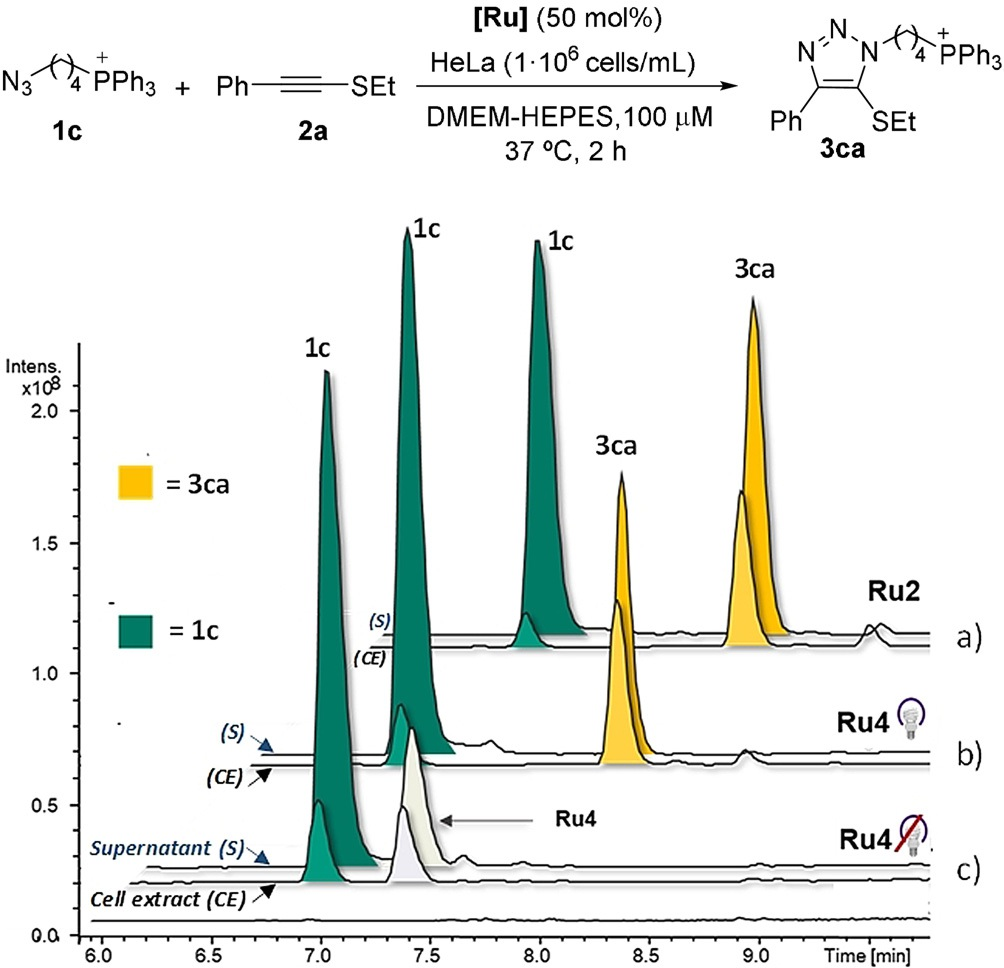
Reaction in the presence of HeLa cells suspended in DMEM‐HEPES. HPLC traces: MS signal of the methanolic cell extract (CE) and supernatant (S) for a) **Ru2**, b) **Ru4** (with 15 min irradiation), and c) **Ru4** without irradiation. See the Supporting Information for further details and control experiments.

As a final test of the potential of our new catalytic systems, we checked the viability of the annulations in cellular environments, using the phosphonium azide **1c**. The reaction could be monitored by LC–MS, owing to the sensitivity enhancement offered by phosphonium cations in mass spectrometry (Figure 6).^[23]^ The experiments were carried out by mixing the ruthenium catalyst (**Ru2** or **Ru4**), the thioalkyne (**2 a**, 800 μM) and azide **1 c** (100 μM) in DMEM–HEPES containing HeLa cells (1×10^6^ cells mL^−1^). After 2 h, the cells were centrifuged, the supernatant collected, and the cell pellets treated with MeOH (80 % aq.) to extract the cellular content. Both the extracellular media and the methanol extracts were analyzed by LC–MS. Either using **Ru2**, or the photoactivatable complex **Ru4** (with irradiation for 15 min), we were glad to detect the expected reaction product **3 ca**, both in the supernatant as well as in the methanolic extract.^[28]^ Worth to note, the methanolic extract turned out to be particularly rich in triazole product **3 ca**, whereas the supernatant contained considerable amounts of both azide (**1 c**) and triazole (**3 ca**). However, it is likely that at least part of the product internalizes after being formed. Overall, both, the photoactivatable complex **Ru4** and the cationic **Ru2**, were capable to promote the annulation under diluted conditions in cellular suspensions.

## Conclusion

We have discovered that specifically tailored cationic ruthenium (II) complexes are highly effective precatalysts to perform Ruthenium Catalyzed Azide Thioalkyne Cycloadditions (RuAtAC) in aqueous media. These precatalysts allow to carry out the annulation under dilute conditions, and also in complex, biorelevant media.

Moreover, the reaction is fully compatible and mutually orthogonal to the standard CuAAC. Importantly, the ruthenium reagents can be engineered as arene sandwich complexes to work as light‐activatable precatalysts. This provides not only for the temporal control of the reactivity, but also for higher efficiencies, likely because of the intrinsic stability of the metal complexes until receiving the optical stimulus. We have also demonstrated that the ruthenium complexes can be used for the orthogonal modification of azide‐tagged peptides and oligonucleotides, and that the reaction can be carried out in the presence of cells.

## Conflict of interest

The authors declare no conflict of interest.

## Supporting information

As a service to our authors and readers, this journal provides supporting information supplied by the authors. Such materials are peer reviewed and may be re‐organized for online delivery, but are not copy‐edited or typeset. Technical support issues arising from supporting information (other than missing files) should be addressed to the authors.

Supplementary

## References

[1] For reviews on bioorthogonal transformations, see:

[1a] E. M. Sletten , C. R. Bertozzi , Angew. Chem. Int. Ed. 2009, 48, 6974–6998; 10.1002/anie.200900942 PMC2864149 19714693

[1b] H. W. Shih , D. N. Kamber , J. A. Prescher , Curr. Opin. Chem. Biol. 2014, 21, 103–111; 25086220 10.1016/j.cbpa.2014.07.002

[1c] E. Brachet , P. Belmont , Curr. Org. Chem. 2016, 20, 2136–2160;

[1d] X. Ji , Z. Pan , B. Yu , L. K. De la Cruz , Y. Zheng , B. Ke , B. Wang , Chem. Soc. Rev. 2019, 48, 1077–1094; 30724944 10.1039/c8cs00395e

[1e] H. Wu , N. K. Devaraj in Cycloadditions in Bioorthogonal Chemistry (Eds.: M. Vrabel , T. Carell ), Springer International Publishing, Cham, 2016, pp. 109–130;

[1f] D. M. Patterson , J. A. Prescher , Curr. Opin. Chem. Biol. 2015, 28, 141–149; 26276062 10.1016/j.cbpa.2015.07.006

[1g] J. M. Baskin , C. R. Bertozzi , Aldrichimica Acta 2010, 43, 15–23;

[1h] D. M. Patterson , L. A. Nazarova , J. A. Prescher , ACS Chem. Biol. 2014, 9, 592–605; 24437719 10.1021/cb400828a

[1i] R. D. Row , J. A. Prescher , Acc. Chem. Res. 2018, 51, 1073–1081; 29727171 10.1021/acs.accounts.7b00606 PMC6190717

[1j] N. K. Devaraj , ACS Cent. Sci. 2018, 4, 952–959; 30159392 10.1021/acscentsci.8b00251 PMC6107859

[1k] M. Tomás-Gamasa , J. L. Mascareñas , ChemBioChem 2020, 21, 294–309. 31187598 10.1002/cbic.201900229

[2] For selected applications, see:

[2a] M. Grammel , H. C. Hang , Nat. Chem. Biol. 2013, 9, 475–484; 23868317 10.1038/nchembio.1296 PMC3866016

[2b] P. Agarwal , C. R. Bertozzi , Bioconjugate Chem. 2015, 26, 176–192. 10.1021/bc5004982 PMC4335810 25494884

[3] For reviews covering recent advances, see:

[3a] J. J. Soldevila-Barreda , N. Metzler-Nolte , Chem. Rev. 2019, 119, 829–869; 30618246 10.1021/acs.chemrev.8b00493

[3b] K. Vong , K. Tanaka in Handbook of In Vivo Chemistry in Mice: From Lab to Living System, Wiley-VCH, Weinheim, 2020, pp. 309–353;

[3c] P. Destito , C. Vidal , F. López , J. L. Mascareñas , Chem. Eur. J. 2021, 27, 4789–4816; 32991764 10.1002/chem.202003927

[3d] P. K. Sasmal , C. N. Streu , E. Meggers , Chem. Commun. 2013, 49, 1581–1587; 10.1039/c2cc37832a 23250079

[3e] M. Yang , Y. Yang , P. R. Chen , Top. Curr. Chem. 2016, 374, 2; 10.1007/s41061-016-0017-3 27573140

[3f] E. V. Vinogradova , Pure Appl. Chem. 2017, 89, 1619–1640;

[3g] M. Martínez-Calvo , J. L. Mascareñas , Coord. Chem. Rev. 2018, 359, 57–79;

[3h] A. H. Ngo , S. Bose , L. H. Do , Chem. Eur. J. 2018, 24, 10584–10594; 29572980 10.1002/chem.201800504

[3i] M. Martínez-Calvo , J. L. Mascareñas , Chimia 2018, 72, 791–801. 30514422 10.2533/chimia.2018.791

[4] For selected recent examples, see:

[4a] D. Chang , E. Lindberg , S. Feng , S. Angerani , H. Riezman , N. Winssinger , Angew. Chem. Int. Ed. 2019, 58, 16033–16037; 10.1002/anie.201907734 31478317

[4b] P. Destito , A. Sousa-Castillo , J. R. Couceiro , F. López , M. A. Correa-Duarte , J. L. Mascareñas , Chem. Sci. 2019, 10, 2598–2603; 30996975 10.1039/c8sc04390f PMC6419927

[4c] C. Vidal , M. Tomás-Gamasa , A. Gutiérrez-González , J. L. Mascareñas , J. Am. Chem. Soc. 2019, 141, 5125–5129; 30892889 10.1021/jacs.9b00837 PMC6497367

[4d] X. Wang , Y. Liu , X. Fan , J. Wang , W. S. C. Ngai , H. Zhang , J. Li , G. Zhang , J. Lin , P. R. Chen , J. Am. Chem. Soc. 2019, 141, 17133–17141; 31580665 10.1021/jacs.9b05833

[4e] R. Das , R. F. Landis , G. Y. Tonga , R. Cao-Milán , D. C. Luther , V. M. Rotello , ACS Nano 2019, 13, 229–235; 30516966 10.1021/acsnano.8b05370 PMC6779054

[4f] S. Learte-Aymamí , C. Vidal , A. Gutiérrez-González , J. L. Mascareñas , Angew. Chem. Int. Ed. 2020, 59, 9149–9154; 10.1002/anie.202002032 32162393

[4g] J. Miguel-Ávila , M. Tomás-Gamasa , J. L. Mascareñas , Angew. Chem. Int. Ed. 2020, 59, 17628–17633; 10.1002/anie.202006689 PMC7689831 32627920

[4h] B. L. Oliveira , B. J. Stenton , V. B. Unnikrishnan , C. R. de Almeida , J. Conde , M. Negrão , F. S. S. Schneider , C. Cordeiro , M. G. Ferreira , G. F. Caramori , J. B. Domingos , R. Fior , G. J. L. Bernardes , J. Am. Chem. Soc. 2020, 142, 10869–10880; 32456416 10.1021/jacs.0c01622 PMC7304066

[4i] A. M. Pérez-López , B. Rubio-Ruiz , T. Valero , R. Contreras-Montoya , L. Álvarez de Cienfuegos , V. Sebastián , J. Santamaría , A. Unciti-Broceta , J. Med. Chem. 2020, 63, 9650–9659; 32787091 10.1021/acs.jmedchem.0c00781 PMC7497487

[4j] J. Ceballos , E. Grinhagena , G. Sangouard , C. Heinis , J. Waser , Angew. Chem. Int. Ed. 2021, 60, 9022–9031; 10.1002/anie.202014511 PMC8048981 33450121

[4k] C. Vidal , M. Tomás-Gamasa , P. Destito , F. López , J. L. Mascareñas , Nat. Commun. 2018, 9, 1913; 29765051 10.1038/s41467-018-04314-5 PMC5954130

[4l] M. Martínez-Calvo , J. R. Couceiro , P. Destito , J. Rodríguez , J. Mosquera , J. L. Mascareñas , ACS Catal. 2018, 8, 6055–6061. 30018848 10.1021/acscatal.8b01606 PMC6038097

[5a] H. C. Kolb , M. G. Finn , K. B. Sharpless , Angew. Chem. Int. Ed. 2001, 40, 2004–2021; 10.1002/1521-3773(20010601)40:11<2004::AID-ANIE2004>3.0.CO;2-5 11433435

[5b] C. W. Tornøe , C. Christensen , M. Meldal , J. Org. Chem. 2002, 67, 3057–3064. 11975567 10.1021/jo011148j

[6a] J. E. Hein , V. V. Fokin , Chem. Soc. Rev. 2010, 39, 1302–1315; 20309487 10.1039/b904091a PMC3073167

[6b] V. K. Tiwari , B. B. Mishra , K. B. Mishra , N. Mishra , A. S. Singh , X. Chen , Chem. Rev. 2016, 116, 3086–3240; 26796328 10.1021/acs.chemrev.5b00408

[6c] D. Schulz , A. Rentmeister , ChemBioChem 2014, 15, 2342–2347; 25224574 10.1002/cbic.201402240

[6d] F. Musumeci , S. Schenone , A. Desogus , E. Nieddu , D. Deodato , L. Botta , Curr. Med. Chem. 2015, 22, 2022–2050; 25895896 10.2174/0929867322666150421110819

[6e] J. Matyašovský , P. Perlikova , V. Malnuit , R. Pohl , M. Hocek , Angew. Chem. Int. Ed. 2016, 55, 15856–15859; 10.1002/anie.201609007 PMC6680173 27879047

[7] V. Hong , N. F. Steinmetz , M. Manchester , M. G. Finn , Bioconjugate Chem. 2010, 21, 1912–1916. 10.1021/bc100272z PMC3014321 20886827

[8] D. C. Kennedy , C. S. McKay , M. C. B. Legault , D. C. Danielson , J. A. Blake , A. F. Pegoraro , A. Stolow , Z. Mester , J. P. Pezacki , J. Am. Chem. Soc. 2011, 133, 17993. 21970470 10.1021/ja2083027

[9] For the use of stabilizing ligands to improve efficiency and reduce toxicity, see:

[9a] V. O. Rodionov , S. I. Presolski , D. D. Diaz , V. V. Fokin , M. G. Finn , J. Am. Chem. Soc. 2007, 129, 12705–12712; 17914817 10.1021/ja072679d

[9b] C. Besanceney-Webler , H. Jiang , T. Zheng , L. Feng , D. Soriano del Amo , W. Wang , L. M. Klivansky , F. L. Marlow , Y. Liu , P. Wu , Angew. Chem. Int. Ed. 2011, 50, 8051–8056; 10.1002/anie.201101817 PMC3465470 21761519

[9c] C. Uttamapinant , A. Tangpeerachaikul , S. Grecian , S. Clarke , U. Singh , P. Slade , K. R. Gee , A. Y. Ting , Angew. Chem. Int. Ed. 2012, 51, 5852–5856; 10.1002/anie.201108181 PMC3517120 22555882

[9d] J. C. Jewett , C. R. Bertozzi , Chem. Soc. Rev. 2010, 39, 1272–1279. 20349533 10.1039/b901970g PMC2865253

[10] For recent examples of alternative Cu catalysts for CuAAC, see:

[10a] Y. Bai , X. Feng , H. Xing , Y. Xu , B. K. Kim , N. Baig , T. Zhou , A. A. Gewirth , Y. Lu , E. Oldfield , S. C. Zimmerman , J. Am. Chem. Soc. 2016, 138, 11077–11080; 27529791 10.1021/jacs.6b04477

[10b] J. Chen , J. Wang , Y. Bai , K. Li , E. S. Garcia , A. L. Ferguson , S. C. Zimmerman , J. Am. Chem. Soc. 2018, 140, 13695–13702; 30192530 10.1021/jacs.8b06875

[10c] J. Chen , J. Wang , K. Li , Y. Wang , M. Gruebele , A. L. Ferguson , S. C. Zimmerman , J. Am. Chem. Soc. 2019, 141, 9693–9700; 31124359 10.1021/jacs.9b04181 PMC13229381

[10d] J. Chen , K. Li , J. S. L. Shon , S. C. Zimmerman , J. Am. Chem. Soc. 2020, 142, 4565–4569; 32100539 10.1021/jacs.9b13997 PMC11446247

[10e] Q. Lu , S. Bai , Z. Chen , N. Zheng , X. Feng , Y. Bai , ACS Mater. Lett. 2020, 2, 89–94;

[10f] J. Clavadetscher , S. Hoffmann , A. Lilienkampf , L. Mackay , R. M. Yusop , S. A. Rider , J. J. Mullins , M. Bradley , Angew. Chem. Int. Ed. 2016, 55, 15662–15666; 10.1002/anie.201609837 27860120

[10g] F. Wang , Y. Zhang , Z. Liu , Z. Du , L. Zhang , J. Ren , X. Qu , Angew. Chem. Int. Ed. 2019, 58, 6987–6992; 10.1002/anie.201901760 30888728

[10h] Y. You , F. Cao , Y. Zhao , Q. Deng , Y. Sang , Y. Li , K. Dong , J. Ren , X. Qu , ACS Nano 2020, 14, 4178–4187; 32298078 10.1021/acsnano.9b08949

[10i] J. Huang , L. Wang , P. Zhao , F. Xiang , J. Liu , S. Zhang , ACS Catal. 2018, 8, 5941–5946;

[10j] S. Li , L. Wang , F. Yu , Z. Zhu , D. Shobaki , H. Chen , M. Wang , J. Wang , G. Qin , U. J. Erasquin , L. Ren , Y. Wang , C. Cai , Chem. Sci. 2017, 8, 2107–2114; 28348729 10.1039/c6sc02297a PMC5365239

[10k] L. Xue , Y. Yang , S. Wu , Y. Huang , J. Li , Y. Xiang , G. Li , Anal. Chem. 2020, 92, 2972–2978; 31973521 10.1021/acs.analchem.9b03677

[10l] J. Miguel-Ávila , M. Tomás-Gamasa , A. Olmos , P. Perez , J. L. Mascareñas , Chem. Sci. 2018, 9, 1947–1952. 29675241 10.1039/c7sc04643j PMC5892125

[11] P. Destito , J. R. Couceiro , H. Faustino , F. López , J. L. Mascareñas , Angew. Chem. Int. Ed. 2017, 56, 10766–10770; 10.1002/anie.201705006 PMC5638077 28685950

[12] W. G. Kim , M. E. Kang , J. B. Lee , M. H. Jeon , S. Lee , J. Lee , B. Choi , P. M. S. D. Cal , S. Kang , J.-M. Kee , G. J. L. Bernardes , J.-U. Rohde , W. Choe , S. Y. Hong , J. Am. Chem. Soc. 2017, 139, 12121–12124. 28814075 10.1021/jacs.7b06338

[13a] Y. Liao , Q. Lu , G. Chen , Y. Yu , C. Li , X. Huang , ACS Catal. 2017, 7, 7529–7534;

[13b] W. Song , N. Zheng , M. Li , K. Dong , J. Li , K. Ullah , Y. Zheng , Org. Lett. 2018, 20, 6705–6709; 30346176 10.1021/acs.orglett.8b02794

[13c] W. Song , N. Zheng , M. Li , K. Ullah , Y. Zheng , Adv. Synth. Catal. 2018, 360, 2429–2434.

[14a] W. Song , N. Zheng , Org. Lett. 2017, 19, 6200–6203; 29112442 10.1021/acs.orglett.7b03123

[14b] R. Chen , L. Zeng , Z. Lai , S. Cui , Adv. Synth. Catal. 2019, 361, 989–994;

[14c] L. Zeng , Z. Lai , C. Zhang , H. Xie , S. Cui , Org. Lett. 2020, 22, 2220–2224; 32133860 10.1021/acs.orglett.0c00394

[14d] M. Li , N. Zheng , J. Li , Y. Zheng , W. Song , Green Chem. 2020, 22, 2394–2398.

[15] A. M. McNair , K. R. Mann , Inorg. Chem. 1986, 25, 2519–2527.

[16] For a photoinitiated CuAAC with temporal control, which so far has not been shown to be biocompatible, see:

[16a] B. J. Adzima , Y. Tao , C. J. Kloxin , C. A. DeForest , K. S. Anseth , C. N. Bowman , Nat. Chem. 2011, 3, 256–259; See also:21336334 10.1038/nchem.980

[16b] P. K. Sasmal , S. Carregal-Romero , W. J. Parak , E. Meggers , Organometallics 2012, 31, 5968–5970.

[17] Previous studies had indicated that cationic Cp*Ru^II^ complexes such as **Ru2** were not suitable catalysts for promoting azide–(thio)alkyne cycloaddition reactions in organic solvents:

[17a] B. C. Boren , S. Narayan , L. K. Rasmussen , L. Zhang , H. Zhao , Z. Lin , G. Jia , V. V. Fokin , J. Am. Chem. Soc. 2008, 130, 8923–8930; 18570425 10.1021/ja0749993

[17b] S. Ding , G. Jia , J. Sun , Angew. Chem. Int. Ed. 2014, 53, 1877–1880; 10.1002/anie.201309855 24474668

[18a] Complex **Ru2′** can be independently prepared in quantitative yield by mixing **Ru2** and the thioalkyne **2 a** in CH_2_Cl_2_ at room temperature. See the Supporting Information for its preparation, X-ray crystallographic analysis and a preliminary mechanistic proposal.

[18b] This type of (2+2+2) thioalkyne trimerization, promoted by **Ru2**, was not observed when the neutral Ru^II^ chloride complex **Ru1** was used.

[19] For the use of a chloride source in combination with **Ru2** in organic solvents, see:

[19a] J. A. Varela , S. G. Rubín , C. González-Rodríguez , L. Castedo , C. Saá , J. Am. Chem. Soc. 2006, 128, 9262–9263; 16848424 10.1021/ja057306w

[19b] J. A. Varela , L. Castedo , C. Saá , Org. Lett. 2003, 5, 2841–2844. 12889888 10.1021/ol0348710

[20] For precedent on CpRu oxo complexes, see:

[20a] R. Shimogawa , T. Takao , H. Suzuki , Organometallics 2014, 33, 289–301;

[20b] S. Takemoto , H. Ishii , M. Yamaguchi , A. Teramoto , M. Tsujita , D. Ozeki , H. Matsuzaka , Organometallics 2019, 38, 4298–4306;

[20c] K. M. Rao , C. L. Day , R. A. Jacobson , R. J. Angelici , Organometallics 1992, 11, 2303–2304;

[20d] L. Fan , M. L. Turner , M. B. Hursthouse , K. M. A. Malik , O. V. Gusev , P. M. Maitlis , J. Am. Chem. Soc. 1994, 116, 385–386;

[20e] J. A. Gilbert , D. S. Eggleston , W. R. Murphy, Jr. , D. A. Geselowitz , S. W. Gersten , D. J. Hodgson , T. J. Meyer , J. Am. Chem. Soc. 1985, 107, 3855–3864; for reviews of organic chemistry in/on water, see:

[20f] C.-J. Li , L. Chen , Chem. Soc. Rev. 2006, 35, 68–82; 16365643 10.1039/b507207g

[20g] V. Chanda , V. Fokin , Chem. Rev. 2009, 109, 725–748. 19209944 10.1021/cr800448q PMC3999525

[21] Analysis by ESI-MS of the mixtures resulting from dissolving **Ru2** in water allowed the detection of molecular ions corresponding to oxo derivates, [Cp*Ru(O)]^+^, [Cp*Ru(O)(MeCN)_2_] and [Cp*Ru(O)_2_(MeCN)_2_], as well as hydroxo species [{Cp*Ru(O)}_2_(OH)]^+^ (see the Supporting Information, Figure S11).

[22] L. Xu , S. L. Kuan , T. Weil , Angew. Chem. Int. Ed. 2021, 10.1002/anie.202012034; PMC8248073 33258535

[23] See the Supporting Information for further details.

[24] According to previous studies, the displacement is initiated by an η6 → η4 arene shift with concomitant addition of an external ligand, and subsequent release of the arene unit, see:

[24a] D. S. Perekalin , A. R. Kudinov , Coord. Chem. Rev. 2014, 276, 153–173;

[24b] D. S. Perekalin , N. V. Shvydkiy , Y. V. Nelyubina , A. R. Kudinov , Mendeleev Commun. 2015, 25, 29–31;

[24c] J. A. S. Howell , N. F. Ashford , D. T. Dixon , J. C. Kola , T. A. Albright , S. K. Kang , Organometallics 1991, 10, 1852–1864.

[25a] The use of anthracenyl azide **1 b** is also possible, but the irradiation must be performed immediately before the addition of **1 b**, to avoid the decomposition of the anthracenyl moiety by the light.

[25b] See Ref. [16b] for the use of **Ru4** in a photo-triggered *N*-allylcarbamatte cleavage.

[26] Control experiments confirmed that, under these dilute conditions the presence of acetonitrile as a cosolvent is detrimental for the activity of the photoactivatable catalysts **Ru4** and **Ru5**, probably owing to competing coordination to the Ru center, which slows down the rate of the process.

[27] In all cases, control experiments with photoactivatable catalysts **Ru4** and **Ru5**, without irradiation, provided negligible yields of triazole products.

[28] Control experiments with **Ru2** confirmed its low toxicity towards HeLa cells (see the Supporting Information). However, the use of **Ru4** and light under the conditions of the in vitro experiments (365 nm, 15 min) caused severe damage to the cells. Thus, in vivo applications will require specific optimization, the use of alternative two-photon irradiation settings or the development of visible-light-activatable Ru catalysts.

