# Supplementary material for: Bioorthogonal Azide–Thioalkyne Cycloaddition Catalyzed by Photoactivatable Ruthenium(II) Complexes
Source: Angew Chem Int Ed Engl. 2021 Jun 16;60(29):16059–66. doi: 10.1002/anie.202103645 (PMC9545742; doi:10.1002/anie.202103645)

Angewandte Chemie
Eine Zeitschrift der Gesellschaft Deutscher Chemiker

Supporting Information

Bioorthogonal Azide–Thioalkyne Cycloaddition Catalyzed by Photoactivatable Ruthenium(II) Complexes

Alejandro Gutiérrez-González, Paolo Destito, José R. Couceiro, Cibran Pérez-González, Fernando López, and José L. Mascareñas**

anie_202103645_sm_miscellaneous_information.pdf

Supporting Information

Bioorthogonal Azide–Thioalkyne Cycloadditions Catalyzed by Photoactivatable Ru(II) Complexes

Alejandro Gutiérrez-González[a], Paolo Destito[a], José R. Couceiro,[a] Cibrán Pérez-González,[a] Fernando López*[a,b] and José L. Mascareñas*[a]

[a] Centro Singular de Investigación en Química Biolóxica e Materiais Moleculares (CiQUS), Departamento de Química Orgánica. Universidade de Santiago de Compostela, 15782 Santiago de Compostela, Spain.

[b] Misión Biológica de Galicia, Consejo Superior de Investigaciones Científicas (CSIC), 36080, Pontevedra (Spain).

Table of Contents

1. General Information S3

2. Synthesis of Ru Complexes, Azides and Thioalkynes S4

3. General Procedure for the RuAtAC under Millimolar Conditions S9

4. General Procedure for the RuAtAC under Micromolar Conditions S15

5. Performance of **Ru1**- **Ru4** in the Reaction of **1d** and **2a** at Micromolar Conditions S17

6. Influence of the Catalyst Loading (**Ru2**) at Different Micromolar Concentrations S18

7. Influence of the Catalyst Loading (**Ru4**) at Different Micromolar Concentrations S18

8. Assesment of the Stability of **Ru4** in Biologically Relevant Media S19

9. Comparative of Ir, Rh and Ru complexes, under micromolar conditions S20

9 MS- Speciation Experiments S21

10 Labelling of Biomolecules S23

11. Experiments in the Presence of Cells S26

12. References S35

13. NMR Spectra S36

1. General Information

Procedures for the synthesis of precursors and complexes were performed under an atmosphere of dry N_2 using vacuum-line and standard Schlenk techniques. Dry solvents were directly purchased from Sigma Aldrich and used without further purification. Water used in the catalytic reactions was fresh Mili-Q grade. The abbreviation “rt” correspond to approximately 23 ºC. All reactions were stirred using Teflon-coated magnetic stirring bars. Flash chromatography was carried out in silica gel unless otherwise stated. Na_2SO_4 or $MgSO_4$ were used as drying agents. Reactions carried out with temperature control were performed using either Thermo watch-controlled silicone oil baths for heating or the corresponding bath for cooling (water-ice for 0ºC or acetone-dry ice for -78 ºC).

^{1}H, ^{13}C ,^{19}F and ^{31}P NMR spectra were collected on a 300 MHz (Varian), 400 MHz (Varian) or 500 MHz (Bruker and Varian) in $CDCl_3$, CD_2Cl_2, CD_3OD, DMSO-d_6 or DMF-d_7. Carbon types and structure assignments were determined from DEPT-NMR. NMR spectra were analyzed using MestreNova© NMR data processing software (www.mestrelab.com). Abbreviations to denote the multiplicity of the signals are s (singlet), d (doublet), t (triplet), q (quartet), quint (quintet), sex (sextet), m (multiplet) and their corresponding combinations. Routine mass spectra were acquired using ITMS Bruker AmaZon SL at CIQUS, High Resolution Mass Spectrawere recordedusing electrospray ionization (ESI) recorded at the CACTUS facility of the University of Santiago de Compostela or at the University of Vigo. HPLC-MS analysis was carried out using Bruker Amazon IT/MS with C18 column using coumarin (2H-chromen-2-one) as internal standard.

Cp*Ru(cod)Cl (**Ru1**) and [Cp*Ru(MeCN)$_3$]PF$_6$ (**Ru2**), were purchased from Sigma Aldrich and used without further purification. [Ir(cod)Cl]$_2$ and [Rh(CO)$_2$Cl]$_2$ were purchased from STREM and Sigma Aldrich and used a without further purification. Pyrene, sodium pyrene-1-sulfonate, naphtalene, phenylcetylene, but-1-yn-1-ylbenzene,1-heptyn-7-ol (**2e**), trimethylsilylacetylene, n-BuLi (2.5 M in hexanes), tetraethylammonium chloride, sodium azide, (4-bromobutyl) triphenylphosphonium bromide, Rhodamine-B, 3-(azidomethyl)anthracene, thionyl chloride, isopropyldisulfide, phenyldisulfide, α,α-dibromo-m-xylene, 4-dimethylaminobenzaldehyde were purchased from Sigma-Aldrich and used without further purification. Azides **1a**,[1] **1b**,[2] **1c**,[3] **1e**[4]; thioalkynes **2a**,[5] **2b**,[6] **2d**[7]; precursors **S1**,[8] **S2**[9]; triazole **3aa**[10] and ruthenium complexes **Ru3**,[11] **Ru4**[12] are known compounds, have been synthesized according to theor respective reported procedures and their NMR data was in accordance with that previously reported.

Reactions that required the photoactivation of the [Cp*Ru(arene)]X complexes were irradiated at 365 nm with a UV-B LED (Custom apparatus by ThorLabs) for the indicated time, using the following setup.

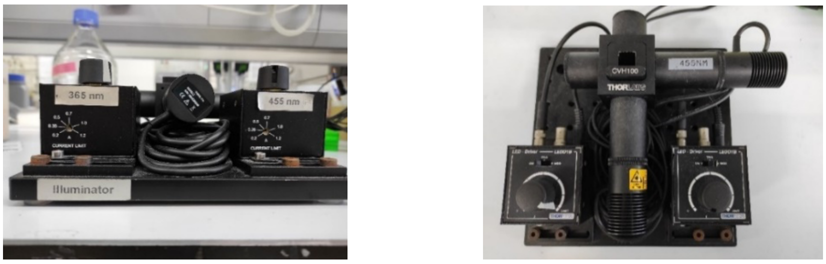

Front view Upper view

Figure S1. Irradiation set up for the reactions with Ru3-Ru5. Two UV-led, 365 nm and 455 nm (by ThorLabs)

2. Synthesis of Ru Complexes, Azides and Thioalkynes

2.1 Preparation of Ruthenium complexes

η5-(Pentamethyl-cyclopentadienyl)-η6-(naphtalene) ruthenium(II) tetraphenylborate (Ru3)

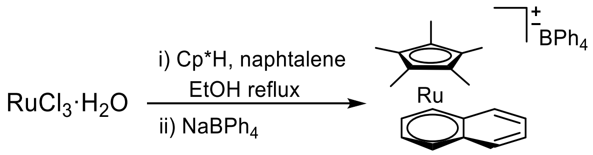

The synthesis of [Cp*Ru(Napht)]BPh_4 was carried out according to a reported procedure:[11] In a dried Schlenk tube, filled with nitrogen, $RuCl_3$·$3H_2O$ (100 mg, 0.482 mmol, 1 eq.) was suspended in EtOH (5 mL). The mixture was refluxed until the starting material was dissolved and 1,2,3,4,5-pentamethylcyclopenta-1,3-diene (Cp*H, 151 µL, 0.946 mmol, 2 eq.) and naphthalene (123.0 mg, 0.946 mmol, 2 eq.) were added to the reaction vessel. The resulting solution was refluxed overnight, the solvent was removed under vacuum and the residue was dissolved in a water/Et_2O mixture (1:1, 10 mL). The aqueous fraction was retained and washed with Et_2O (3 x 5 mL). The aqueous layer was mixed slowly with an aqueous solution of $NaBPh_4$ (5 mL, 0.30 M). The resulting precipitate was filtered and washed with Et_2O. If necessary, **Ru3** can be further purified through a short column of neutral alumina using acetone as eluent. Yellow- mustard coloured fractions were collected affording [Cp*Ru(naphthalene)]BPh_4 (**Ru3**) as yellow needles (94% yield). The NMR data are in accordance with those previously reported.[11] **^{1}H NMR** (300 MHz, DMSO-d_6) δ 7.73 – 7.61 (m, 4H), 7.22 – 7.11 (m, 8H), 6.92 (t, J = 7.3 Hz, 8H), 6.78 (t, J = 7.1 Hz, 4H), 6.71 (dd, J = 4.3, 2.4 Hz, 2H), 6.15 (dd, J = 4.3, 2.4 Hz, 2H), 1.61 (s, 15H).

η^5-(Pentamethyl-cyclopentadienyl)-η^6-(pyrene) ruthenium(II) hexafluorophosphate (Ru4)

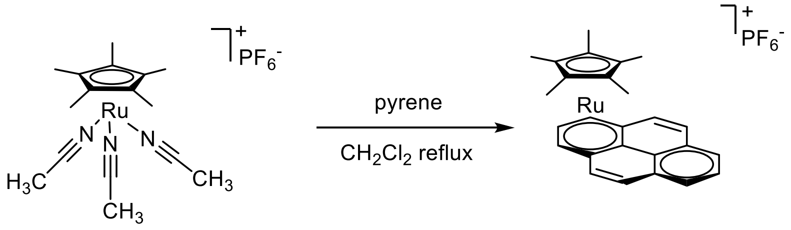

The synthesis of [Cp*Ru(pyrene)]PF_6 (**Ru4**) was carried out according a reported procedure:[12] In an dried Schlenk tube, filled with N_2, [Cp*Ru(MeCN)$_3$]PF_6 (100 mg, 0.19 mmol, 1.0 eq.) was added to a solution of pyrene (40.6 mg, 0.20 mmol, 1.05 eq.) in degassed 1,2-dichloroethane (5 mL). The mixture was gently heated (just below the refluxing temperature) for 20 h, the solvent was removed, and the product was chromatographed on neutral alumina using Et_2O as eluent, collecting the yellow fractions. Concentration under vacuum gave [Cp*Ru(pyrene)]PF_6 (**Ru4**) as bright yellow needles (40 mg, 36% yield). The NMR data is in accordance with that previously reported.[12] **^{1}H NMR** (300 MHz, CD_2Cl_2) δ 8.26 – 8.05 (m, 5H), 7.55 (dd, J = 9.3, 4.2 Hz, 2H), 6.41 (d, J = 6.0 Hz, 2H), 6.12 (t, J = 6.0 Hz, 1H), 1.32 (s, 15H).

η5-(Pentamethylcyclopentadienyl)-η6-(pyrene-1-sulfonate) ruthenium (II) sodium hexafluorophosphate (Ru5).

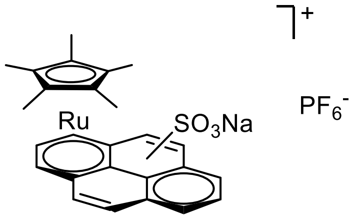

The synthesis of (**Ru5**) was carried out adapting the previous procedure for the synthesis of **Ru4**:[12] Sodium pyrene-1-sulfonate (60.6 mg, 0.2 mmol) was suspended in a degassed THF:H_2O (9:1) mixture and [Cp*Ru(MeCN)$_3$]PF_6 (100 mg, 0.2 mmol) was added. The reaction mixture was refluxed for 14 h, cooled down to rt and the resulting precipitate was filtered and washed with CH_2Cl_2 and Et_2O. The resulting pale green powder was dried under vacuum and used without further purification (41.1 mg 30% yield). If needed, **Ru5** can be purified by recrystallization in MeOH:CH_2Cl_2 and slow diffusion of Et_2O. **Ru5** consists of a ca.1:1 mixture of positional isomers. **^{1}H NMR** (300 MHz, Methanol-*d*$_4$) δ 9.13 (d, *J* = 9.7 Hz, 1H), 8.62 (t, *J* = 8.7 Hz, 2H), 8.30 – 8.02 (m, 7H), 7.61 (d, *J* = 9.7 Hz, 1H), 7.51 (dd, *J* = 9.3, 7.2 Hz, 2H), 6.77 (d, *J* = 6.2 Hz, 1H), 6.58 (d, *J* = 6.2 Hz, 1H), 6.47 (dd, *J* = 14.0, 6.0 Hz, 2H), 6.14 (t, *J* = 6.0 Hz, 1H), 1.28 (m, 30H). **^{13}C NMR** (75 MHz, Methanol-*d*$_4$) δ 143.5, 133.9, 133.6, 133.0, 132.7, 132.0, 130.3, 129.7, 129.1, 128.7, 126.6, 126.5, 126.2, 125.6, 125.3, 125.1, 124.8, 94.7, 94.6, 94.1, 93.9, 90.9, 88.8, 87.9, 86.6, 86.4, 85.3, 9.0, 8.7. **HRMS-ESI** calculated for $C_{26}H_{25}O_3S^+$ 519.0562 found 519.0562.

Preparation of dithiofulvene Cp*Ru(II) complex Ru2’

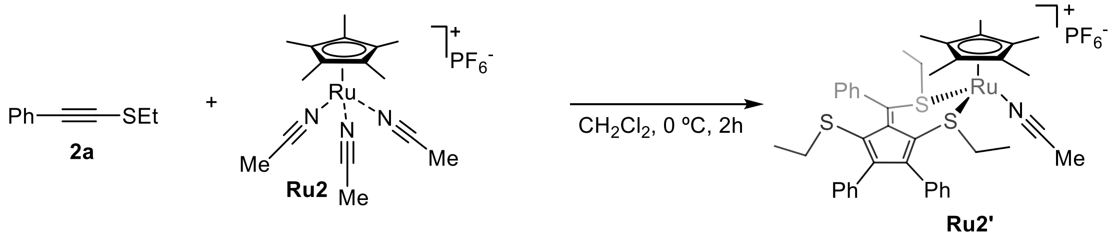

Cp*Ru complex **Ru2’** was first detected from the reactions carried out in CH_2Cl_2 between **1a** and **2a**, promoted by **Ru2** (Table 1 main manuscript, entry 2). Nonetheless, **Ru2’** can be quantitatively prepared following the subsequent procedure: In a dried Schlenk tube, under nitrogen, **Ru2** (15.0 mg, 31.7 μmol) was dissolved in freshly distilled, degassed CH_2Cl_2 (1 mL) and stirred at 0 ºC for 15 min. To the resulting pale-orange solution, thioalkyne **2a** (21 μL, 20.6 mg, 127 μmol) was added via syringe, leading to a dark green solution. The mixture was stirred at 0 ºC for 2 h, resulting in a dark orange-brown solution. Then, 6 mL of dried, degassed pentane were added slowly (in order to avoid mixing), and the resulting mixture was kept at -28 ºC for 3 days, to yield the entitled compound (**Ru2’**) as deep-orange- crystalline needles (99% yield). **^{1}H NMR** (300 MHz, Methylene Chloride-*d*$_2$) δ 7.90 – 7.82 (m, 1H), 7.71 (q, *J* = 4.7 Hz, 1H), 7.64 – 7.56 (m, 2H), 7.54 – 7.05 (m, 11H), 2.96 – 2.80 (m, 2H), 2.61 – 2.44 (m, 2H), 2.40 (d, *J* = 1.7 Hz, 3H), 1.72 (s, 15H), 1.70 – 1.61 (m, 3H), 1.17 (t, *J* = 7.4 Hz, 3H), 0.84 (t, *J* = 7.4 Hz, 2H), 0.59 (t, *J* = 7.2 Hz, 3H).**^{13}C NMR** (75 MHz, CD_2Cl_2) δ 156.1 (C), 151.1 (C), 149.6 , (C) 147.1 (C), 140.9 (C), 136.7 (C), 136.2 (C), 135.5 (CH), 133.2 (CH), 132.2 (CH), 131.7 (CH), 131.2 (CH), 131.0 (CH), 130.0 (CH), 129.7 (CH), 129.6 (CH), 129.5 (CH), 129.1 (C), 89.6 (C), 38.2 (CH_2), 37.2 (CH_2), 31.3 (CH_2), 15.9 (CH_3), 14.8 (CH_3), 14.4 (CH_3), 11.0 (CH_3), 10.5 (CH_3), 10.4 (CH_3), 5.8 (CH_3). **HRMS-ESI** calculated for $C_{40}H_{45}RuS_3^+$ 723.1730 found 723.1727.

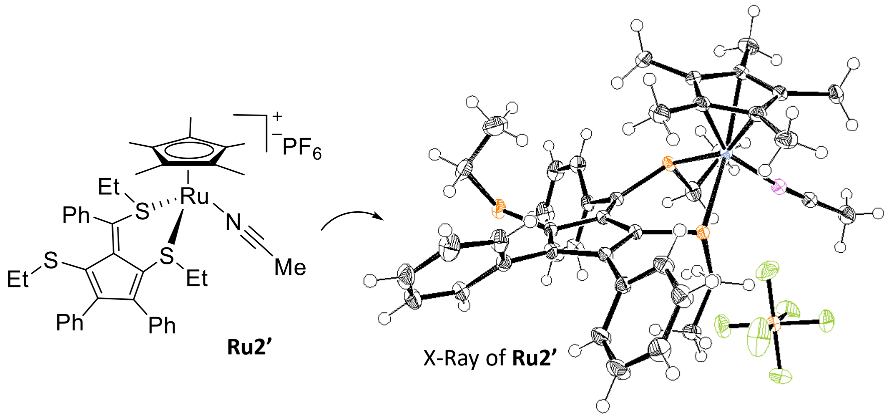

Figure S2. **X-ray structure of Ru2'**; (CCDC 2070106) [**Note**: PF_6 is distorted within the crystalline structure, only one of the mayor contributions is shown for clarity. A molecule of CH_2Cl_2 is also present in the cell and was omitted for clarity].

X-ray Crystal Structure Analysis of Ru2': $C_{43}H_{50}Cl_2F_6NPRuS_3$, *MW*= 994.00 g/mol, orange-needles, crystal size 0.039 mm x 0.055 mm x 0.341 mm, monoclinic, space group *P21/n* , a = 19.4000(7) Å, b = 9.9869(4) Å, c = 23.9334(9) Å, β = 107.4067(14)°, V = 4424.6(3) Å^3, T = 100 K, Z = 4, D_{calc} = 1.492 g/cm^3, λ = 0.71073 Å, Gaussian absorption correction (T_{mn} = 0.89, T_{max} = 0.97) Bruker D8 VENTURE PHOTON-III C14 κ-geometry diffractometer, 4.400° < 2θ < 57.40°, 166840 measured reflections, 14775 independent reflections 10854 reflections with $I > 2\sigma(I)$, R_{int} = 0.087. The structure was solved by direct methods and refined by full-matrix least-squares against F^2 to R [$F^2 > 2\sigma(F^2)$] = 0.038, wR(F^2)= 0.095, 614 parameters. The H atoms were inferred from neighbouring sites, H-atom parameters constrained, $w = 1/[\sigma^2(F_o^2) + (0.033P)^2 + 4.3237P]$ where $P = (F_o^2 + 2F_c^2)/3$, $(\Delta/\sigma)_{max}$ = 0.001, $\Delta\rho_{max}$ = 0.52 e Å^{-3} $\Delta\rho_{min}$ = −0.82 e Å^{-3}.

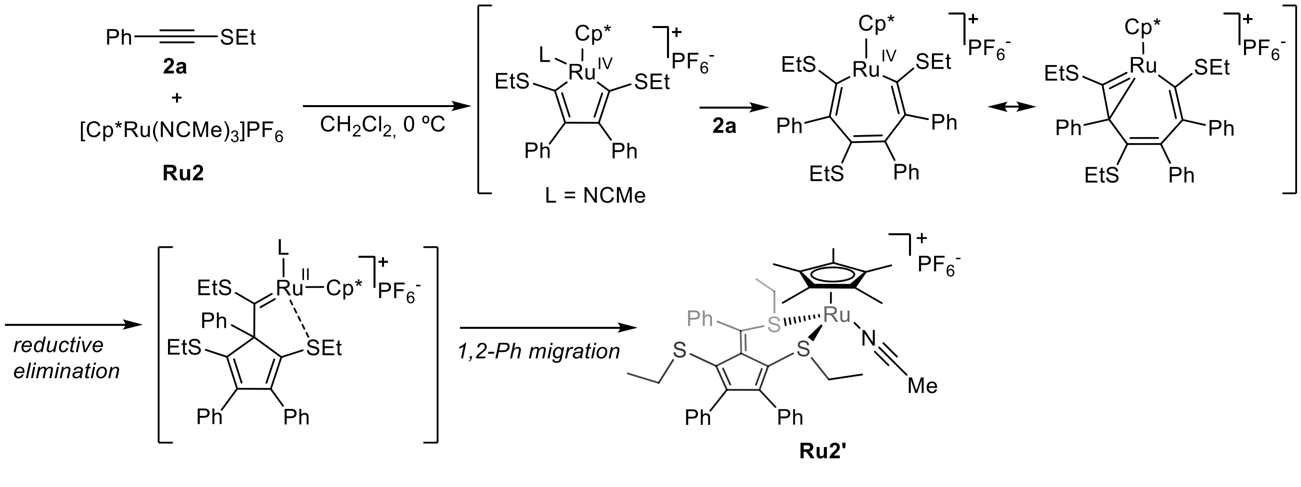

Scheme S1. Tentative mechanistic proposal for the formation of **Ru2'**.

2.2 Preparation of organic azides

Azides **1a**,[1] **1b**[2], **1c**[3] and **1d**[4] are known compounds and were prepared according to reported procedures.

3-(Azidomethyl)benzyl) triphenylphosphonium bromide (1d)

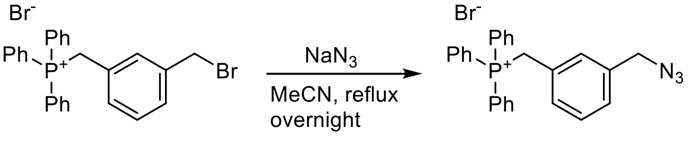

S1 **1d**

NaN_3 (241 mg, 3.70 mmol, 1.3 eq.) was added to a solution of (3-(bromomethyl) benzyl) triphenylphosphonium bromide[8] (1.50 g, 2.85 mmol, 1.0 eq.) in MeCN (30 mL) and DMF (5 mL), and the mixture was stirred under reflux overnight. Then, the solvent was evaporated and the residue was purified by flash column chromatography [CH_2Cl_2:MeOH (95:5) as eluent] to obtain the corresponding azide (**1d**) as a light yellow powder (1.067 g, 77% yield). **^{1}H NMR** (300 MHz, Chloroform-*d*) δ 7.8 – 7.6 (m, 15H), 7.2 (s, 3H), 7.0 (s, 1H), 5.5 (d, *J* = 14.5 Hz, 2H), 4.1 (s, 2H). **^{13}C NMR** (75 MHz, Chloroform-d) δ 135.8 (d, *J* = 3.3 Hz, C), 134.9 (d, *J* = 2.8 Hz, CH), 134.2 (d, *J* = 9.8 Hz, CH), 131.3 (d, *J* = 5.4 Hz, CH), 131.1 (d, *J* = 5.4 Hz, CH), 130.1 (d, *J* = 12.6 Hz, CH), 129.2 (d, *J* = 3.2 Hz, CH), 128.1 (d, *J* = 3.7 Hz, CH), 127.8 (d, *J* = 8.6 Hz, C), 117.3 (d, *J* = 85.8 Hz, C), 53.9 (CH_2), 30.5 (d, *J* = 47.0 Hz, CH_2). **HRMS-ESI** Calculated for $C_{36}H_{33}N_3PS^+$ 570.2127 found 570.2129.

2.3 Preparation of thioalkyne partners

Thioalkynes **2a**,[5] **2b**[6] y **2d**[7] are known compounds and were prepared according to reported procedures.

Benzyl(5-phenylpent-1-yn-1-yl)sulfane (2c)

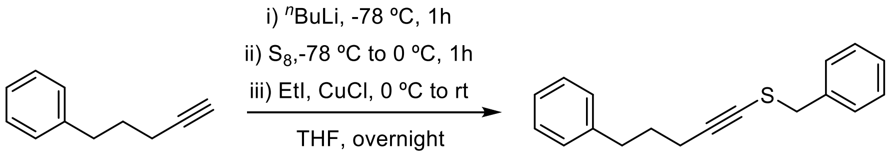

2c

Pent-4-yn-1-ylbenzene (1.6 mL, 10.2 mmol, 1.0 eq.) and freshly distilled THF (30 mL) were successively added to a two-neck round-bottom flask and the mixture was cooled to -78 ºC. Then, *n*-BuLi (4.3 mL, 2.5 M in hexane, 10.7 mmol, 1.05 eq.) was added dropwise and the mixture was allowed to warm up to 0 ºC (water/ice bath) and stirred for 30 min. Elemental sulphur (0.327g, 1.27 mmol, 0.125 eq.) was then added in one-portion, and the mixture turned from yellow to deep red, and was stirred for an additional hour. CuCl (50 mg, 5 mol%) and benzylbromide (1.74 g, 1.2 mL, 1.0 eq.) were then added and the solution was allowed to warm up to rt and stirred overnight. The reaction was quenched by addition of $NH_4Cl_{(sat)}$, extracted with Et_2O, dried and evaporated to dryness. The resulting crude was purified by flash column chromatography using hexane as eluent to yield benzyl(5-phenylpent-1-yn-1-yl)sulfane (**2c**) as a colourless oil (1.01 g, 3.8 mmol, 50% yield). **^{1}H NMR** (300 MHz, Chloroform-*d*) δ 7.52 – 6.87 (m, 10 H), 3.87 (s, 2 H), 2.84 – 2.40 (t, *J* = 6.9 Hz, 2H), 2.25 (t, *J* = 6.9 Hz, 2H), 1.75 (p, *J* = 7.0 Hz, 2H). **^{13}C NMR** (75 MHz, Chloroform-*d*) δ 141.7 (C), 137.1 (C), 129.1 (CH), 128.6 (CH), 128.5

(CH), 127.7 (CH), 126.0 (CH), 95.6 (C), 68.8 (C), 40.3 (CH_2), 34.8 (CH_2), 30.3 (CH_2), 19.6 (CH_2). **HRMS-ESI** Calculated for $C_{18}H_{19}S$ 267.1202, found 267.1203.

N-(6-(Diethylamino)-9-(2-(((7-(ethylthio)hept-6-yn-1-yl)oxy) carbonyl)phenyl)- 3H-xanthen-3-ylidene)-N-ethylethanaminium chloride (2i)

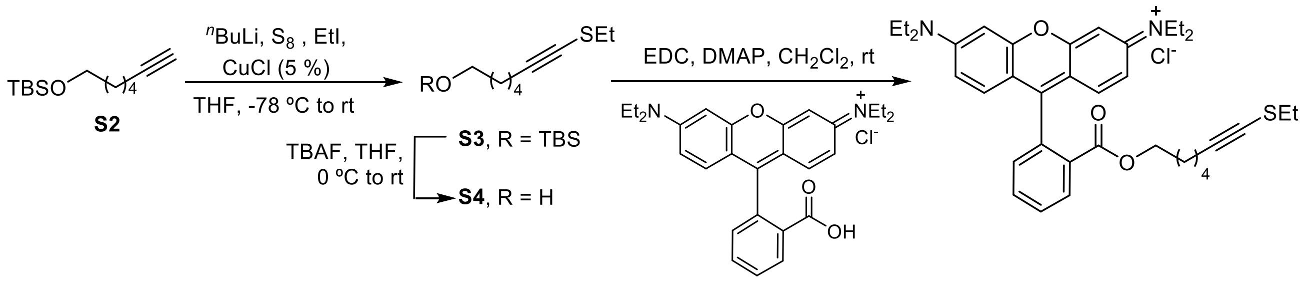

tert-Butyl((7-(ethylthio)hept-6-yn-1-yl)oxy)dimethylsilane (**S3**), was prepared according to the above described procedure for **2c** (1.76 g, 70% yield). **^{1}H NMR** (300 MHz, Chloroform-*d*) δ 3.57 (t, *J* = 6.2 Hz, 2H), 2.63 (q, *J* = 7.3 Hz, 2H), 2.27 (t, *J* = 6.8 Hz, 2H), 1.58 – 1.38 (m, 6H), 1.33 (t, *J* = 7.3 Hz, 3H), 0.86 (s, 9H), 0.01 (s, 6H).**^{13}C NMR** (75 MHz, Chloroform-*d*) δ 94.6 (C), 68.1(C), 63.1 (CH_2), 32.4 (CH_2), 29.5 (CH_2), 28.7 (CH_2), 26.0 (CH_3), 25.2 (CH_2), 20.2 (CH_2), 18.4 (C), 14.7 (CH_3), -5.2 (CH_3). **HRMS-ESI** Calculated for $C_{15}H_{31}OSSi$ 287.1859 found 287.1859. TBAF (6.23 mL, 1.0 M in THF, 6.23 mmol, 1.05 eq.) was added to a solution of *tert*-butyl((7-(ethylthio)hept-6-yn-1-yl)oxy)dimethylsilane (**S3**, 1.70 g, 5.93 mmol) in THF (10 mL) at 0 ºC, and the mixture was stirred at 0 ºC for 15 min, allowed to warm to rt and stirred until full conversion was observed by TLC. Upon completion the mixture was poured into NH_4Cl (sat) (50 mL) and extracted with Et_2O. The organic phases were dried and evaporated to dryness to yield a crude residue that was column-chromatographed (from 0 to 20% Hexanes:EtOAc). Thus, 7-(ethylthio)hept-6-yn-1-ol (**S4**) was obtained as colourless oil (789 mg, 4.68 mmol, 79% yield). **^{1}H NMR** (300 MHz, $CDCl_3$) δ 3.65 (t, *J* = 6.4 Hz, 2H), 2.68 (q, *J* = 7.3 Hz, 2H), 2.33 (t, *J* = 6.7 Hz, 2H), 1.63 – 1.31 (m, 10H). **^{13}C NMR** (75 MHz, $CDCl_3$) δ 94.6 (C), 68.2 (C), 62.7 (CH_2), 32.2 (CH_2), 29.6 (CH_2), 28.6 (CH_2), 25.0 (CH_2), 20.1 (CH_2), 14.7 (CH_3). **HRMS-ESI** Calculated for $C_9H_{17}OS$ 173.0995 found 173.0994.

Rhodamine B (400 mg, 0.835 mmol, 1.0 eq.), EDC (176.1 mg, 0.919 mmol, 1.1 eq.), DMAP (25.5mg, 0.209, 0.25 eq.) and 7-(ethylthio)hept-6-yn-1-ol (**S4**, 158 mg, 0.919 mmol, 1.1 eq.) were added to a dried round-bottom flask containing CH_2Cl_2 (8.3 mL) at 0 ºC . The mixture was stirred at rt overnight, poured in of HCl 1N, extracted with CH_2Cl_2 (3 x 10 mL) and successively washed with $NaHCO_3$(sat) and brine. The organic layer was dried, evaporated and purified by flash column chromatography, using CH_2Cl_2:MeOH (95:5) as eluent, to yield N-(6-(diethylamino)-9-(2-(((7-(ethylthio)hept-6-yn-1-yl)oxy)carbonyl)phenyl)-3H-xanthen-3-ylidene)-N-ethylethanaminium chloride **2i** (318 mg, 60% yield). **^{1}H NMR** (500 MHz, Chloroform-*d*) δ 8.26 (d, *J* = 7.82, 1H), 7.79 (td, *J* = 7.5, 1.4 Hz, 1H), 7.72 (td, *J* = 7.7, 1.3 Hz, 1H), 7.28 (dd, *J* = 7.6, 0.9 Hz, 1H), 7.06 (d, *J* = 9.5 Hz, 2H), 6.91 – 6.85 (m, 2H), 6.81 (d, *J* = 2.5 Hz, 2H), 4.00 (t, *J* = 6.5 Hz, 2H), 3.62 (q, *J* = 7.1 Hz, 8H), 2.63 (q, *J* = 7.3 Hz, 2H), 2.20 (m, 4H), 1.41 (m, 4H), 1.31 (t, *J* = 7.3 Hz, 12H), 1.27 – 1.16 (m, 3H). **^{13}C NMR** (75 MHz, Chloroform-*d*) δ 165.2 (C), 159.0 (C), 157.8 (C), 155.6 (C), 133.5 (C), 133.1 (CH), 131.4 (CH), 130.5 (CH), 130.2 (CH), 114.3 (CH), 113.6 (C), 96.4 (CH), 94.17 (C), 70.6 (CH_2), 68.6 (C), 65.5 (CH_2), 46.2 (CH_2), 31.0 (CH_3), 29.6 (CH_2), 28.3 (CH_2), 27.9 (CH_2), 25.1 (CH_2), 20.0 (CH_2), 14.7 (CH_3), 12.7 (CH_3). **HRMS-ESI** Calculated for $C_{37}H_{45}N_2O_3S^+$ 597.3145 found 597.3145.

3. General Procedure for the RuAtAC under Millimolar Conditions

3.1 RuAtAC promoted by Ru2 in CH_2Cl_2 (exemplified for the reaction of **1a** and **2a**)

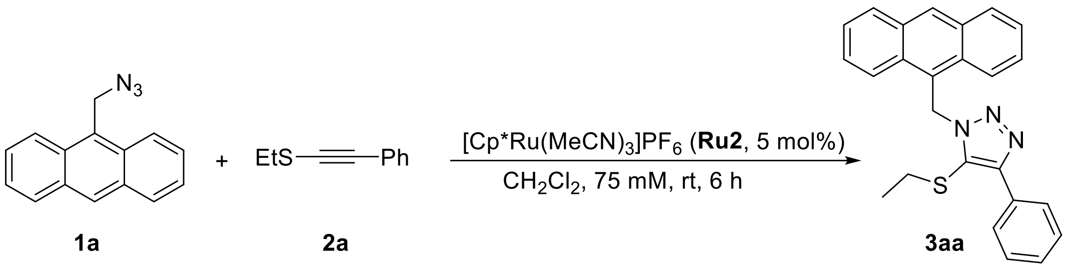

Ru2 (1.9 mg, 3.8 µmol), thioalkyne **2a** (24.3 mg 150 µmol), CH_2Cl_2 (1 mL), and azide **1a** (17.5 mg, 75 µmol), were sequentially added to a dry vial under argon at rt. The mixture was stirred for 6 h, filtered through a Florisil plug, concentrated and analysed by NMR using trimethoxybenzene as internal standard (IS). The resulting product, **3aa**, was obtained in 30% yield. [**Note**: When the reaction is carried out for 1 h, under otherwise identical reaction conditions, **3aa** is obtained in 15% yield]. NMR data of **3aa** is in agreement with that previously reported.[10] **^{1}H NMR** (500 MHz, Chloroform-*d*) δ 8.54 (d, *J* = 8.1 Hz, 3H), 8.14 (d, *J* = 7.3 Hz, 2H), 8.04 (d, *J* = 7.7 Hz, 2H), 7.62 – 7.54 (m, 2H), 7.53 – 7.45 (m, 2H), 7.45 – 7.38 (m, 2H), 7.41 – 7.30 (m, 1H), 6.58 (s, 2H), 2.36 (q, *J* = 7.5 Hz, 2H), 0.94 (t, *J* = 7.4 Hz, 3H).

3.2 RuAtAC promoted by Ru2 in water (exemplified for the cycloaddition of **1a** and **2a**)

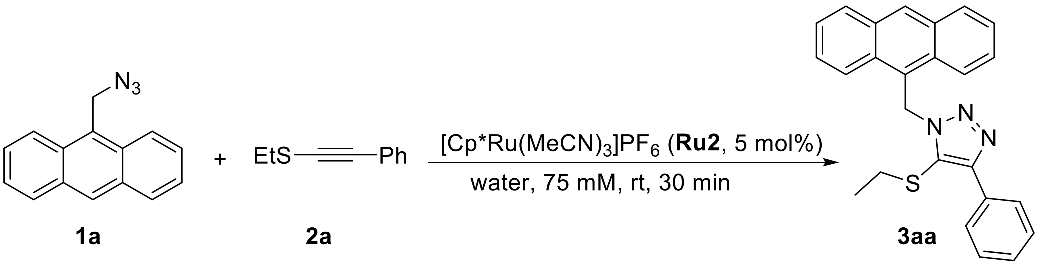

Ru2 (1.9 mg, 3.8 µmol), thioalkyne **2a** (24.3 mg, 150 µmol), H_2O (1 mL), and azide **1a** (17.5 mg, 75 µmol), were sequentially added under air to a vial. After 30 min, the reaction mixture was extracted with CH_2Cl_2 filtered through a Florisil plug, concentrated, and analysed by NMR using trimethoxybenzene as internal standard. The resulting product, **3aa**,[10] was obtained in 99% yield.

5-(Ethylthio)-4-phenyl-1-(p-methylbenzyl)-1H-1,2,3-triazole (3ba)

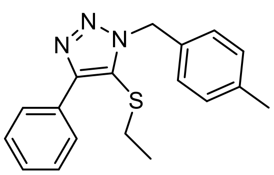

3ba

79 yield. **Rf** = 0.35 in 60:40 (Hexanes:Et_2O); flash column chromatography in Hexanes:Et_2O (from 80:20 to 30:70). **^{1}H NMR** (300 MHz, Chloroform-*d*) δ **^{1}H NMR** (300 MHz, Chloroform-*d*) δ 8.18 (d, *J* = 7.6 Hz, 2H), 7.44 (t, *J* = 7.5 Hz, 3H), 7.25 (d, *J* = 7.5 Hz, 2H), 7.14 (d, *J* = 7.8 Hz, 2H), 5.65 (s, 2H), 2.44 (q, *J* = 7.4 Hz, 2H), 2.32 (s, 3H), 0.97 (t, *J* = 7.4 Hz, 3H). **^{13}C NMR** (75 MHz, Chloroform-*d*) δ 149.1 (C), 138.1 (C), 132.4 (C), 130.8 (C), 129.5 (CH), 128.5 (CH), 128.3 (CH), 127.8 (CH), 126.8 (CH), 125.3 (C), 51.8 (CH_2) 30.0 (CH_2), 21.2 (CH_3), 14.4 (CH_3). **HRMS-ESI** Calculated for $C_{18}H_{19}N_3S^+$ 310.1373 found 310.1372.

(4-(5-(Ethylthio)-4-phenyl-1H-1,2,3-triazol-1-yl)butyl)triphenylphosphonium bromide (3ca)

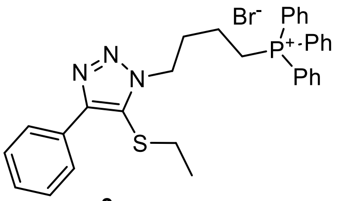

3ca

98% yield (carried out in a 9:1 Water: DMSO mixture). **Rf** = 0.39 in CH_2Cl_2:MeOH (90:10); flash column chromatography in CH_2Cl_2:MeOH (from 98:2 to 90:10) as eluent. **^{1}H NMR** (300 MHz, Chloroform-*d*) δ 8.12 (d, *J* = 7.5 Hz, 2H), 7.83 (dd, *J* = 12.4, 7.8 Hz, 6H), 7.69 (dd, *J* = 16.4, 5.5 Hz, 9H), 7.49 – 7.31 (m, 3H), 4.68 (t, *J* = 5.1 Hz, 2H), 4.18 – 4.02 (m, 2H), 2.68 (q, *J* = 7.5 Hz, 2H), 2.50 – 2.32 (m, 2H), 1.68 – 1.54 (m, 2H), 1.08 (t, *J* = 7.3 Hz, 3H). **^{13}C NMR** (75 MHz, Chloroform-*d*) δ 148.6 (C), 134.9 (CH), 133.7 (d, *J* = 10.1 Hz, CH), 130.5 (d, *J* = 12.5 Hz, CH), 128.6 (CH), 128.4 (CH), 126.7 (CH), 125.3 (C), 118.7 (C), 117.6 (C), 46.8 (CH_2), 30.4 (CH_2), 29.7 (d, *J* =17.3 Hz, CH_2), 21.4 (d, *J* = 50.8 Hz, CH_2), 19.2 (CH_2), 14.6 (CH_3). **HRMS-ESI** Calculated for $C_{32}H_{33}N_3PS^+$ 522.2127 found 522.2125.

(3-((5-(Ethylthio)-4-phenyl-1H-1,2,3-triazol-1-yl) methyl)benzyl) triphenyl-phosphonium bromide (3da)

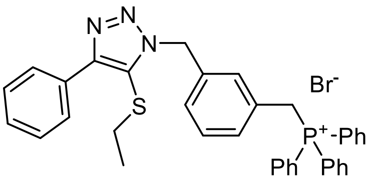

3da

69% yield (carried out in a 9:1 Water: DMSO mixture). **Rf** = 0.24 in CH_2Cl_2: MeOH (95:5); flash column chromatography in CH_2Cl_2:MeOH (from 100:0 to 95:5) as eluent. **^{1}H NMR** (300 MHz, Chloroform-*d*) δ 8.18 (d, *J* = 7.1 Hz, 2H), 7.75 – 7.63 (m, 9H), 7.63 – 7.54 (m, 6H), 7.53 – 7.34 (m, 3H), 7.23 – 7.08 (m, 3H), 7.01 (s, 1H), 5.53 – 5.36 (m, 4H), 2.52 (q, *J* = 7.4 Hz, 2H), 1.01 (t, *J* = 7.4 Hz, 3H). **^{13}C NMR** (75 MHz, Chloroform-*d*) δ 148.6 (C), 136.0 (d, *J* = 3.2 Hz, C), 135.1 (d, *J* = 2.6 Hz, CH) 134.2 (d, *J* = 9.8 Hz, CH), 131.7 (d, *J* = 5.4 Hz, CH), 130.6 (C), 130.5 (d, *J* = 5.4 Hz, CH), 130.2 (d, *J* = 12.6 Hz, CH), 129.5 (d, *J* = 2.8 Hz, CH), 128.7 (CH), 128.5 (CH), 128.2 (d, *J* = 8.5 Hz, C), 127.9 (d, *J* = 3.5 Hz, CH), 126.6 (CH), 125.4 (C), 117.5 (d, *J* = 85.8 Hz, C), 51.2 (CH_2), 30.9 (CH_2), 30.2 (CH_2), 14.6 (CH_3). **HRMS-ESI** Calculated for $C_{36}H_{33}N_3PS^+$ 570.2127 found 570.2129.

(4-(5-(Isopropylthio)-4-phenyl-1H-1,2,3-triazol-1-yl)butyl)triphenyl phosphonium bromide (3cd)

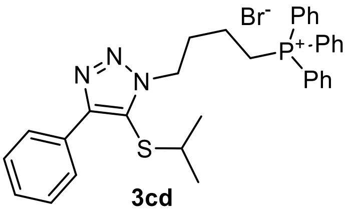

3cd

99% yield (carried out in a 9:1 Water: DMSO mixture). **Rf** = 0.58 in CH_2Cl_2:MeOH (90:10); flash column chromatography using CH_2Cl_2:MeOH (from 98:2 to 92:8) as eluent. **^{1}H NMR** (300 MHz, Chloroform-*d*) δ 88.04 (d, *J* = 7.2 Hz, 2H), 7.85 – 7.48 (m, 15H), 7.40-7.24 (m, 3H), 4.58 (t, *J* = 5.9 Hz, 2H), 3.91 (t, *J* = 14.5 Hz, 2H), 3.05 (hept, *J* = 6.2 Hz, 1H), 2.40-2.26 (m, 2H), 1.62-1.48 (m, 2H), 1.02 (d, *J* = 6.7 Hz, 6H). **^{13}C NMR** (75 MHz, Chloroform-*d*) δ 148.9 (C), 134.9 (d, *J* = 2.8 Hz, CH), 133.6 (d, *J* = 10.1 Hz, CH), 130.7 (C), 130.4 (d, *J* = 12.6 Hz, CH), 128.4 (CH), 128.3 (CH), 126.7 (CH), 125.1 (C), 117.9 (d, *J* = 86.0 Hz, C), 46.6 (CH_2), 40.9 (CH), 29.5 (d, *J* =17.0, CH_2), 22.9 (CH_3), 21.5 (d, *J* = 51.0 Hz, CH_2), 19.1 (d, *J* = 3.5 Hz, CH_2). **HRMS-ESI** Calculated for $C_{33}H_{35}N_3PS^+$ 536.2284 found 536.2284.

1-(4-Methylbenzyl)-5-(phenylthio)-4-(trimethylsilyl)-1H-1,2,3-triazole (3bb)

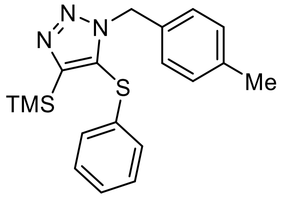

3bb

99% yield. **Rf** = 0.21 in Hexanes:EtOAc (80:20); flash column chromatography using Hexanes:EtOAc (80:20) as eluent. **^{1}H NMR** (300 MHz, Chloroform-*d*) δ 7.19 – 7.04 (m, 5H), 6.99 (d, *J* = 7.9 Hz, 2H), 6.84 – 6.71 (m, 2H), 5.46 (s, 2H), 2.26 (s, 3H), 0.30 (s, 9H). **^{13}C NMR** (75 MHz Chloroform-*d*) δ 153.1 (C), 138.0 (C), 134.8 (C), 131.7 (C), 131.5 (C), 129.3 (CH), 128.2 (CH), 126.3 (CH), 126.2 (CH), 51.5 (CH_2), 21.2 (CH_3), -1.20 (CH_3). **HRMS-ESI** Calculated for $C_{19}H_{24}N_3SSi^+$ 354.1455 found 354.1457.

(4-(5-(Benzylthio)-4-(3-phenylpropyl)-1H-1,2,3-triazol-1-yl)butyl)triphenyl phosphonium bromide (3cc)

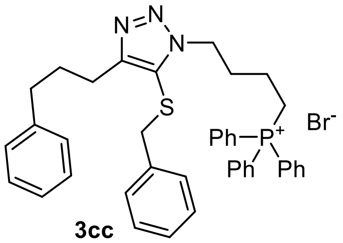

3cc

65% isolated yield. **Rf** = 0.26 in CH_2Cl_2:MeOH (90:10)**;** flash column chromatography using CH_2Cl_2:MeOH (from 98:2 to 92:8) as eluent.). **^{1}H NMR** (500 MHz, Chloroform-*d*) δ 7.72 (dd, *J* = 12.6, 7.4 Hz, 6H), 7.67 – 7.61 (m, 3H), 7.57 (td, *J* = 7.6, 3.4 Hz, 6H), 7.21 (t, *J* = 7.0 Hz, 2H), 7.14 – 7.07 (m, 6H), 6.83 (dd, *J* = 6.4, 2.9 Hz, 2H), 4.11 – 4.06 (m, 2H), 3.89 (tt, *J* = 13.5, 6.7 Hz, 2H), 3.65 (s, 2H), 2.53 (t, *J* = 7.7 Hz, 2H), 2.42 – 2.36 (m, 2H), 2.14 (dt, *J* = 13.1, 6.9 Hz, 2H), 1.81 (p, *J* = 7.8 Hz, 2H), 1.43 – 1.33 (m, 2H). **^{13}C NMR** (126 MHz, Chloroform-*d*) δ 152.5 (C), 142.0 (C), 136.6 (C), 135.0 (d, *J* = 2.8 Hz, CH), 133.7 (d, *J* = 10.0 Hz, CH), 130.4 (d, *J* = 12.6 Hz, CH), 128.8 (d, *J* = 9.6 Hz, CH), 128.5 (CH), 128.3 (CH), 127.8 (CH), 125.9 (CH), 124.8 (C), 118.2 (d, *J* = 85.9 Hz, C), 46.5 (CH_2), 40.9 (CH_2), 35.6 (CH_2), 30.7 (CH_2), 29.5 (d, *J* = 17.1 Hz, CH_2), 24.7 (CH_2), 21.5 (d, *J* = 50.9 Hz, CH_2), 19.3 (d, *J* = 3.6 Hz, CH_2). **HRMS-ESI** Calculated for $C_{40}H_{41}N_3PS,^+$ 626.2753 found 626.2756.

(S)-2-((((9H-Fluoren-9-yl)methoxy)carbonyl)amino)-6-(5-(ethylthio)-4-phenyl-1H-1,2,3-triazol-1-yl)hexanoic acid (3ea)

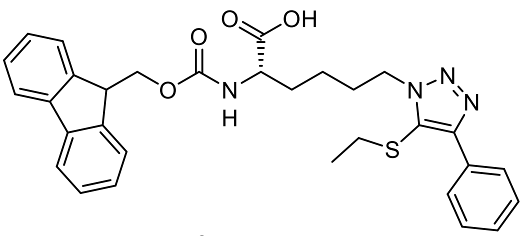

3ea

83% yield. **Rf** = 0.22 in CH_2Cl_2:MeOH (95:5); flash column chromatography using CH_2Cl_2:MeOH (from 100:0 to 90:10) as eluent **^{1}H NMR** (500 MHz, Chloroform-*d*) δ 8.04 (d, *J* = 7.4 Hz, 2H), 7.65 (d, *J* = 7.5 Hz, 2H), 7.54 – 7.41 (m, 2H), 7.35 (t, *J* = 7.5 Hz, 2H), 7.28 (t, *J* = 6.6 Hz, 3H), 7.20 (m 2H), 5.57 (d, *J* = 7.7 Hz, 1H), 4.44 – 4.23 (m, 5H), 4.12 (t, *J* = 6.9 Hz, 1H), 2.56 (q, *J* = 7.4 Hz, 2H), 2.04 – 1.81 (m, 2H), 1.75 (s, 1H), 1.18 (s, 1H), 0.99 (t, *J* = 7.4 Hz, 3H). **^{13}C NMR** (126 MHz, $CDCl_3$) δ 175.3 (C), 156.1 (C), 148.7 (C), 143.9 (C), 143.7 (C), 141.3 (C), 130.4 (C), 128.6 (CH) 128.6 (CH), 127.7 (CH), 127.1 (CH), 127.0 (CH), 125.2 (CH), 120.0 (CH), 67.1 (CH_2), 53.6 (CH), 47.9 (CH_2), 47.2 (CH), 31.8 (CH_2), 30.2 (CH_2), 29.7 (CH_2), 22.1 (CH_2), 14.67 (CH_3). **HRMS-ESI** Calculated for $C_{31}H_{33}N_4O_4S$ 557.2217 found 557.2216.

4-((5-(Ethylthio)-4-phenyl-1H-1,2,3-triazol-1-yl)methyl)-N,N-dimethylaniline (3fa)

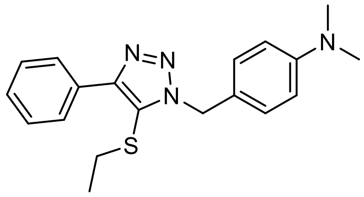

3fa

97% yield. **Rf** = 0.25 in Hexanes:EtOAc (80:20); flash column chromatography using hexanes:EtOAc (80:20) as eluent. **^{1}H NMR** (300 MHz, Chloroform-*d*) δ 8.20 (d, *J* = 7.4 Hz, 2H), 7.49 – 7.24 (m, 5H), 6.69 (d, *J* = 8.2 Hz, 2H), 5.60 (s, 2H), 2.94 (s, 6H), 2.47 (q, *J* = 7.2 Hz, 2H), 1.01 (t, *J* = 7.3 Hz, 3H). **^{13}C NMR** (75 MHz, Chloroform-*d*) δ 150.4 (C), 149.0 (C), 131.0 (C), 129.2 (CH), 128.5 (CH), 128.3 (CH), 126.9 (CH), 125.1 (C), 122.8 (C), 112.4 (CH), 51.8 (CH_2), 40.5 (CH_3), 30.1 (CH_2), 14.4 (CH_3). **HRMS-ESI** Calculated for $C_{19}H_{23}N_4S$ 339.1638 found339.1642

3.3. Orthogonality of the RuAtAC and CuAAC Annulations

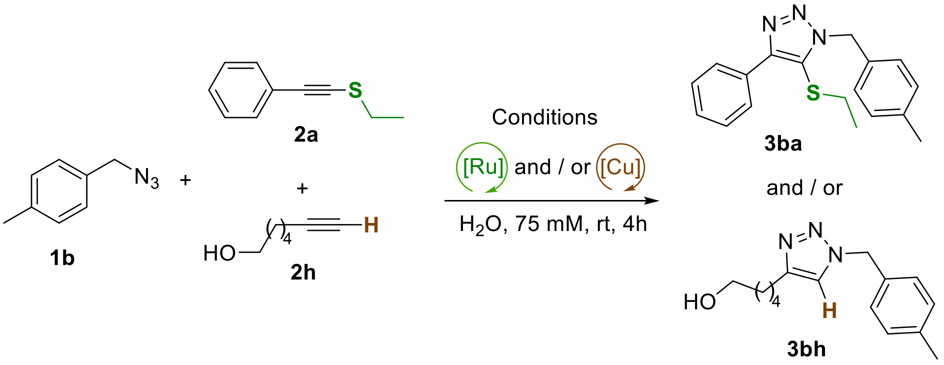

Selectivity of Ru2 towards the alkyne: treatment of a mixture of **1b, 2a** and **2h** with **Ru2**

In a 5 mL vial was added 50 mg of a solution of azide **1b** (5.5 mg, 37.5 μmol), thioalkyne **2a** (12.2 mg, 75 μmol) and alkyne **2h** (8.4 mg, 75 μmol) in DMSO (23.9 mg) followed by 400 μL of water and **Ru2** (1.1 mg, 1.9 μmol). The mixture was stirred for 2h, treated with a solution of EDTA-Na_2 (1 mL, 0.1M in water with 0.3 mL of aqueous ammonia/10 mL) for 5 min, extracted with CH_2Cl_2, filtered through a Florisil plug and dried under vacuo. Analysis by NMR, using 1,3,5-trimethozybenzene as internal standard confirmed the exclusive formation of **3ba** (79% yield), 0% yield of **3bh.**

Selectivity of the Cu-conditions towards the alkyne: treatment of a mixture of **1b, 2a** and **2h** with $CuSO_4{\cdot}5H_2O$ / Sodium ascorbate

In a 5 mL vial was added 50 mg of a solution of azide **1b** (5.5 mg, 37.5 μmol), thioalkyne **2a** (12.2 mg, 75 μmol) and alkyne **2h** (8.4 mg, 75 μmol) in DMSO (23.9 mg) followed by 400 μL of water, sodium ascorbate (25 μL, 150 mM in water) and $CuSO_4{\cdot}5H_2O$ (25 μL, 75 mM in water). The mixture was stirred for 2 h, treated with a solution of EDTA-Na_2 (1 mL, 0 .1 M in water with 0.3 mL of aqueous NH_3/10 mL) for 5 min, extracted with CH_2Cl_2, filtered through a Florisil plug and dried under vacuo. Analysis by NMR, using 1,3,5-trimethozybenzene as internal standard confirmed the exclusive formation of **3bh** (78% yield), 0% yield of **3ba.**

Sequential RuAtAC / CuAAC processes

In a 5 mL vial was added 50 mg of a solution of azide **1b** (5.5 mg, 37.5 µmol), thioalkyne **2a** (12.2 mg, 75 µmol) and alkyne **2h** (8.4 mg, 75 µmol) in DMSO (23.9 mg) followed by 400 µL of water and **Ru2** (1.1 mg, 1.9 µmol). The mixture was stirred for 2h followed by the addition of another equivalent of azide **1b** (5.5 mg, 37.5 µmol), sodium ascorbate (25 µL, 150 mM in water) and $CuSO_4 \cdot 5H_2O$ (25 µL, 75 mM in water). The mixture was stirred for another 2h, treated with a solution of EDTA-Na_2 (1 mL, 0.1M in water with 0.3 mL of aqueous ammonia/10 mL) for 5 min, extracted with CH_2Cl_2, filtered through a Florisil plug and dried under vacuo. Analysis by NMR, using 1,3,5-trimethozybenzene as internal standard confirmed the formation of both **3ba** (78% yield) and **3bh** (95% yield).

Sequential CuAAC / RuAtAC processes

In a 5 mL vial was added 50 mg of a solution of azide **1b** (5.5 mg, 37.5 µmol), thioalkyne **2a** (12.2 mg, 75 µmol) and alkyne **2h** (8.4 mg, 75 µmol) in DMSO (23.9 mg) followed by 400 µL of water, sodium ascorbate (25 µL, 150 mM in water) and $CuSO_4 \cdot 5H_2O$ (25 µL, 75 mM in water). The mixture was stirred for 2h followed by the addition of another equivalent of azide **1b** (5.5 mg, 37.5 µmol) and **Ru2** (1.1 mg, 1.9 µmol). The mixture was stirred for another 2h, treated with a solution of EDTA-Na_2 (1 mL, 0.1M in water with 0.3 mL of aqueous ammonia/10 mL), extracted with CH_2Cl_2 and filtered through a Florisil plug and dried under vacuo. Analysis by NMR, using 1,3,5-trimethozybenzene as internal standard confirmed the formation of both **3ba** (79% yield) and **3bh** (78% yield).

Simultaneous RuAtAC and CuAAC processes

In a 5 mL vial was added 50 mg of a solution of azide **1b** (5.5 mg, 37.5 µmol), thioalkyne **2a** (12.2 mg, 75 µmol) and alkyne **2h** (8.4 mg, 75 µmol) in DMSO (23.9 mg) followed by 425 µL of water, sodium ascorbate (25 µL, 150 mM in water), **Ru2** (1.1 mg, 1.9 µmol) and $CuSO_4 \cdot 5H_2O$ (0.5 mg, 1.9 µmol). The mixture was stirred for 2h, treated with a solution of EDTA-Na_2 (1 mL, 0.1M in water with 0.3 mL of aqueous ammonia/10 mL), for 5 min, extracted with CH_2Cl_2, filtered through a Florisil plug and dried under vacuo. Analysis by NMR, using 1,3,5-trimethozybenzene as internal standard confirmed the formation of both **3ba** (44% yield) and **3bh** (50% yield).

5-(1-(4-Methylbenzyl)-1H-1,2,3-triazol-4-yl)pentan-1-ol. White solid. **Rf** = 0.17 in Hexanes:EtOAc (70:30).

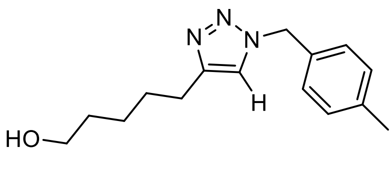

3bh

Flash column chromatography using Hexanes:EtOAc (70:30) as eluent. **^{1}H NMR** (300 MHz, Chloroform-*d*) δ 7.32 – 7.03 (m, 5H), 5.42 (s, 2H), 3.61 (t, *J* = 6.5 Hz, 2H), 2.74 (bs, 1H), 2.67 (t, *J* = 7.6 Hz, 2H), 2.33 (s, 3H), 1.84 – 1.52 (m, 4H), 1.48 – 1.27 (m, 2H). **^{13}C NMR** (75 MHz, Chloroform-*d*) δ 148.5 (C), 138.5 (C), 131.9 (C), 129.7 (CH), 128.1 (CH), 120.6 (CH), 62.5 (CH_2), 53.8 (CH_2), 32.4 (CH_2), 29.1 (CH_2), 25.6 (CH_2), 25.4 (CH_2), 21.2 (CH_3). **HRMS-ESI** Calculated for $C_{15}H_{22}N_3O$ 260.1757 found 260.1753

3.4 RuAtAC promoted by Cp*Ru(II) sandwich complexes in water (exemplified for the cycloaddition of **1b** and **2a** with **Ru4,** with irradiation)

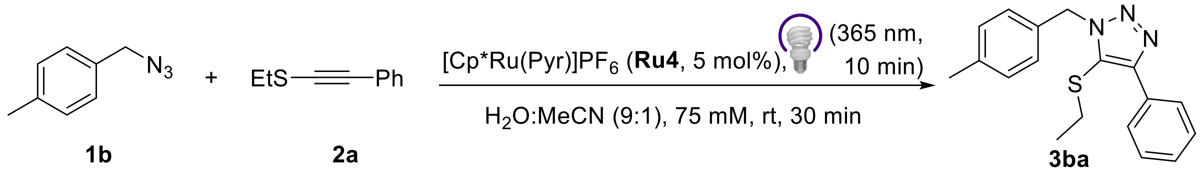

Ru4 (1.9 mg, 3.8 μmol), thioalkyne **2a** (24.3 mg, 150 μmol), H_2O:MeCN (9:1, 1 mL), and azide **1b** (11.0 mg, 75 μmol) were sequentially added to a dry vial under air to a vial. The mixture was irradiated at 365 nm for 10 min and stirred for 0.5 h. Then, the reaction mixture was extracted with CH_2Cl_2, filtered through a plug of Florisil, concentrated, and analysed by NMR using trimethoxybenzene as internal standard. The resulting product, **3ab** was formed in 99% yield.

Table S1. **Optimization of the RuAtAC reaction using [Cp*Ru(arene)]X precatalysts.**

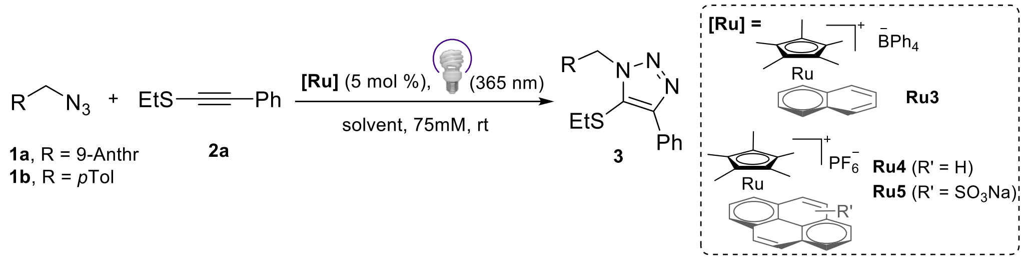

Entry	Azide (**1**)	**[Ru]**	Time (h)	Solvent	hv (365nm) time	Conv (%)	yield (%)
1	**1a**	**Ru4**	2	H_2O	–	7	0
2	**1a**	**Ru4**	2	CH_2Cl_2	–	10	0
3	**1a**	**Ru4**	0.5	MeCN	–	18	0
4	**1b**	**Ru4**	0.5	MeCN	–	10	7
5	**1b**	**Ru4**	0.5	H_2O	–	5	0
7	**1a**	**Ru4**	1	H_2O:Acetone (1:1)	30 min	70	28
8	**1a**	**Ru4**	2	H_2O:Acetone (1:1)	–	10	0
12	**1b**	**Ru4**	2	H_2O	10 min	7	0
13	**1b**	**Ru4**	2	H_2O:MeCN (1:1)	10 min	99	99
14	**1b**	**Ru4**	2	H_2O:MeCN (1:1)	–	30	24
15	**1b**	**Ru4**	2	H_2O:MeCN (1:1)	– [b]	35	5
16	**1b**	**Ru4**	2	H_2O:MeOH (1:1)	10 min	53	49
17	**1b**	**Ru4**	2	H_2O:MeOH (1:1)	-	5	0
18	**1b**	**Ru4**	2	H_2O:MeOH (1:1)	20 min	77	66
21	**1b**	–	2	H_2O:MeCN (1:1)	10 min	10	0
22	**1b**	**Ru4**	2	H_2O:MeCN (9:1)	10 min	99	99
23	**1b**	**Ru4**	2	H_2O:MeCN (9:1)	– [b]	1	0
24	**1b**	**Ru3**	2	H_2O:MeCN (9:1)	10 min	90	90
25	**1b**	**Ru3**	2	H_2O:MeCN (9:1)	– [b]	3	3
26	**1b**	**Ru5**	2	H_2O:MeCN (9:1)	10 min	80	70
27	**1b**	**Ru5**	2	H_2O:MeCN (9:1)	– [b]	0	0

[a] Azide **1** was added after irradiation (anthracenyl azide **1a** does not stand irradiation at 365 nm). [b] Reaction vial was fully covered with aluminium foil to avoid any kind of light exposure.

4. General Procedure for the RuAtAC under Micromolar Conditions

RuAtAC promoted by Ru2 in water (exemplified for the cycloaddition of **1c** and **2a** at 500 µM)

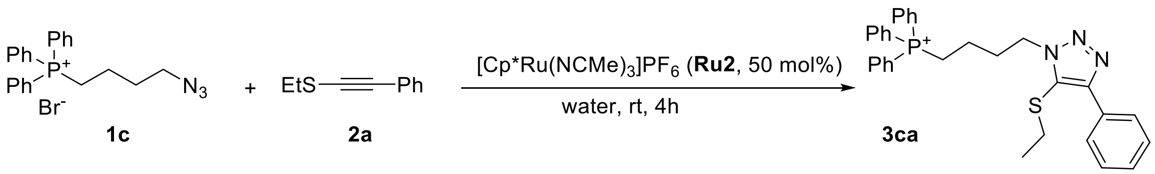

Thioalkyne **2a** (5 µL, from a stock solution 100 mM in DMSO, 2.0 eq.), azide **1c** (5 µL, from a stock solution 50 mM in DMSO, 1.0 eq.), water (500 µL) and **Ru2** (5 µL, from a stock solution 25 mM in DMSO, 0.5 eq.) were sequentially added to a HPLC vial equipped with a magnetic stir bar. The mixture was stirred for 4 h, diluted with MeOH (500 µL). 200 µL of the resulting solution were taken and diluted again with methanol (300 µL) to afford a 100 µM theoretical concentration of the expected triazole product **3ca**. Coumarin (IS, 2.5 µL of a stock solution 20Mm in DMSO) was added as internal standard (final concentration of 100 µM) and the mixture analyzed by HPLC-MS, which allowed to determine a 99% yield of **3ac**.

RuAtAC promoted by ruthenium (II) sandwich complexes in water (exemplified for the cycloaddition of **1c** and **2a** with **Ru4**)

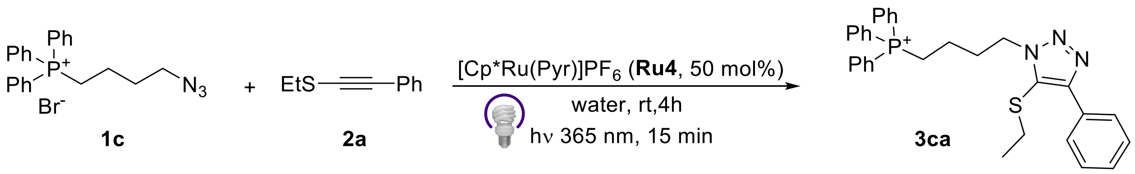

Thioalkyne **2a** (5 µL from a stock solution 100 mM in DMSO, 2.0 eq.), azide **1c** (5 µL, from a stock solution 50 mM in DMSO, 1.0 eq.), water (500 µL) and **Ru4** (5 µL, from a stock solution 25 mM in DMSO, 0.5 eq.) were sequentially added to a HPLC vial equipped with a magnetic stir bar. The mixture was irradiated for 15 min, stirred for 4 h, diluted with MeOH (500 µL). 200 µL of the resulting solution were taken and diluted again with methanol (300 µL) to afford a 100 µM theoretical concentration of the expected triazole product **3ca**. Coumarin (IS, 2.5 µL of a stock solution 20 mM in DMSO) was added as internal standard (final concentration of 100 µM), and the mixture analyzed by HPLC-MS, which allowed to determine a 99% yield of **3ac** (see Figure S3).

a)

b)

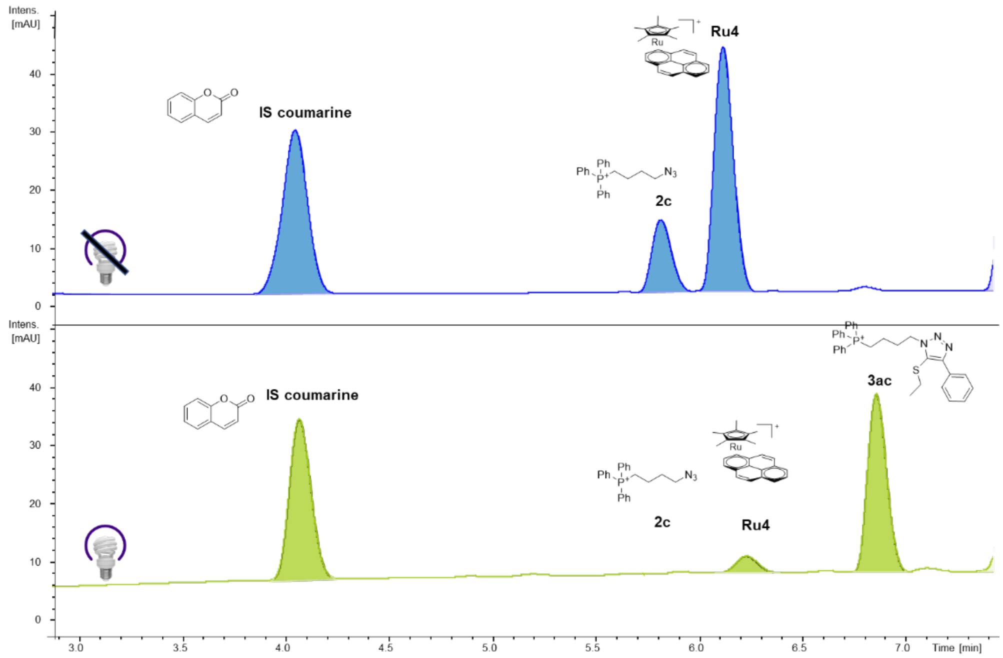

Figure S3. Reaction between thioalkyne 2a, azide 1a, promoted by Ru4 /hν. Yield by UHPLC-MS, using coumarine as internal standard (IS). a) Reaction control using **Ru4** without irradiation. b) Reaction using **Ru4** with 15 min irradiation at 365 nm.

Calibration curves for yield determinations by HPLC

HPLC calibration curves were made by addition of a fixed quantity of and internal standard (IS, coumarin, final concentration 100 μM) to the stock solutions of the corresponding compound. Curves are the result of the division of the area of the corresponding compound (S) against the internal standard (IS, absorption area recorded at 270 nm) plotted against the concentration of the sample.

A) HPLC calibration curve for triazole 3ca

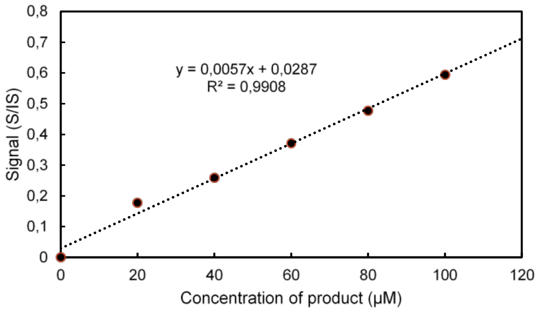

C) HPLC calibration curve triazole 3da

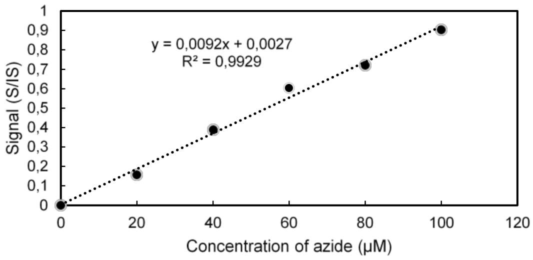

B) HPLC calibration curve for azide 1c

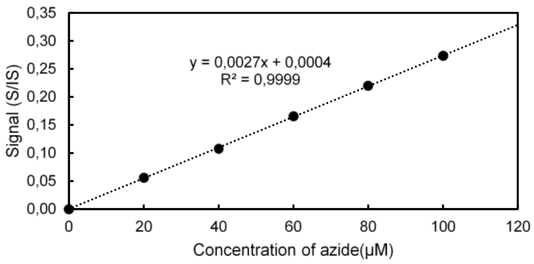

Figure S4. Calibration curves for yield determinations by HPLC.

5. Performance of Ru1- Ru4 in the Reaction of 1d and 2a at Micromolar Conditions

Besides the comparison shown in Figure 2 of the main manuscript, between **1c** and **2a**, we also analyzed the behaviour of the different ruthenium precatalysts in the RuAtAC between **1d** and **2a**. The reactions were conducted in HPLC vials, using stock solution of the reagents and the ruthenium complexes. The reaction yields were determined by UHPLC-MS using coumarin (100 µM) as internal standard. Results are the average of three different reactions. Reaction mixtures in presence of **Ru4** and **Ru5** were irradiated for 15 min at 365 nm to activate de catalyst. Controls without irradiation for **Ru4** and **Ru5** provided yields <1%

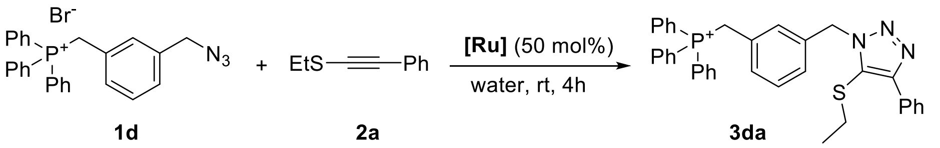

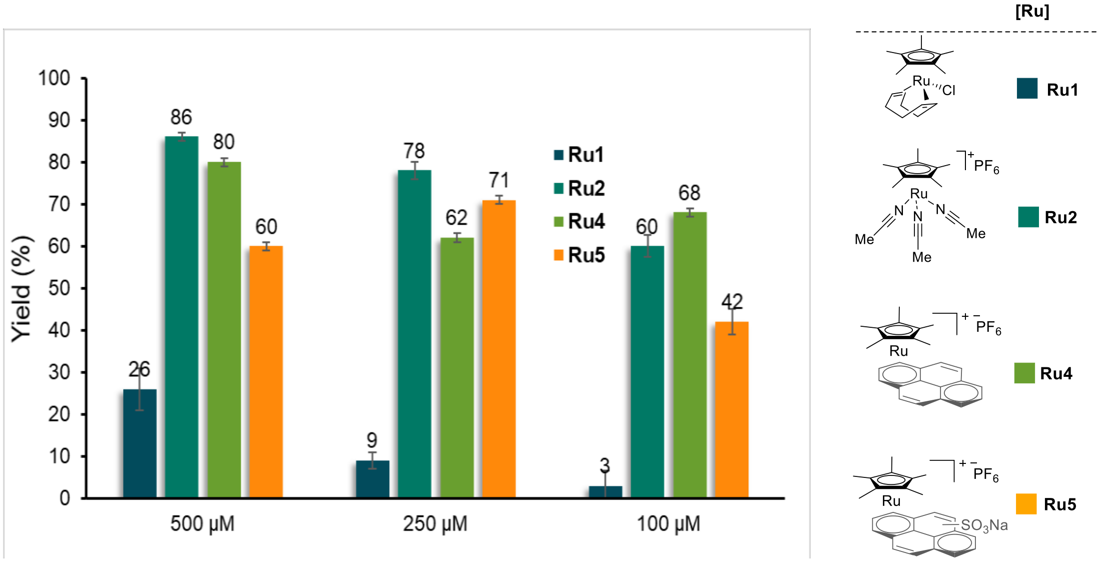

Figure S5. Comparison of the catalyst performances at the micromolar range.

6. Influence of the Catalyst Loading (Ru2) at Different Micromolar Concentrations

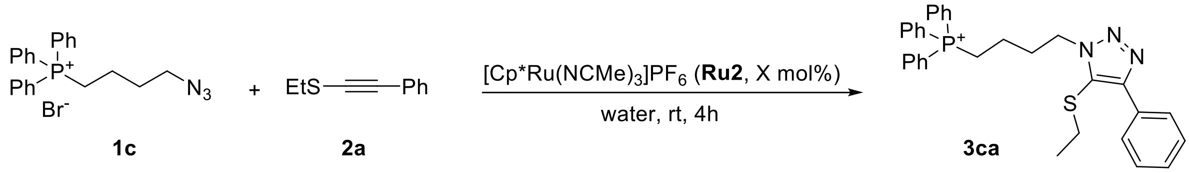

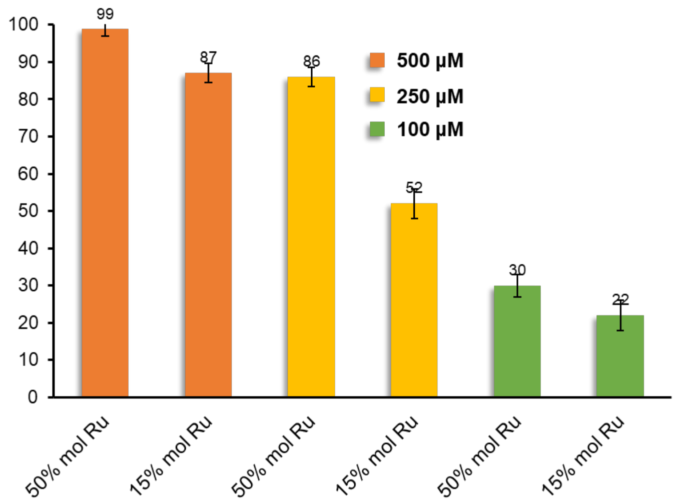

Figure S6. Performance of Ru2 in the micromolar range with different ruthenium loadings. Reactions were conducted in HPLC vials, using stock solution of the reagents and **Ru2**. Yields determined by UHPLC-MS using coumarin as internal standard. Results are the average of three different reactions.

7. Influence of the Catalyst Loading (Ru4) at Different Micromolar Concentrations

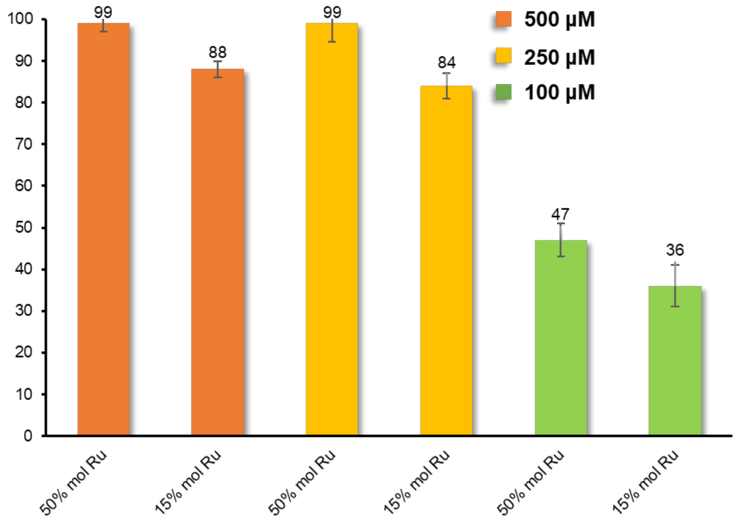

Figure S7. Performance of Ru4 in the micromolar range with different ruthenium loadings. Reactions were conducted in HPLC vials, using stock solution of the reagents and **Ru4.** Reactions were irradiated at 365 nm for 15 min and stirred for 4h. Yields determined by UHPLC-MS using coumarin as internal standard. Results are the average of three different reactions.

8. Assessment of the Stability of Ru4 in Biologically Relevant Media

Ruthenium complex **Ru4** (2.5 µL from a stock solution 10 mM in DMSO) was added to a vial containing 500 µL of the corresponding reaction media (HeLa Cell lysates or DMEM), and the mixture was stirred for 24 h at rt. After the indicated time the solution was diluted with MeOH and analyzed by HPLC MS.

In neither case decomposition of the catalyst was observed.

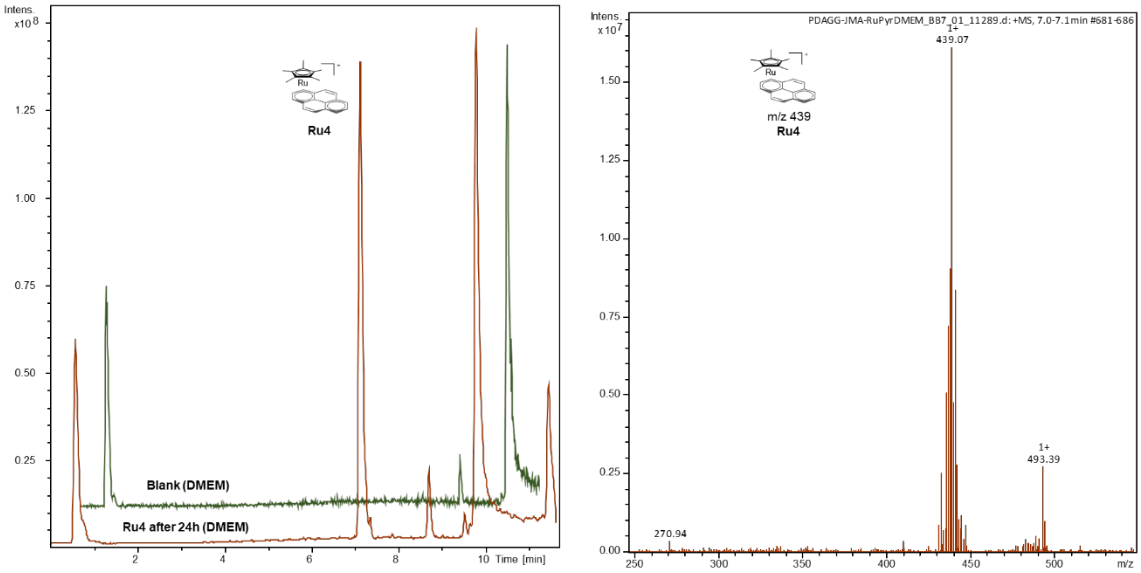

Figure S8. a) MS-chromatogram and MS-spectra for the stability of the complex **Ru4** after 24 h in DMEM (Dulbecco´s modified Eagle´s medium)

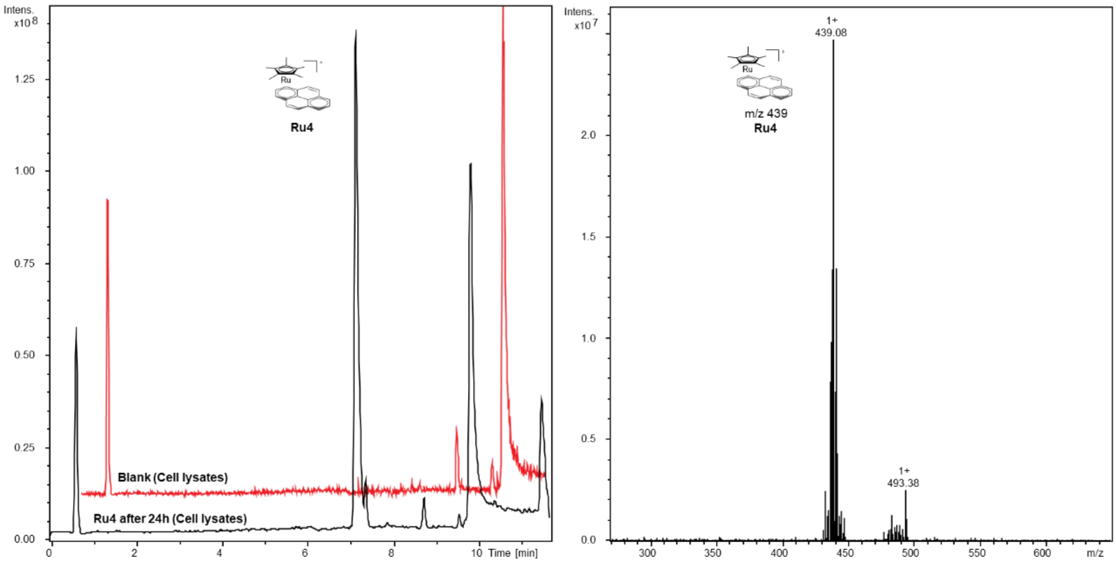

Figure S9. a) MS-chromatogram and MS-spectra for the stability of the complex **Ru4** after 24 h in HeLa Cell Lysates (5 mg/ mL)

9. Comparative of Ir, Rh and Ru complexes, under micromolar conditions

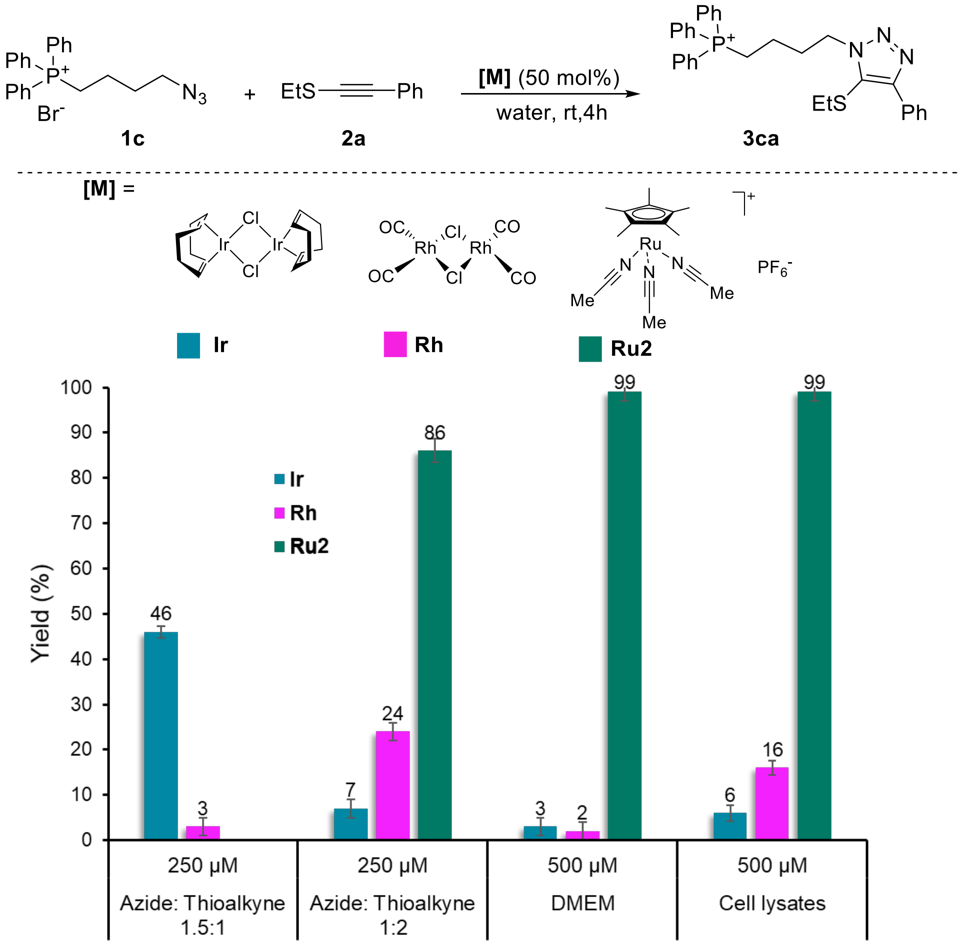

Figure S10. Comparative of Ir, Rh and Ru complexes, in the micromolar range. Reactions were conducted in HPLC vials, using stock solutions of the reagents and the corresponding metal complex. Yields determined by UHPLC-MS, using coumarin as internal standard. Results are the average of three different runs. Due to the dimeric nature of the Rh and Ir complexes, 25 mol% of the complexes were used. [**Note**: We first analyzed different azide:thioalkyne ratios in water (250 μM), to select the optimal conditions for each metal catalyst (1.5:1 ratio for Ir) and (1:2 ratio for Rh); In the subsequent experiments in DMEM and HeLa cell lysates, these optimal azide:thioalkyne ratios were used].

9 MS- Speciation Experiments

Speciation experiments were performed by dissolving the [Cp*Ru(MeCN)$_3$]PF$_6$ (**Ru2**) in the corresponding solvent and injecting the sample directly into either a MS-ESI Bruker Solarix XR or a Bruker AmaZon SL

A) [Cp*Ru(NCMe)$_3$]PF$_6$ speciation in MeCN

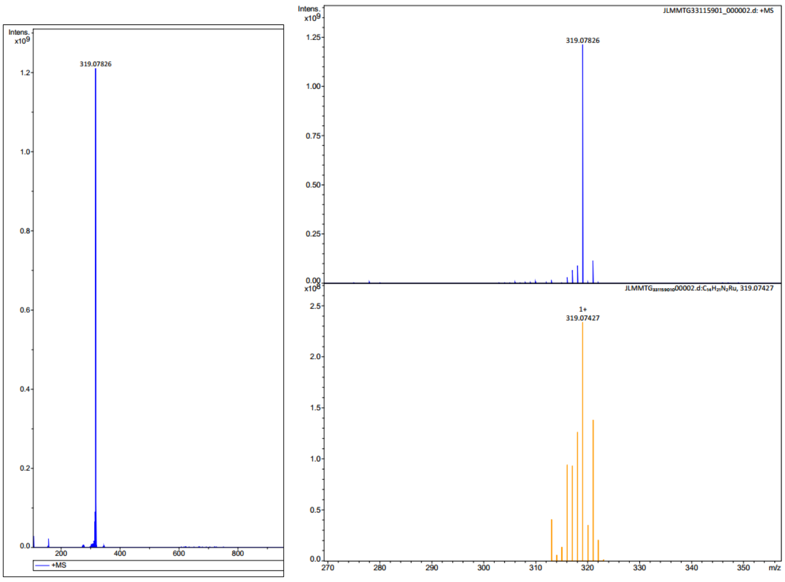

[Cp*Ru(MeCN)$_2$] m/z =319; **HRMS-ESI** Calculated for $C_{14}H_{21}N_2Ru$ 319.07427 found 319.07826;

B) [Cp*Ru(MeCN)$_3$]PF$_6$ speciation in MeCN:Water (2:8)

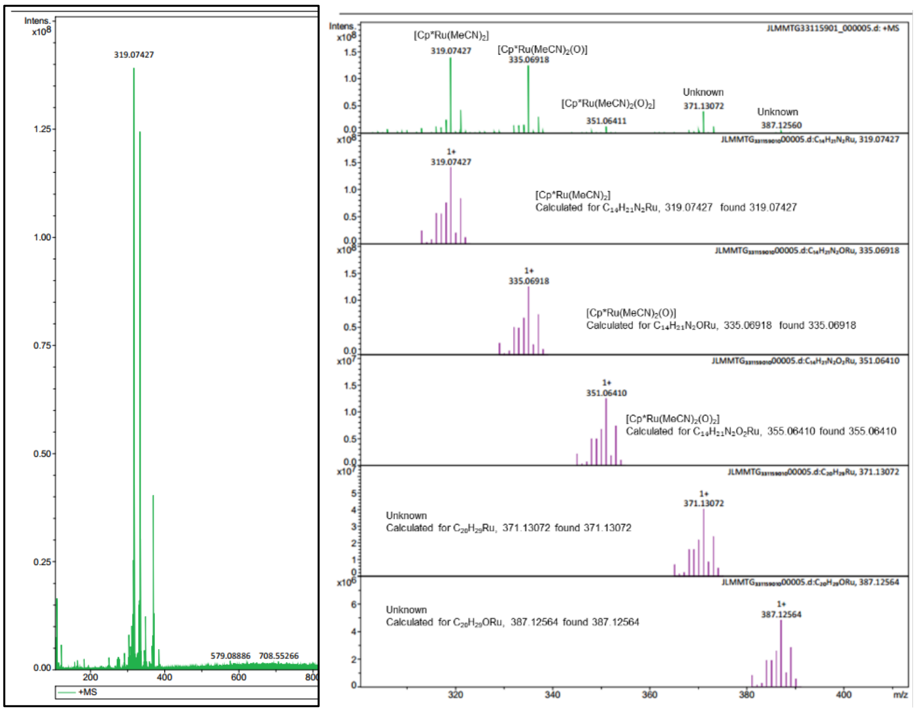

[Cp*Ru(MeCN)$_2$] m/z = 319; **HRMS-ESI** Calculated for $C_{14}H_{21}N_2Ru$ 319.07427 found 319.07827; [Cp*Ru(MeCN)$_2$(O)] m/z = 335; **HRMS-ESI** Calculated for $C_{14}H_{21}N_2ORu$ 335.06918 found 335.06918; [Cp*Ru(MeCN)$_2$(O)$_2$] m/z = 351; **HRMS-ESI** Calculated $C_{14}H_{21}N_2ORu$ 351.06140 found 351.06140.

C)[Cp*Ru(MeCN)$_3$]PF$_6$ speciation in water (Bruker AmaZon SL)

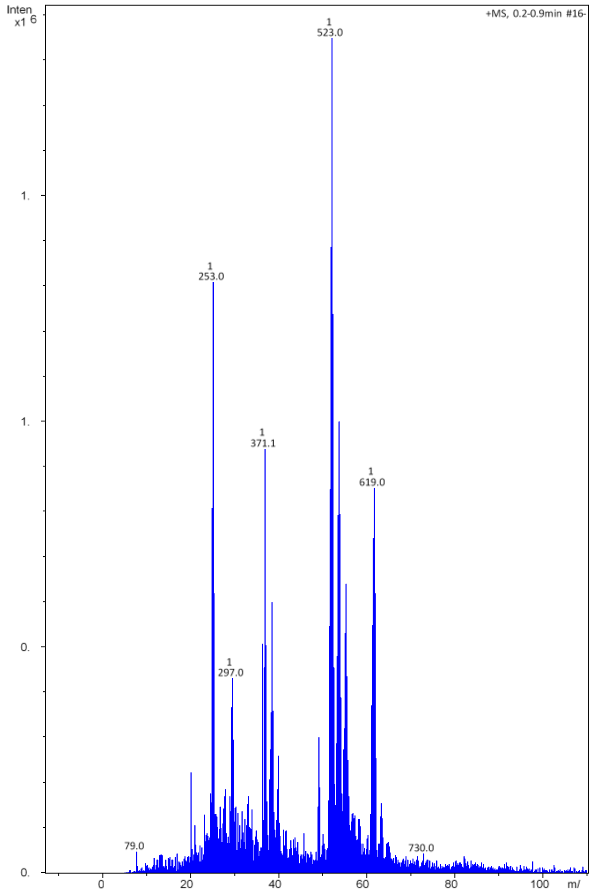

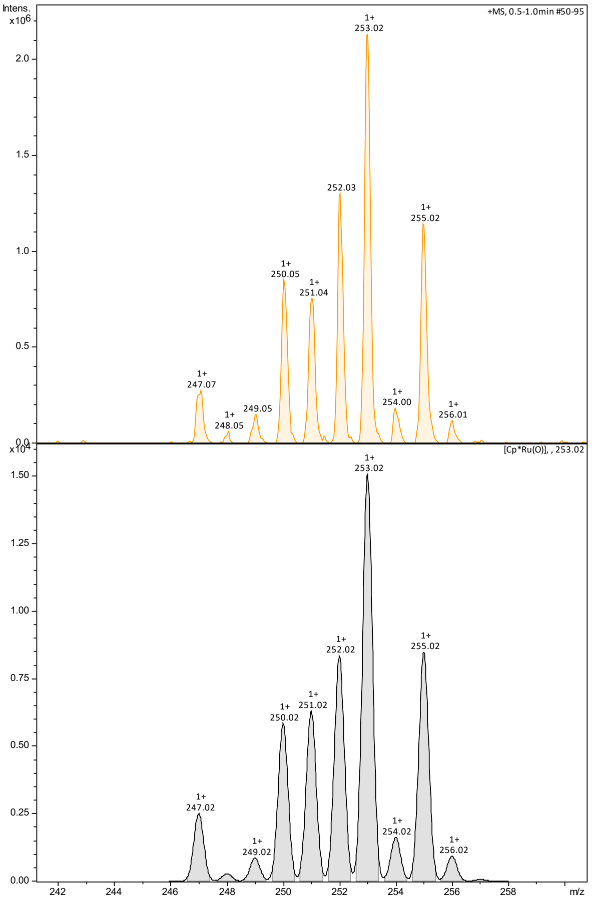

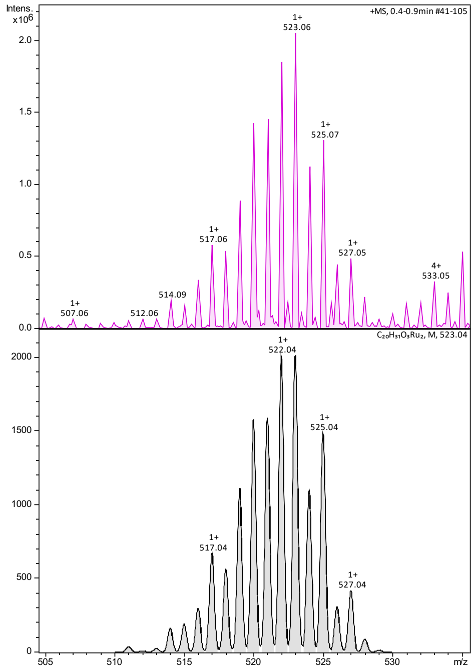

[Cp*Ru(O)]$^+$ m/z = 253; **MS-ESI** Calculated for $C_{10}H_{15}ORu$ 253.02 found 253.02

[Cp*Ru(O)]$_2$(OH)]$^+$ m/z = 523; **MS-ESI** Calculated for $C_{20}H_{31}O_3Ru_2$ 523.04 found 523.06

Figure S11. MS- Speciation of [Cp*Ru(NCMe)$_3$]PF$_6$ in a) MeCN; b) MeCN:Water (2:8); c) water.

10 Labelling of Biomolecules

Oligonucleotides

HPLC assays for DNA quantification were performed in an *Agilent 1100* Series HPLC System using a Phenomenex Luna-C18(2) column 100 Å (250 x 4.6 mm, 5 μM), 1 mL/min, gradient 6 min isocratic at 0% B and then 0-73% over 40 min. (A: 95:5 H_2O:ACN containing 100mM TEAA pH 7.0, B: 70:30 ACN:H_2O containing 100 mM TEAA pH 7.0). Determination of the reaction yield was achieved by integration of the peaks recorded at 260 nm, with correction for the contribution of the rhodamine dye at this wavelength. Triethylammonium acetate buffer (TEAA pH 7.0, 1 M) was prepared from acetic acid and triethylamine following standard procedures.

EMSA experiments were performed using a *BioRad Mini Protean* gel system powered by an electrophoresis power supplier *PowerPac Basic* model at a constant voltage of 120 V.

The products were resolved by PAGE using a 15% non-denaturing polyacrylamide gel and 0.5X TBE buffer for 40 min at 20ºC. Gels were visualized by fluorescence, first after illumination of the rhodamine-labelled band and then by staining with SybrGold (Molecular Probes: 5 µL in 50 mL in 0.5X TBE) for 10 min to visualize the remaining unlabelled ssDNA.

Oligonucleotide modification with **Ru2:** In a HPLC vial the Azide-ssDNA (40 nmol, 1.0 eq.) was diluted with TEAA buffer (pH = 7, 0.1 M) up to a final volume of 200 µL, then the thioalkyne **2i** (80 nmol, 2 eq.), and finally the precatalyst **Ru2** (20 nmol, 0.5 eq.) were added and the mixture was stirred overnight, diluted to 100 µM and analysed by HPLC.

Oligonucleotide modification with **Ru4:** In a HPLC vial the Azide-ssDNA (40 nmol) was diluted with TEAA buffer (pH = 7, 0.1 M) up to a final volume of 200 µL, then the thioalkyne **2i** (80 nmol), and **Ru4** (20 nmol) were added, the reaction mixture was irradiated for 15 min and stirred overnight, diluted to 100 µM and analysed by HPLC.

NOTE: We did not observe any reactivity in the absence of the Ru catalysts while, in the presence of the ruthenium catalysts, we did not detect (by HPLC) any other type of product that could be indicative of side processes.

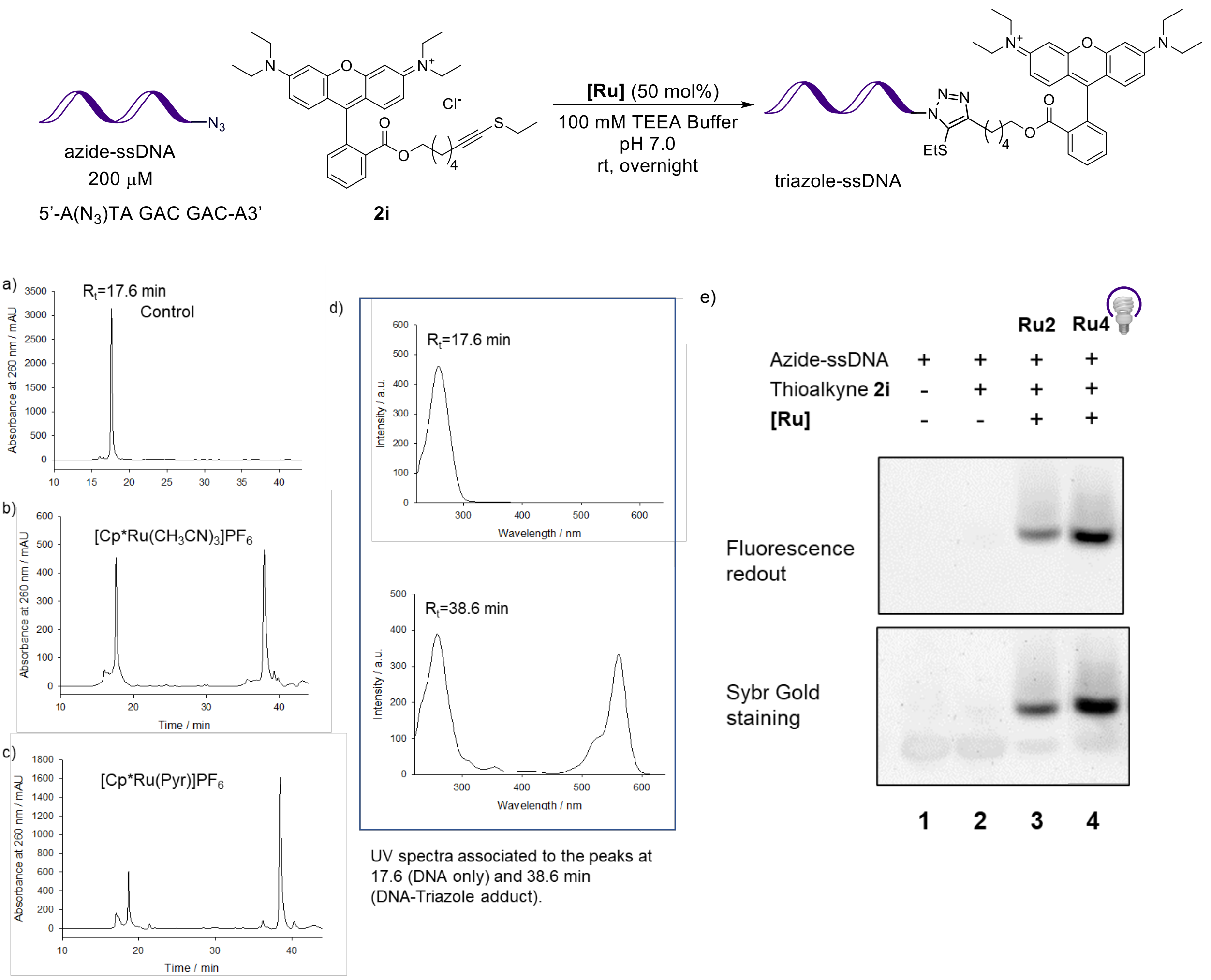

Figure S12. A) Labelling of Azide-ssDNA with 2i a) Control chromatogram: Azide-ssDNA without **[Ru]** b) Chromatogram of the reaction promoted by **Ru2** c) Chromatogram of the reaction promoted by **Ru4** /(+hν); d) Absorption spectra of the Azide-ssDNA (Rt = 17.6 min) and triazole-ssDNA (Rt = 38.6 min). e) Comparative EMSA for the labelling with **Ru2** and **Ru4** (+hν). 100 pmol DNA on each well.

Peptides

Reagents and amino acid derivatives for peptide synthesis were purchased from Sigma Aldrich and Iris Biotech; amino acids were purchased as protected Fmoc amino acids with the standard side chain protecting scheme: Fmoc-Lys(N_3)-OH, Fmoc-Phe-OH, Fmoc-Ile-OH, FmocTyr-OH, Fmoc-Pro-OH Fmoc-His(Trt)-OH and Fmoc-Val-OH. All solvents were dry and synthesis grade, unless specifically noted. Peptides were synthesized using an automatic peptide synthesizer CEM Liberty Lite, following the recommended procedures by the manufacturer: Peptide syntheses was performed using Fmoc strategy on a Rink-amide-ChemMatrix (0.5 mmol/g) using DIC as activator, oxyma (ethyl(hydroxyimino)cyanoacetate) as base, and DMF as solvent. The removal of the Fmoc protecting group was performed by treating the resin with 20% piperidine in DMF. Cleavage/deprotection step was performed by treatment of the resin-bound peptide for 2h with the following cleavage cocktail: 900 µL TFA, 50 µL CH_2Cl_2, 25 µL H_2O and 25 µL triisopropylsilane (1 mL of cocktail / 40 mg resin). The resin was filtered, and the cocktail was added onto ice-cold Et_2O. After 10 - 30 min, the precipitate

was centrifuged and washed again with of ice-cold ether. The solid residue was dried under nitrogen and re-dissolved in water. The synthesized peptides were analysed by analytical UHPLC-MS with an Agilent 1200 series LC/MS using a SB C18 (1.8 µm, 2.1 × 50 mm) analytical column from Phenomenex. Standard conditions for analytical UHPLC consisted on a linear gradient from 5 to 95% of solvent B for 20 min at a flow rate of 0.35 mL/min (A: water with 0.1% TFA, B: acetonitrile with 0.1% TFA). Compounds were detected by UV absorption at 222, 270, and 330 nm. Electrospray Ionization Mass Spectrometry (ESI/MS) was performed with an Agilent 6120 Quadrupole LC/MS model in positive scan mode using direct injection of the purified peptide solution into the MS detector.

Procedure for the RuAtAC for peptide labelling

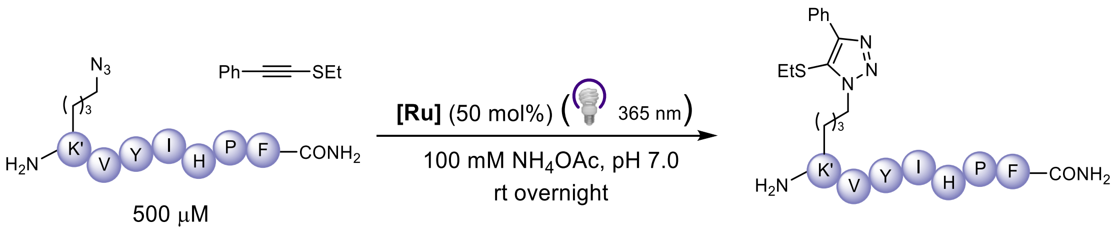

Procedure using **Ru2:** In a HPLC vial, the peptide NH_2-K(N_3)-V-Y-I-H-P-F-$CONH_2$ (250 nmol) was diluted in a NH_4OAc solution (0.1 M) up to a final volume of 500 µL. Then, thioalkyne **2a** (5 µL, 500 nmol from a stock solution 100 mM in DMSO)), and **Ru2** (5 µL, 125 nmol from a stock solution 25 mM in DMSO) were added, and the mixture was stirred overnight. Then, the sample was diluted to 100 µM and the coumarine internal standard was added (100 µM). Determination of the reaction yield of the triazole-peptide (55% yield) was achieved by analysing the conversion of the azide-peptide (N_3-K-V-Y-I-H-P-F-$CONH_2$) into the product, determining the ratio of the areas recorded at 220 nm. The yields are the average of three reactions.

Procedure using **Ru4 / hν**: In a HPLC vial, the peptide NH_2-K(N_3)-V-Y-I-H-P-F-$CONH_2$ (250 nmol) was diluted in a NH_4OAc solution (0.1 M) up to a final volume of 500 µL Then, thioalkyne **2a** (5 µL, 500 nmol from a stock solution 100 mM in DMSO)), and **Ru4** (5 µL, 125 nmol from a stock solution 25 mM in DMSO) were added, the reaction mixture was irradiated for 15 min (365 nm) and stirred overnight. Then, the sample was diluted to 100 µM, and the coumarin internal standard was added (100 µM). Analysis by HPLC determined a 84% yield of the triazole-peptide.

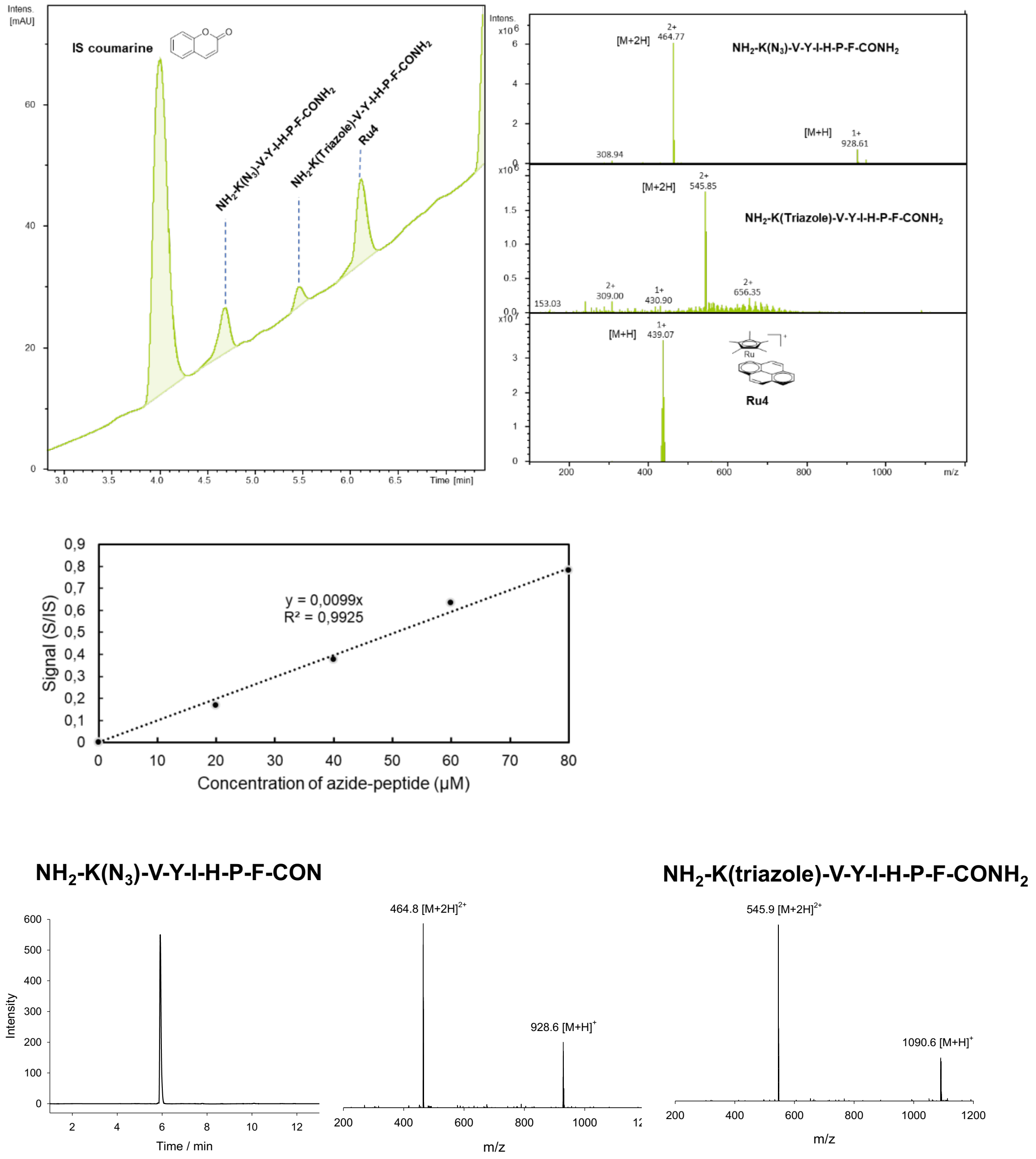

MS profile of NH_2-K(N_3)-V-Y-I-H-P-F-$CONH_2$ Calculated mass for $C_{46}H_{65}N_{13}O_8$: 927.5. Found: 928.6 $[M+H]^+$; 464.8 $[M+2H]^{2+}$; MS profile NH_2-K(triazole)-V-Y-I-H-P-F-$CONH_2$. Calculated mass for $C_{56}H_{75}N_{13}O_8S$: 1089.6. Found: 1090.6 $[M+H]^+$; 545.9 $[M+2H]^{2+}$.

Figure S13. RuAtAC for peptide labelling.

11. Experiments in the Presence of Cells

Cell Culture: HeLa cells were cultured in DMEM (Dulbecco´s modified Eagle´s medium), 5 mM glutamine, penicillin (100 units/mL) and streptomycin (100 units/mL) (all from Invitrogen). Proliferating cultures were maintained in a 5% CO_2 humidified incubator at 37 °C. For all the experiments, cells were suspended in DMEM-HEPES without Phenol Red) at 10^6 cells/mL.

RuAtAC between 1c and 2a promoted by Ru2, in the presence of cells

500 µL of a HeLa cell suspension (10^6 cells / mL) in DMEM-HEPES (without phenol-red) were transferred to a HPLC vial followed by sequential addition of the thioalkyne **2a** (10 µL, from a stock solution 40 mM in DMSO, 8.0 eq.), azide **1c** (2.5 µL, from a stock solution 20 mM in DMSO, 1.0 eq.) and **Ru2** (2.5 µL, from a stock solution 10 mM in DMSO, 0.5 eq.).The resulting suspension was kept at 37 ºC and stirred for 2 h at 80 rpm. After the indicated time 400 µL of the suspension were taken to Eppendorf vial and centrifuged at 10 000 rpm for 4 min. The supernatant was transferred to a HPLC vial diluted with 400 µL of MeOH ($80\%_{v/v}$), coumarin was added as IS (4 µL of a stock solution 20 mM in DMSO) and analyzed by HPLC-MS. The remaining cell pellet was treated with 200 µL of MeOH (80% $_{v/v}$) and shaked at 1000 rpm for 5 min, followed by centrifugation at 10 000 rpm for 5 min. The resulting extract was transferred to a HPLC vial diluted with 200 µL of MeOH (80% $_{v/v}$), coumarin was added as IS (2 µL of a stock solution 20 mM in DMSO) and analyzed by HPLC-MS.

a)

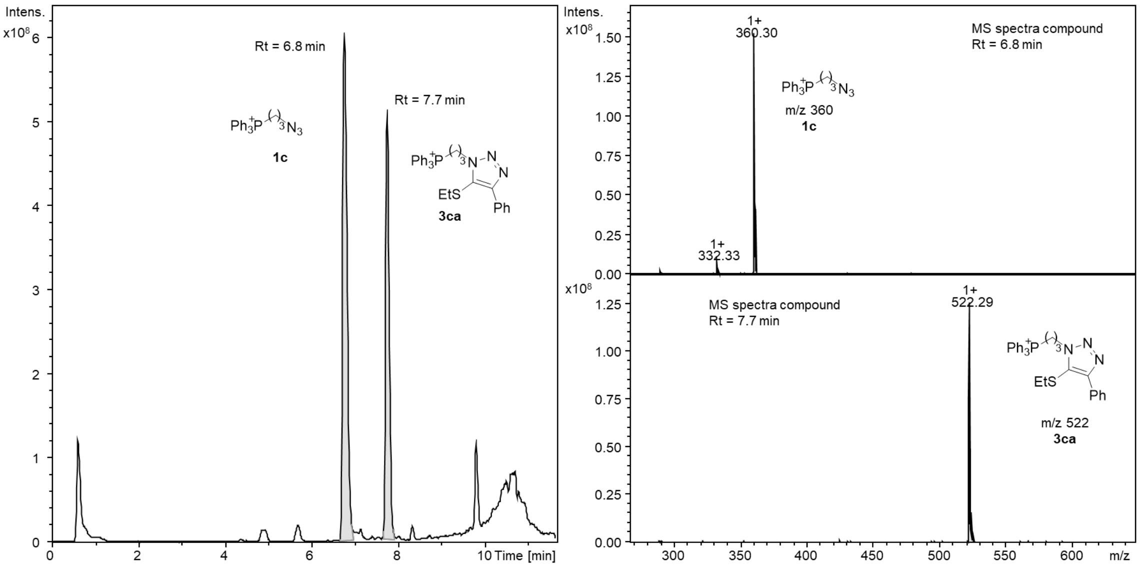

b)

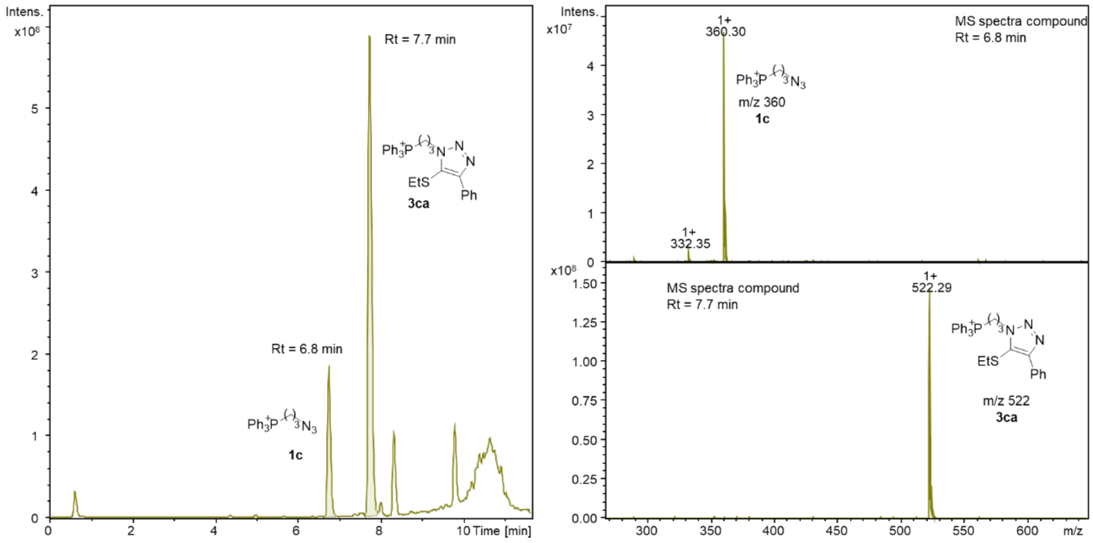

Figure S14. a) MS-chromatogram and MS-spectra for the indicated peaks in the supernatant. b) MS-chromatogram and MS-spectra for the indicated peaks in the methanolic extract.

RuAtAC between 1c and 2a promoted by Ru4, in the presence of cells

500 µL of a HeLa cell suspension (10^6 cells / mL) in DMEM-HEPES (without phenol-red) were transferred to a HPLC vial followed by sequential addition of the thioalkyne **2a** (10 µL, from a stock solution 40 mM in DMSO, 8.0 eq.), azide **1c** (2.5 µL, from a stock solution 20 mM in DMSO, 1.0 eq.) and **Ru4** (2.5 µL, from a stock solution 10 mM in DMSO, 0.5 eq.).The resulting suspension was irradiated at 365 nm for 10 min, then kept at 37 ºC and stirred for 2 h at 80 rpm. After the indicated time 400 µL of the suspension were taken to Eppendorf vial and centrifuged at 10 000 rpm for 4 min. The supernatant was transferred to a HPLC vial diluted with 400 µL of MeOH (80%$_{v/v}$), coumarin was added as IS (4 µL of a stock solution 20 mM in DMSO) and analyzed by HPLC-MS. The remaining cell pellet was treated with 200 µL of MeOH (80% $_{v/v}$) and shaked at 1000 rpm for 5 min, followed by centrifugation at 10 000 rpm for 5 min. The resulting extract was transferred to a HPLC vial diluted with 200 µL of MeOH (80% $_{v/v}$), coumarin was added as IS (2 µL of a stock solution 20 mM in DMSO) and analyzed by HPLC-MS.

a)

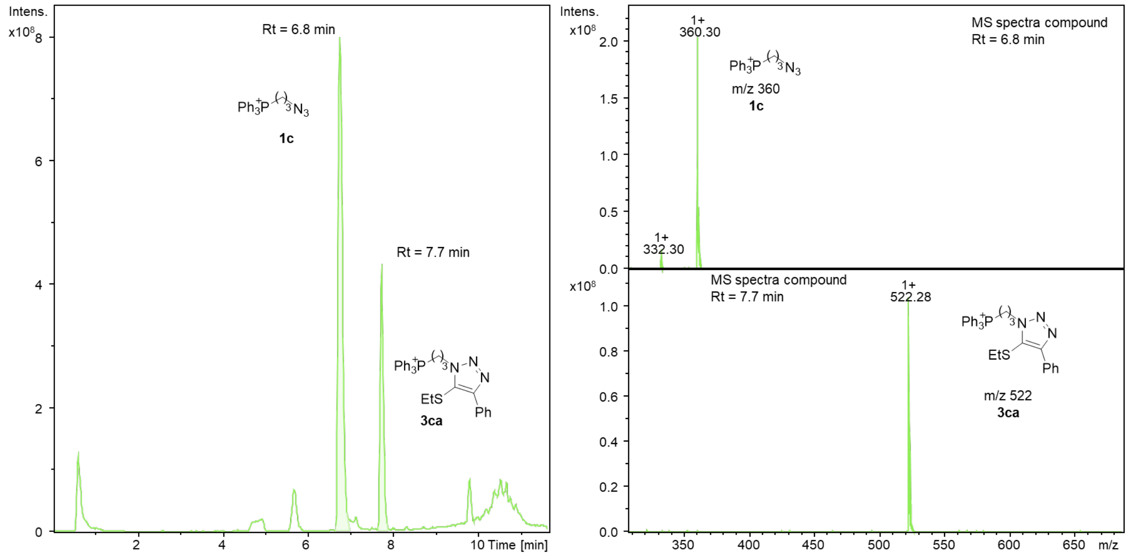

b)

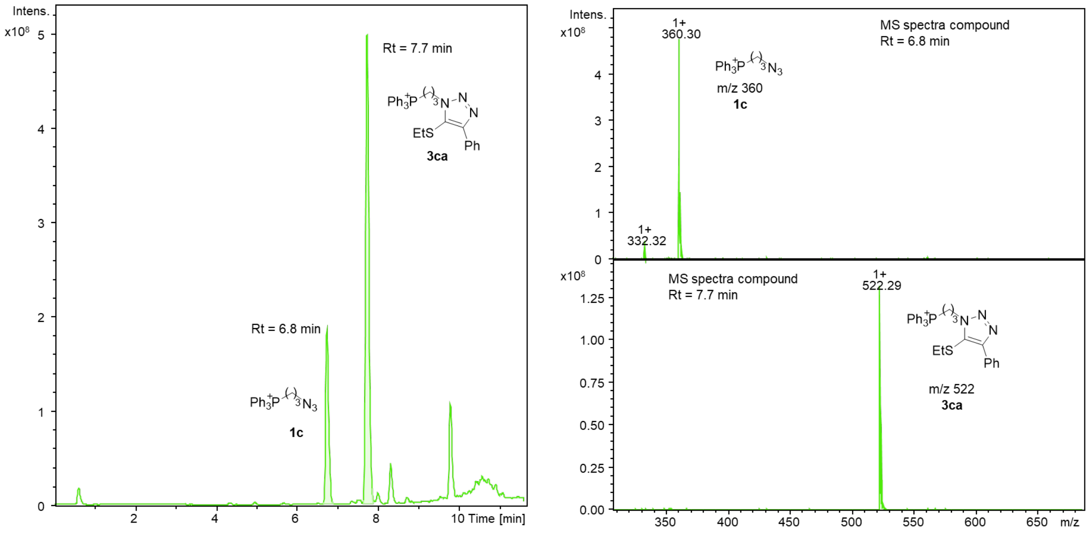

Figure S15. a) MS-chromatogram and MS-spectra for the indicated peaks in the supernatant b) MS-chromatogram and MS-spectra for the indicated peaks in the supernatant

Control experiments

Blank:

500 µL of a HeLa cell suspension (10^6 cells / mL) in DMEM-HEPES (without phenol-red) were transferred to a HPLC and kept at 37 ºC and stirred for 2 h at 80 rpm. After the indicated time 400 µL of the suspension were taken to Eppendorf vial and centrifuged at 10 000 rpm for 4 min. The supernatant was transferred to a HPLC vial diluted with 400 µL of MeOH (80%$_{v/v}$), coumarin was added as IS (4 µL of a stock solution 20 mM in DMSO) and analyzed by HPLC-MS. The remaining cell pellet was treated with 200 µL of MeOH (80% $_{v/v}$) and shaked at 1000 rpm for 5 min, followed by centrifugation at 10 000 rpm for 5 min. The resulting extract was transferred to a HPLC vial diluted with 200 µL of MeOH (80% $_{v/v}$), coumarin was added as IS (2 µL of a stock solution 20 mM in DMSO) and analyzed by HPLC-MS.

a)

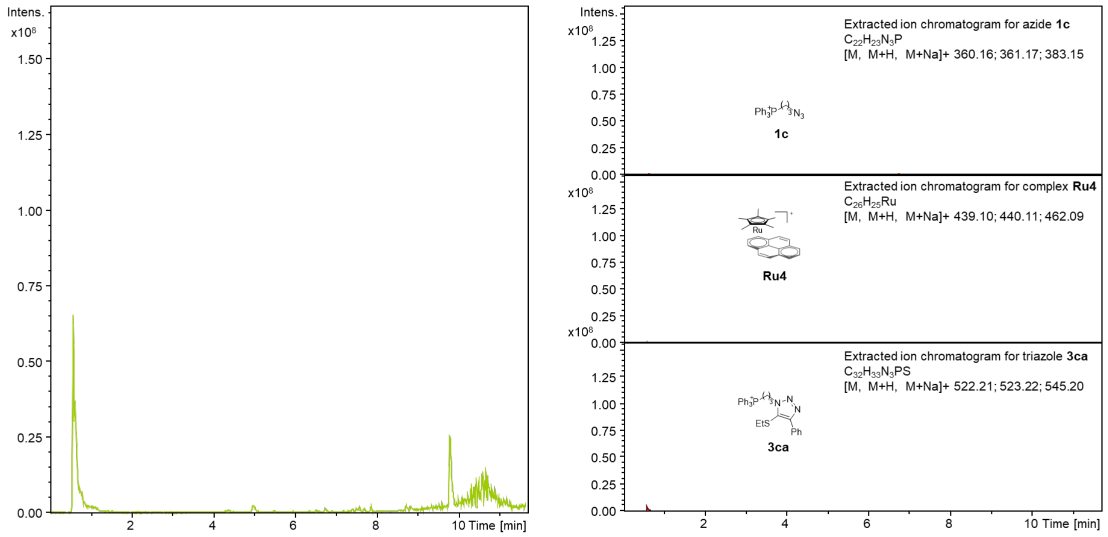

. b)

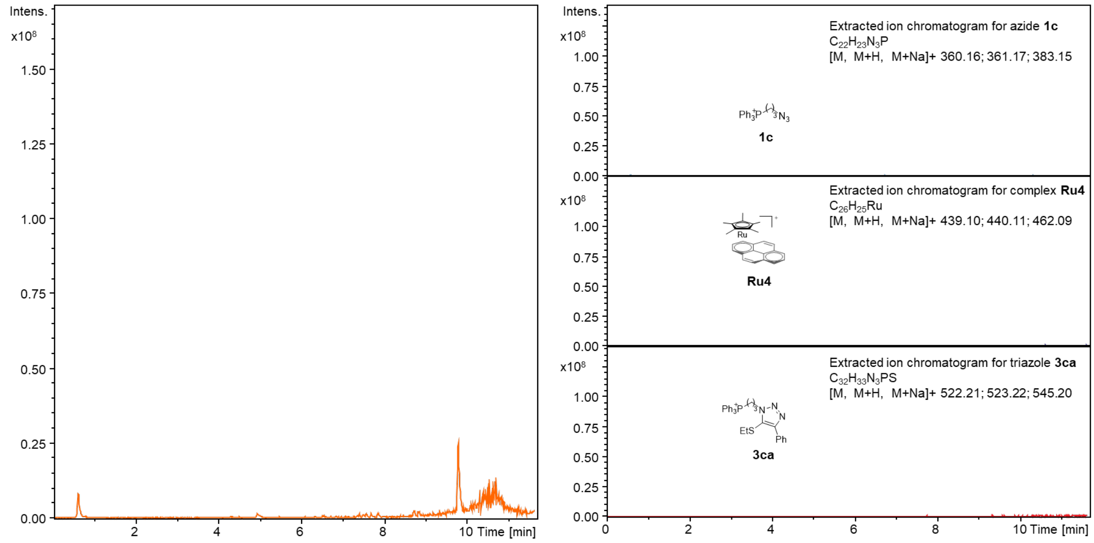

Figure S16. a) Blank MS-chromatogram of the supernatant. Extracted ion chromatograms for triazole **3ca**, azide **1c** and ruthenium complex **Ru4** for the supernatant of the blank; b) Blank MS-chromatogram of the supernatant. Extracted ion chromatograms for triazole **3ca**, azide **1c** and ruthenium complex **Ru4** for the methanolic extract of the blank.

Control reaction in the presence of Ru4, without irradiation

500 µL of a HeLa cell suspension (10^6 cells / mL) in DMEM-HEPES (without phenol-red) were transferred to a HPLC vial followed by sequential addition of the thioalkyne **2a** (10 µL, from a stock solution 40 mM in DMSO, 8.0 eq.), azide **1c** (2.5 µL, from a stock solution 20 mM in DMSO, 1.0 eq.) and **Ru4** (2.5 µL, from a stock solution 10 mM in DMSO, 0.5 eq.).The resulting suspension was kept at 37 ºC and stirred for 2 h at 80 rpm. After the indicated time 400 µL of the suspension were taken to Eppendorf vial and centrifuged at 10.000 rpm for 4 min. The supernatant was transferred to a HPLC vial diluted with 400 µL of MeOH ($80\%_{v/v}$), coumarin was added as IS (4 µL of a stock solution 20 mM in DMSO) and analyzed by HPLC-MS. The remaining cell pellet was treated with 200 µL of MeOH ($80\%_{v/v}$) and shaked at 1000 rpm for 5 min, followed by centrifugation at 10.000 rpm for 5 min. The resulting extract was transferred to a HPLC vial diluted with 200 µL of MeOH ($80\%_{v/v}$), coumarin was added as IS (2 µL of a stock solution 20 mM in DMSO) and analyzed by HPLC-MS.

a)

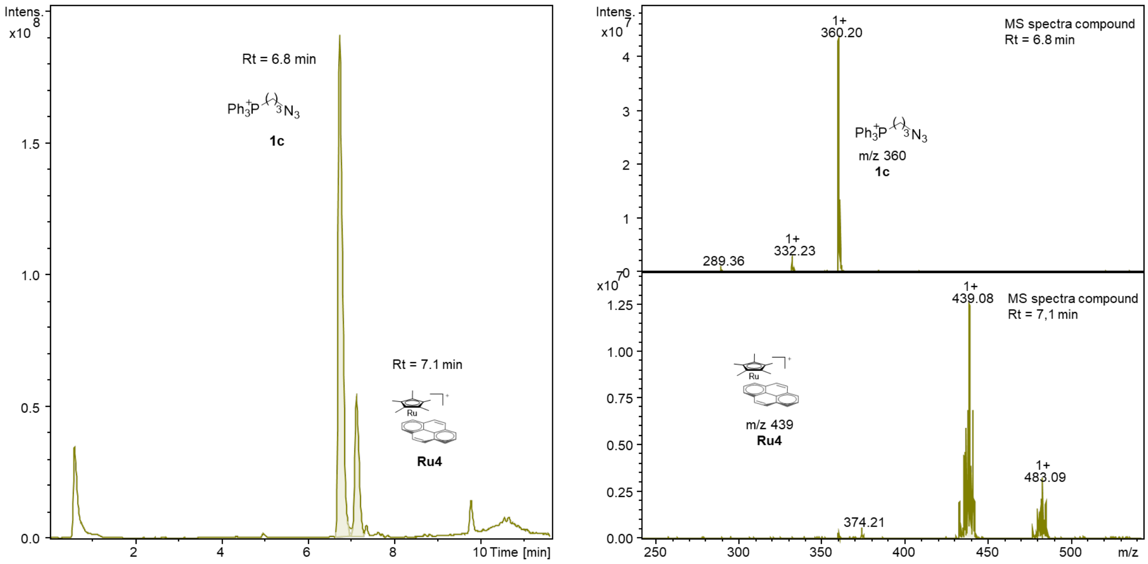

b)

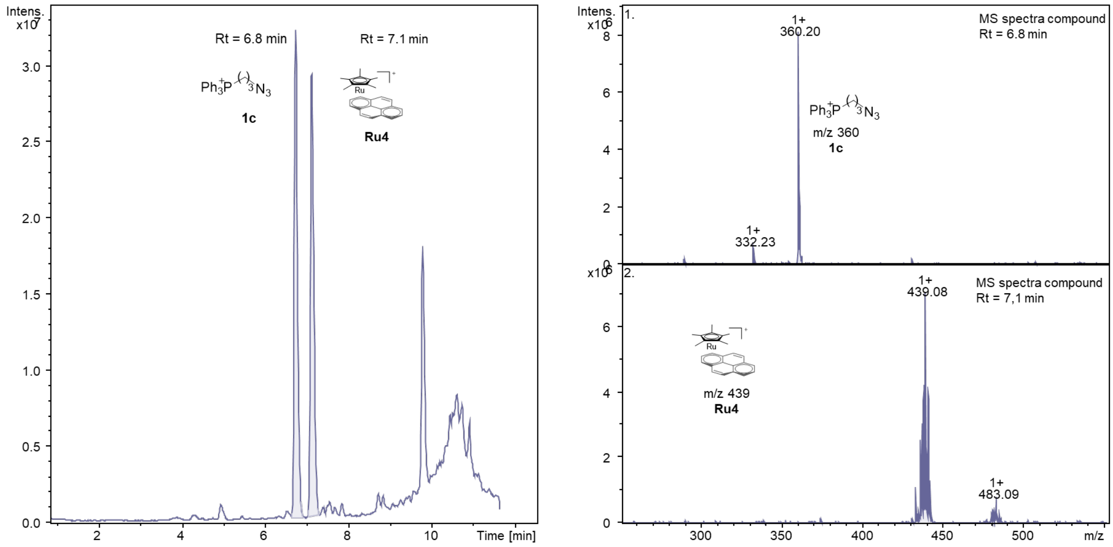

Figure S17. a) MS-chromatogram and MS-spectra for the indicated peaks in the supernatant. b) MS-chromatogram and MS-spectra for the indicated peaks in the methanolic extract.

Control reaction without Ru4 and without irradiation

500 µL of a HeLa cell suspension (10^6 cells / mL) in DMEM-HEPES (without phenol-red) were transferred to a HPLC vial followed by sequential addition of the thioalkyne **2a** (10 µL, from a stock solution 40 mM in DMSO, 8.0 eq.) and azide **1c** (2.5 µL, from a stock solution 20 mM in DMSO, 1.0 eq.) The resulting suspension was kept at 37 ºC and stirred for 2 h at 80 rpm. After the indicated time 400 µL of the suspension were taken to Eppendorf vial and centrifuged at 10.000 rpm for 4 min. The supernatant was transferred to a HPLC vial diluted with 400 µL of MeOH (80%$_{v/v}$), coumarin was added as IS (4 µL of a stock solution 20 mM in DMSO) and analyzed by HPLC-MS. The remaining cell pellet was treated with 200 µL of MeOH (80% $_{v/v}$) and shaked at 1000 rpm for 5 min, followed by centrifugation at 10 000 rpm for 5 min. The resulting extract was transferred to a HPLC vial diluted with 200 µL of MeOH (80% $_{v/v}$), coumarin was added as IS (2 µL of a stock solution 20 mM in DMSO) and analyzed by HPLC-MS.

a)

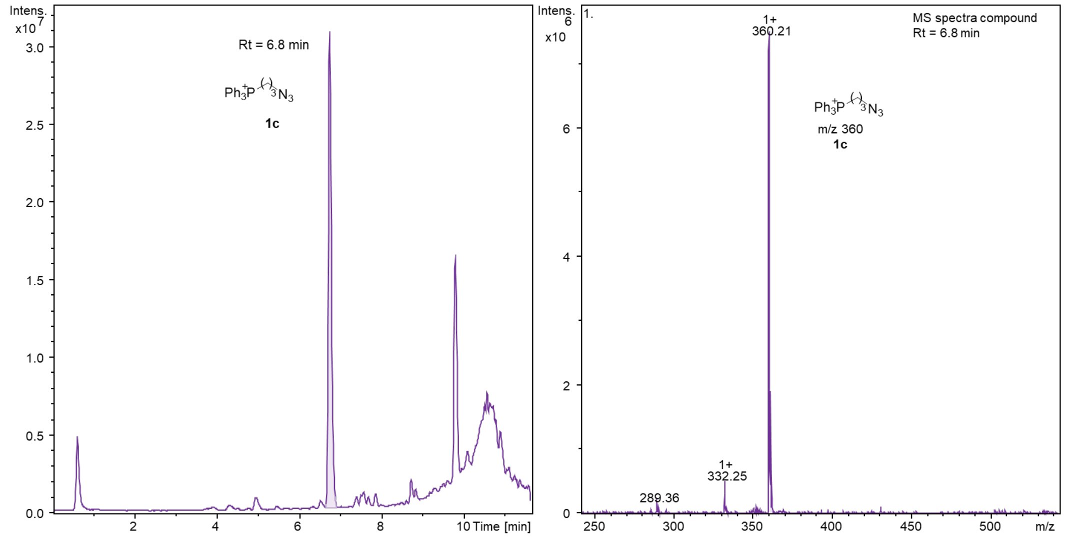

b)

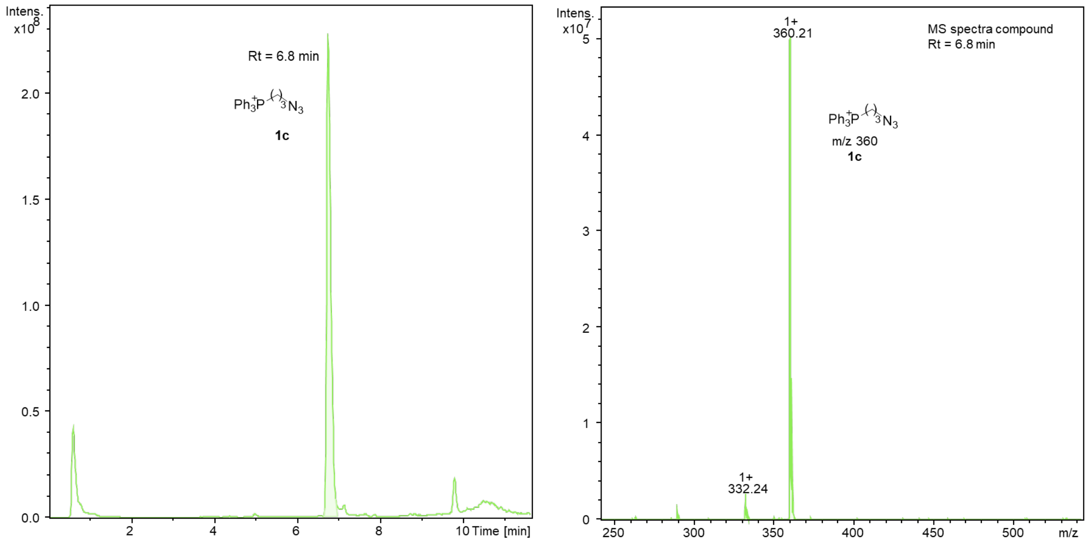

Figure S18. a) MS-chromatogram and MS-spectra for the indicated peaks in the supernatant. b) MS-chromatogram and MS-spectra for the indicated peaks in the methanolic extract

Control reaction without Ru4, with irradiation

500 μL of a HeLa cell suspension (10^6 cells / mL) in DMEM-HEPES (without phenol-red) were transferred to a HPLC vial followed by sequential addition of the thioalkyne **2a** (10 μL, from a stock solution 40 mM in DMSO, 8.0 eq.) and azide **1c** (2.5 μL, from a stock solution 20 mM in DMSO, 1.0 eq.) The resulting suspension was irradiated at 365 nm for 10 min, then kept at 37 ºC and stirred for 2 h at 80 rpm. After the indicated time 400 μL of the suspension were taken to Eppendorf vial and centrifuged at 10.000 rpm for 4 min. The supernatant was transferred to a HPLC vial diluted with 400 μL of MeOH (80%$_{v/v}$), coumarin was added as IS (4 μL of a stock solution 20 mM in DMSO) and analyzed by HPLC-MS. The remaining cell pellet was treated with 200 μL of MeOH (80% $_{v/v}$) and shaked at 1000 rpm for 5 min, followed by centrifugation at 10.000 rpm for 5 min. The resulting extract was transferred to a HPLC vial diluted with 200 μL of MeOH (80% $_{v/v}$), coumarin was added as IS (2 μL of a stock solution 20 mM in DMSO) and analyzed by HPLC-MS.

a)

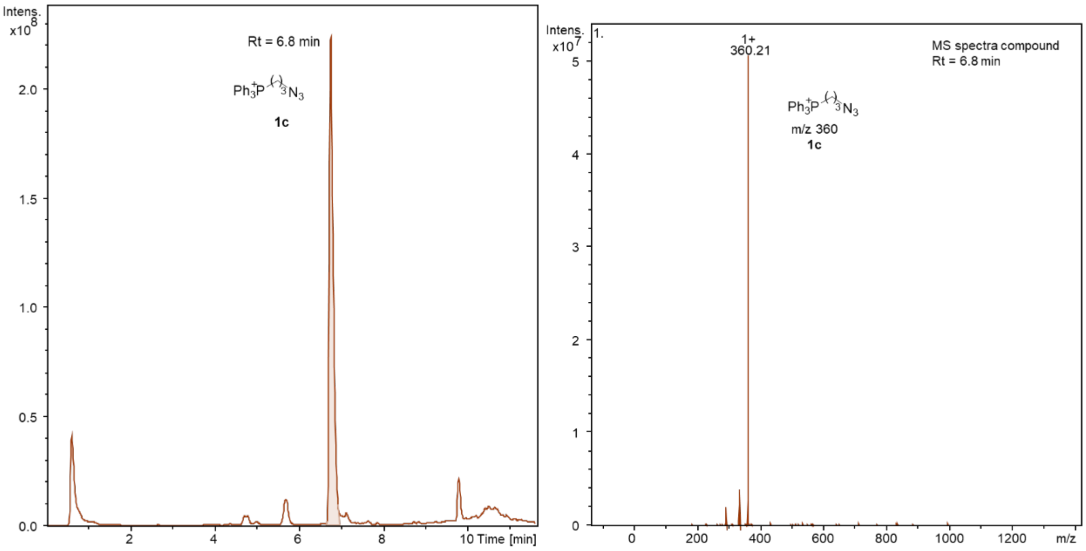

b)

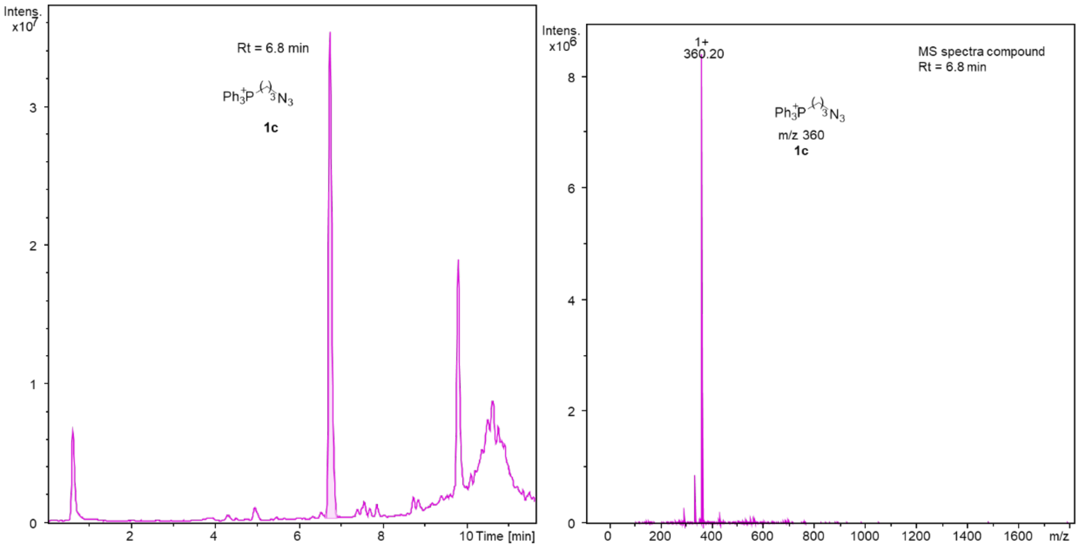

Figure S19. a) MS-chromatogram and MS-spectra for the indicated peaks in the supernatant. b) MS-chromatogram and MS-spectra for the indicated peaks in the methanolic extract.

Control with the product 3ca

500 µL of a HeLa cell suspension (10^6 cells / mL) in DMEM-HEPES (without phenol-red) were transferred to a HPLC vial followed addition of the triazole **3ca** (2.5 µL, from a stock solution 20 mM in DMSO, 8.0 eq.) The resulting suspension was kept at 37 ºC and stirred for 2 h at 80 rpm. After the indicated time 400 µL of the suspension were taken to Eppendorf vial and centrifuged at 10 000 rpm for 4 min. The supernatant was transferred to a HPLC vial diluted with 400 µL of MeOH (80%$_{v/v}$), coumarin was added as IS (4 µL of a stock solution 20 mM in DMSO) and analyzed by HPLC-MS. The remaining cell pellet was treated with 200 µL of MeOH (80% $_{v/v}$) and shaked at 1000 rpm for 5 min, followed by centrifugation at 10.000 rpm for 5 min. The resulting extract was transferred to a HPLC vial diluted with 200 µL of MeOH (80% $_{v/v}$), coumarin was added as IS (2 µL of a stock solution 20 mM in DMSO) and analyzed by HPLC-MS.

a)

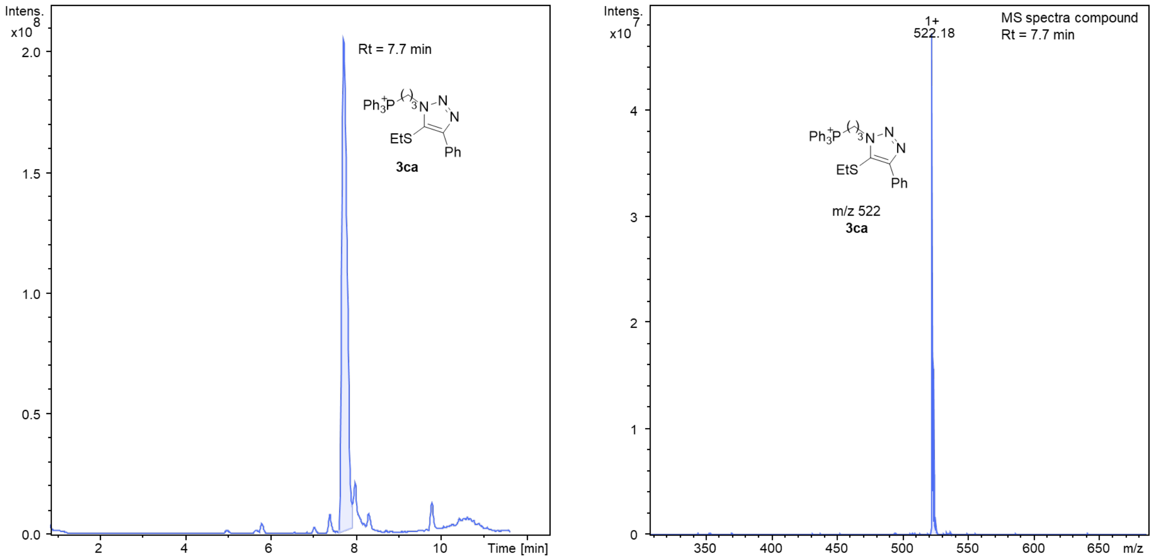

b)

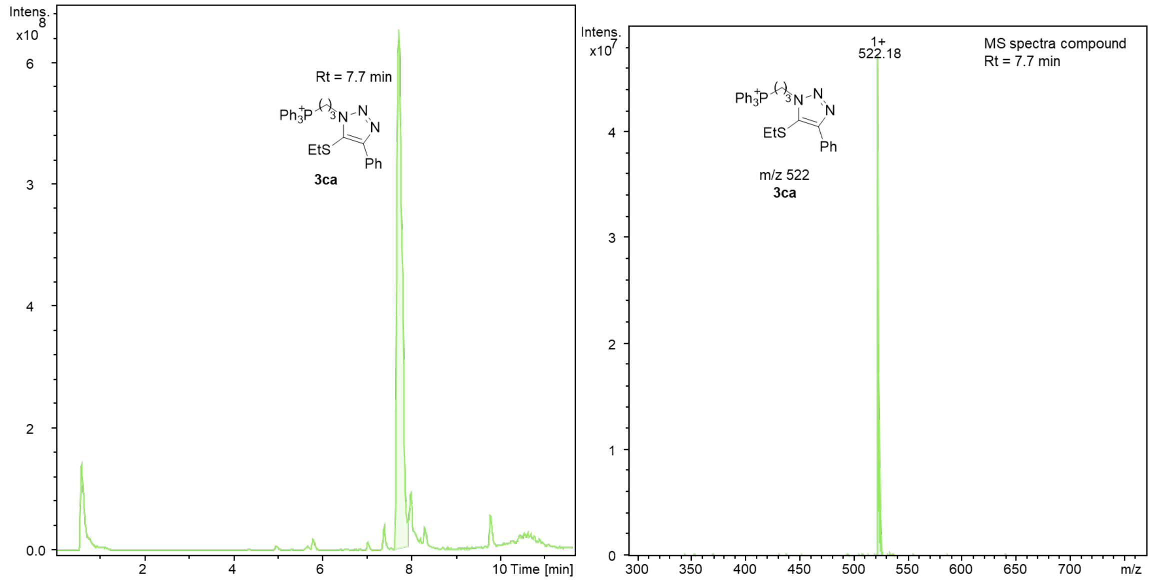

Figure S20. a) MS-chromatogram and MS-spectra for the indicated peaks in the supernatant. b) MS-chromatogram and MS-spectra for the indicated peaks in the methanolic extract.

Cell Viability assays

Viability assays were performed to determine the cellular toxicity of the catalytic reaction

Hela cells were seeded on a 96 well plate at a concentration of 50,000 cells/ml. 24 h later, cells were incubated with 100 μM of **Ru2** or left untreated. In parallel, to replicate the conditions of the photoactivatable reactions, cells were treated with 100 μM of the **Ru4** and irradiated for 10 min with UV light. All incubations were performed in serum-free DMEM-HEPES for 18h. Cell viability was then analyzed by MTT assay. To perform this assay, incubation media was changed by serum-free DMEM-HEPES containing Thiazolyl Blue Tetrazolium Bromide (Sigma) at a final concentration of 0.5 mg/ml and cells were incubated for 4 h to allow the formation of formazan precipitates by metabolically active cells. A detergent solution of 10% SDS (sodium dodecyl sulphate) and 0.01 M HCl was then added and the plate was incubated overnight at room temperature to allow the solubilization of the precipitates. The quantity of formazan in each well (directly proportional to the number of viable cells) was measured by recording changes in absorbance at 570 nm in a microtiter plate reading spectrophotometer (Tecan Infinite 200 PRO). As shown in Figure S21, incubation of cells with **Ru2** even at long periods (24h), exceedingly greater that the time needed by the catalytic reaction (< 2h), only resulted in a decrease of about 30% in cell viability compared to untreated cells. Treatment of cells with **Ru4** and UV irradiation in the conditions required for the catalysis resulted in a marked toxicity.

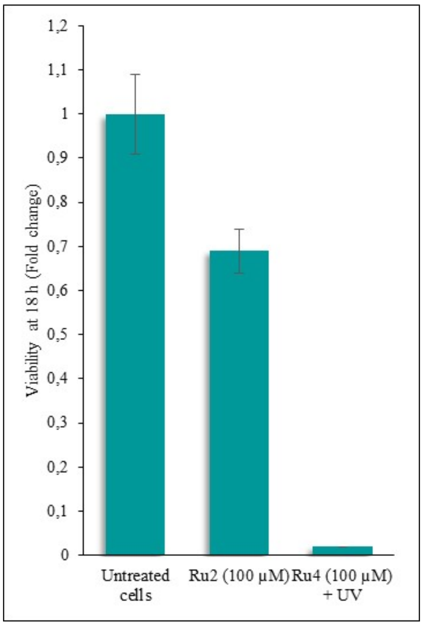

Figure S21. Viability of cells after 24h of treatment with **Ru2** and with **Ru4**+irradiation for 15 min. Results are indicated as the fold change on cell viability with respect to untreated cells. Error bars represent the standard deviation of three replicates.

12. References

[1]. K.-C. Chang, I.-H. Su, A. Senthilvelan, W.-S. Chung, *Org. Lett.* **2007**, *9*, 3363–3366.

[2]. R. M. Pinto, R. I. Olariu, J. Lameiras, F. T. Martins, A. A. Dias, G. J. Langley, P. Rodrigues, C. D. Maycock, J. P. Santos, M. F. Duarte, M. T. Fernandez, M. L. Costa, *Journal of Molecular Structure* **2010**, *980*, 163–171.

[3]. X.-L. Liu, L.-Y. Niu, Y.-Z. Chen, M.-L. Zheng, Y. Yang, Q.-Z. Yang, *Org. Biomol.Chem.***2017**, *15*, 1072–1075.

[4]. D. González-Calderón, M. A. Morales-Reza, E. Díaz-Torres, A. Fuentes-Benítes, C. González-Romero, *RSC Adv.* **2016**, *6*, 83547–83550.

[5]. N. Riddell, W. Tam, *J. Org. Chem.* **2006**, *71*, 1934–1937.

[6]. C. Eller, G. Kehr, C. G. Daniliuc, R. Fröhlich, G. Erker, *Organometallics* **2013**, *32*, 384–386.

[7]. W. Zheng, F. Zheng, Y. Hong, L. Hu, *Heteroatom Chemistry* **2012**, *23*, 105–110.

[8]. A. Naghipour, Z. H. Ghasemi, D. Morales-Morales, J. M. Serrano-Becerra, C. M. Jensen, *Polyhedron* **2008**, *27*, 1947–1952.

[9]. R. E. Beveridge, R. A. Batey, *Org. Lett.* **2013**, *15*, 3086–3089.

[10]. P. Destito, J. R. Couceiro, H. Faustino, F. López, J. L. Mascareñas, *Angew. Chem. Int. Ed.* **2017**, *56*, 10766–10770.

[11]. B. T. Loughrey, B. V. Cunning, P. C. Healy, C. L. Brown, P. G. Parsons, M. L. Williams, *Chem. Asian J.* **2012**, *7*, 112–121.

[12]. A. M. McNair, K. R. Mann, *Inorganic Chemistry* **1986**, *25*, 2519–2527.

13. NMR Spectra

η5-(Pentamethylcyclopentadienyl)-η6-(pyrene-1-sulfonate) ruthenium(II) sodium hexafluorophosphate (Ru5)

^{1}H (Methanol-*d4*, 300 MHz)

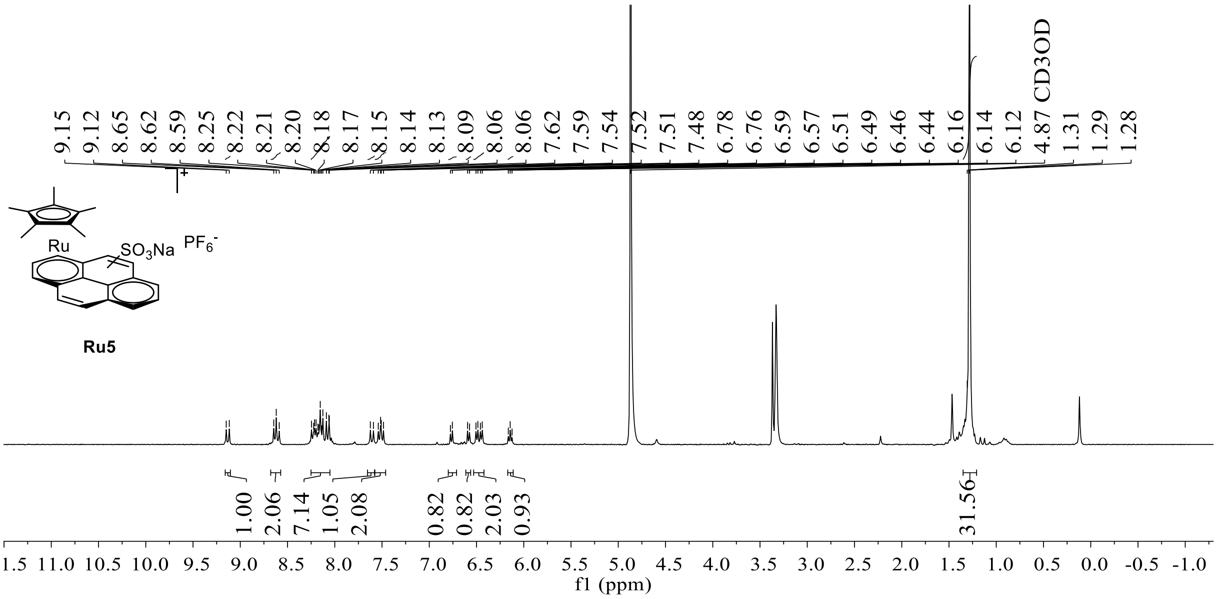

^{13}C (Methanol-*d4*, 75 MHz)

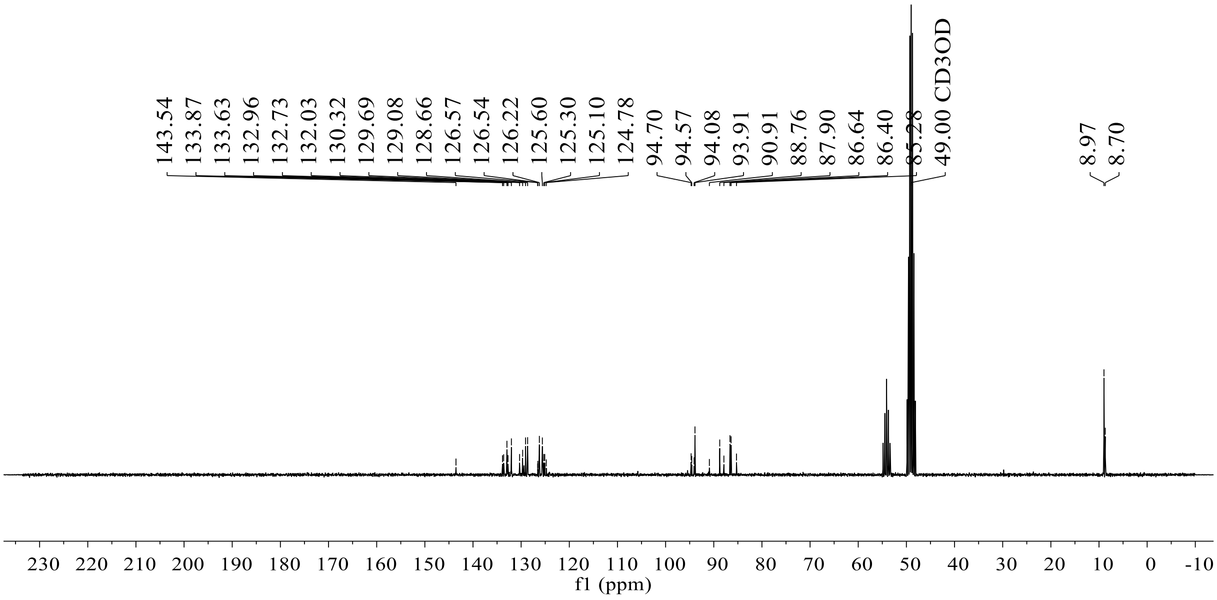

Ruthenium complex Ru2'

^{1}H (CD_2Cl_2, 300 MHz)

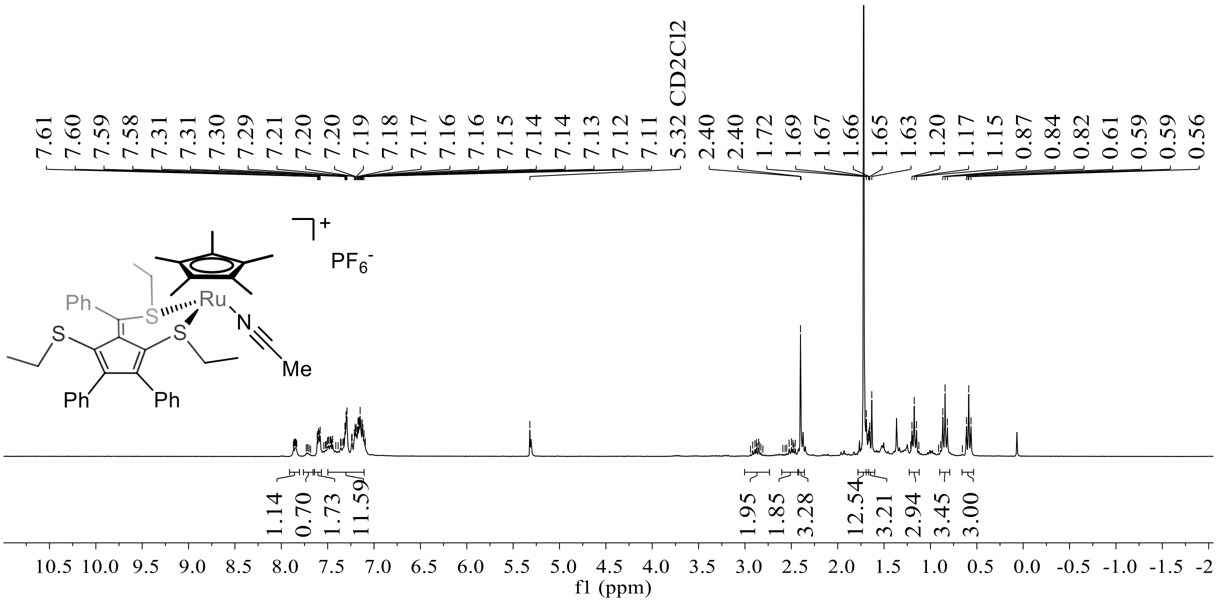

^{13}C (CD_2Cl_2, 75 MHz)

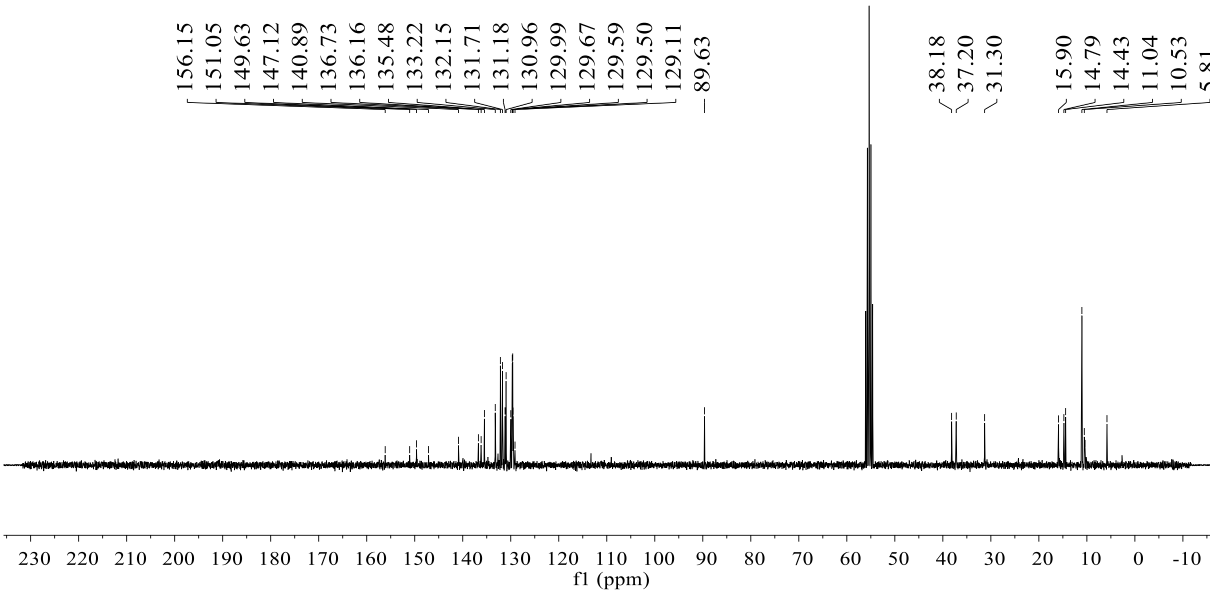

(3-(Azidomethyl)benzyl) triphenylphosphonium bromide (1d)

^1H NMR ($CDCl_3$, 300 MHz)

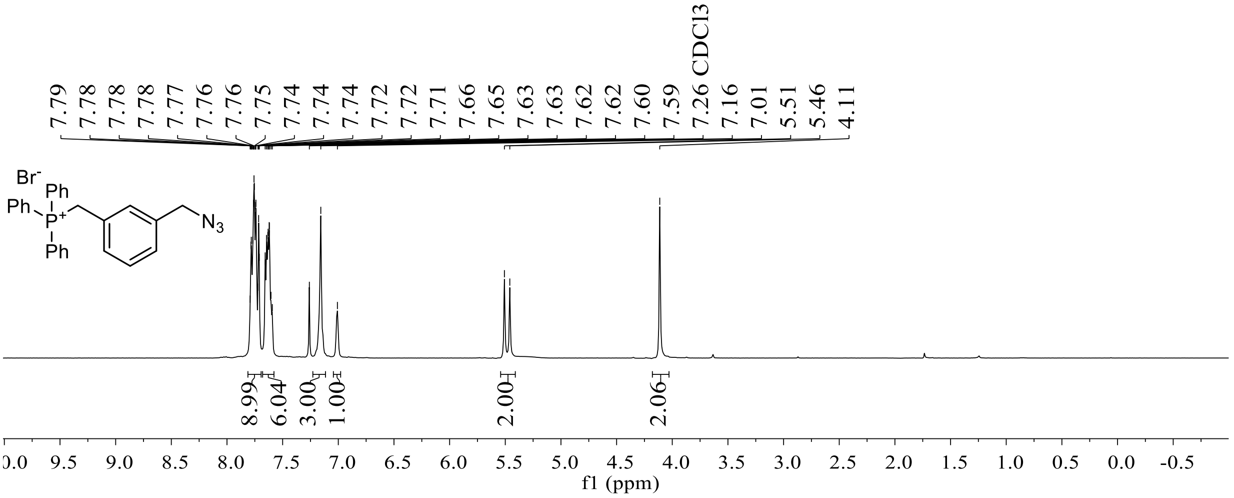

^{13}C NMR ($CDCl_3$, 75 MHz)

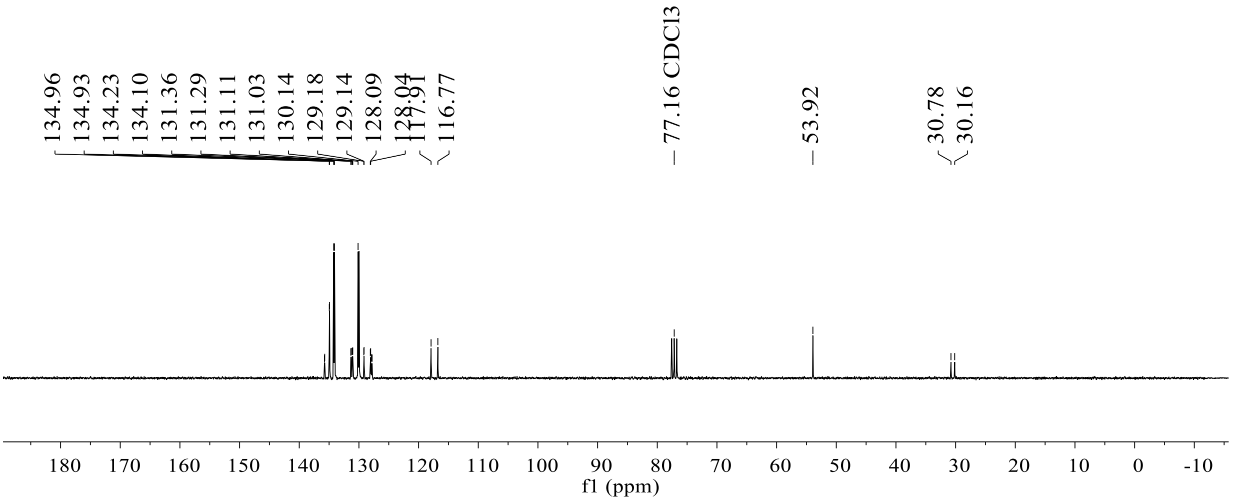

^{13}C-DEPT-135 ($CDCl_3$, 75 MHz)

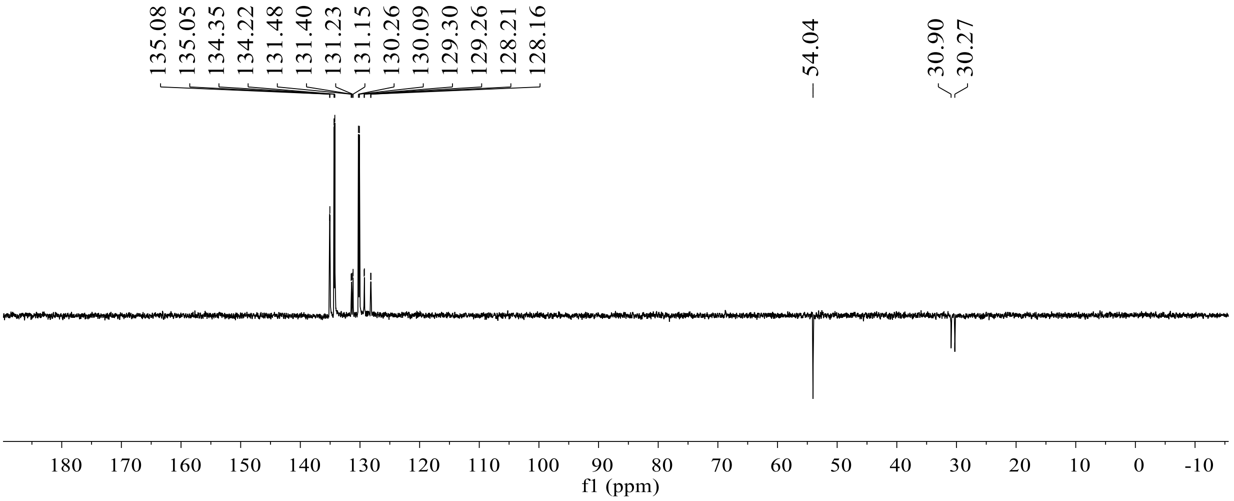

Benzyl(5-phenylpent-1-yn-1-yl)sulfane (2c)

^{1}H ($CDCl_3$, 300 MHz)

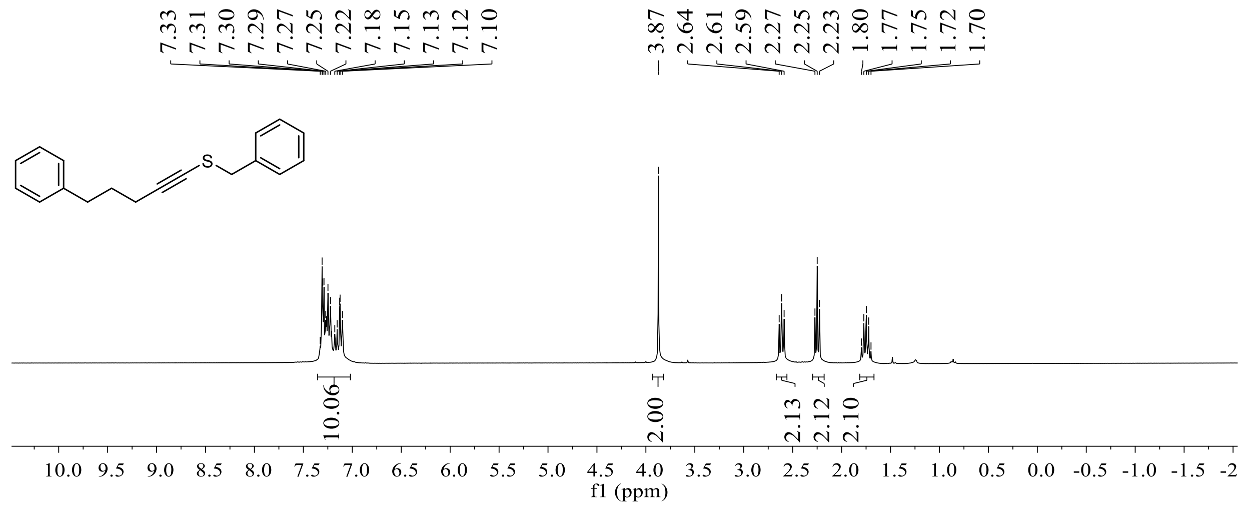

^{13}C ($CDCl_3$, 75 MHz)

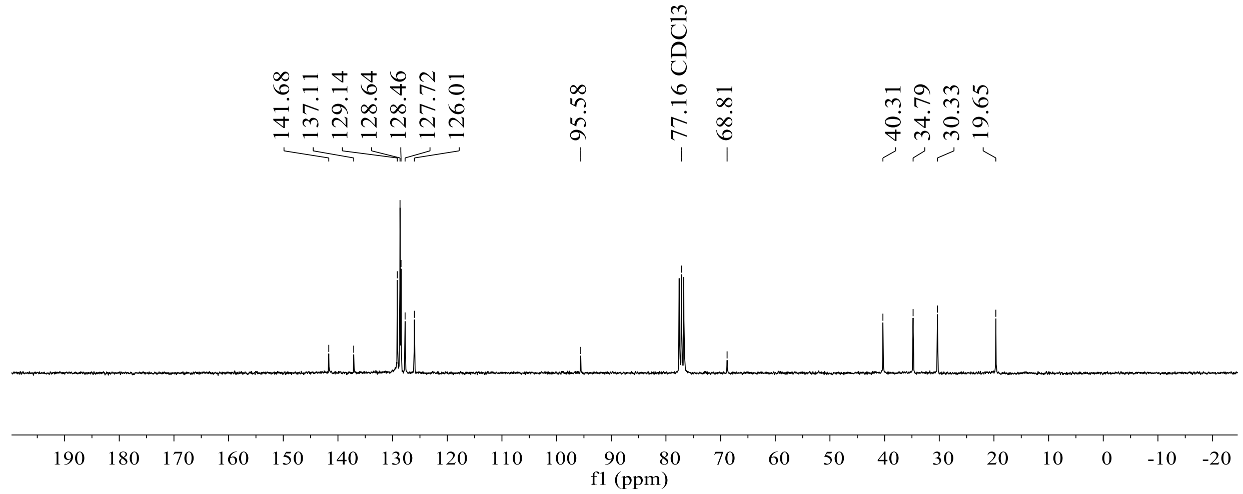

^{13}C-DEPT-135 ($CDCl_3$, 75 MHz)

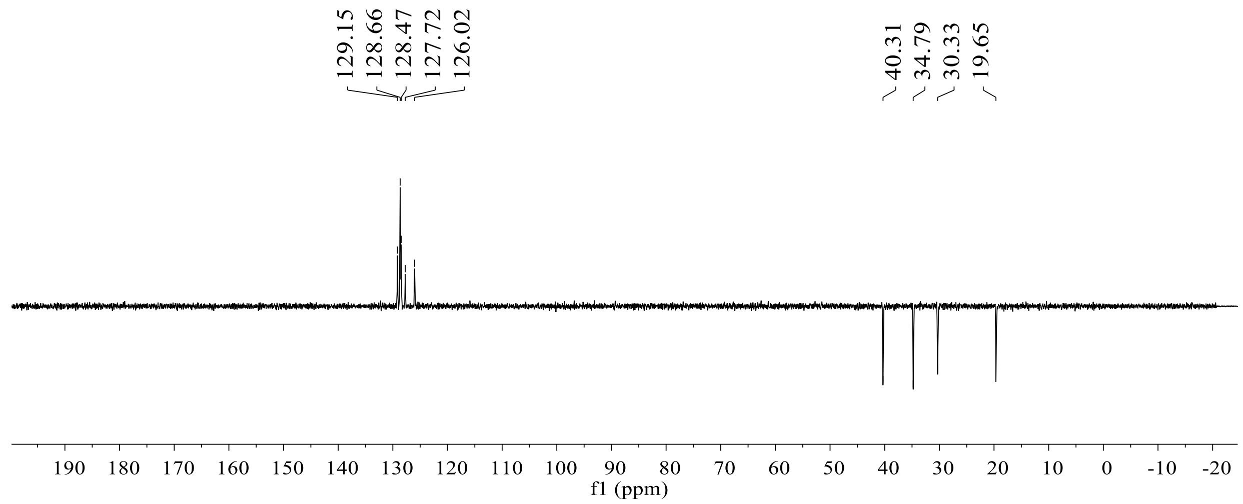

tert-Butyl((7-(ethylthio)hept-6-yn-1-yl)oxy)dimethylsilane.

^{1}H ($CDCl_3$, 75 MHz)

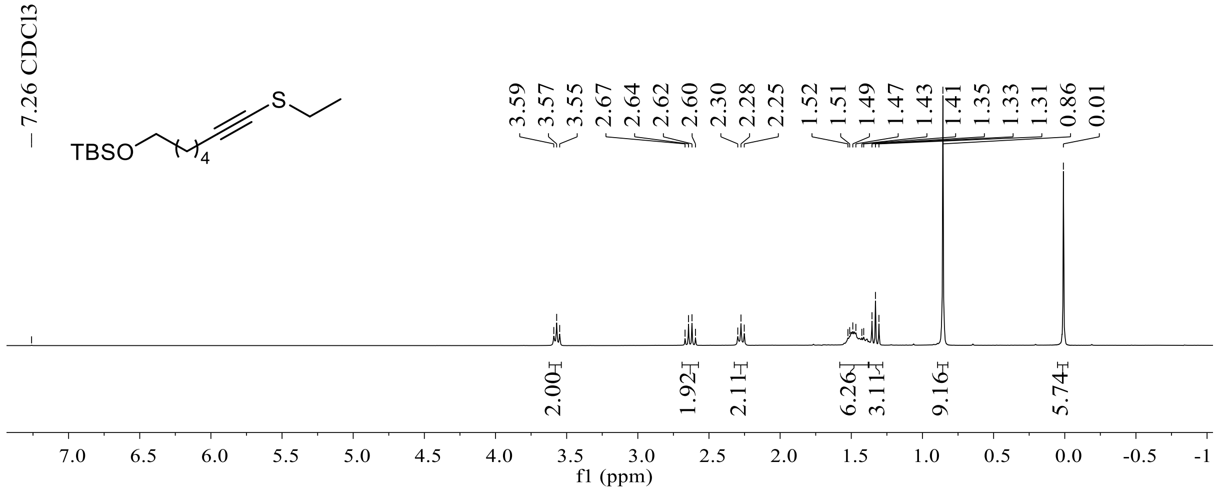

^{13}C ($CDCl_3$, 75 MHz)

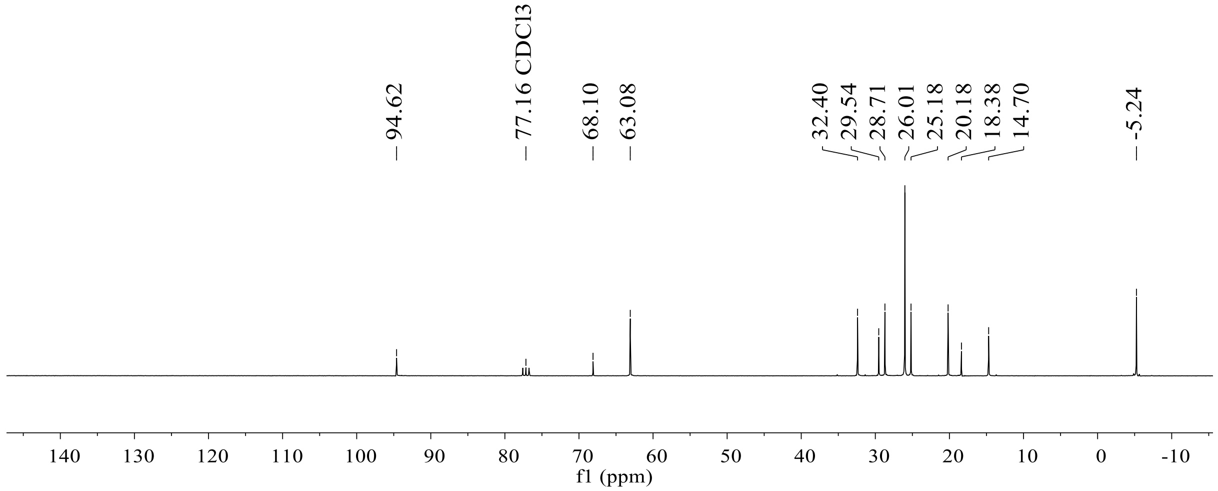

^{13}C-DEPT-135 ($CDCl_3$, 75 MHz)

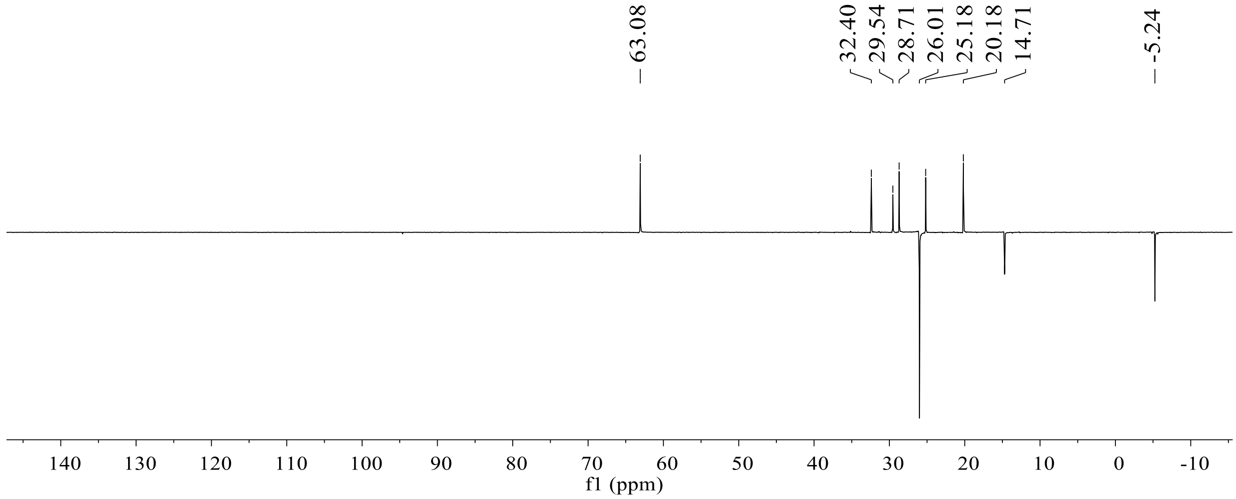

7-(Ethylthio)hept-6-yn-1-ol.

^{1}H (CDCl$_3$, 300 MHz)

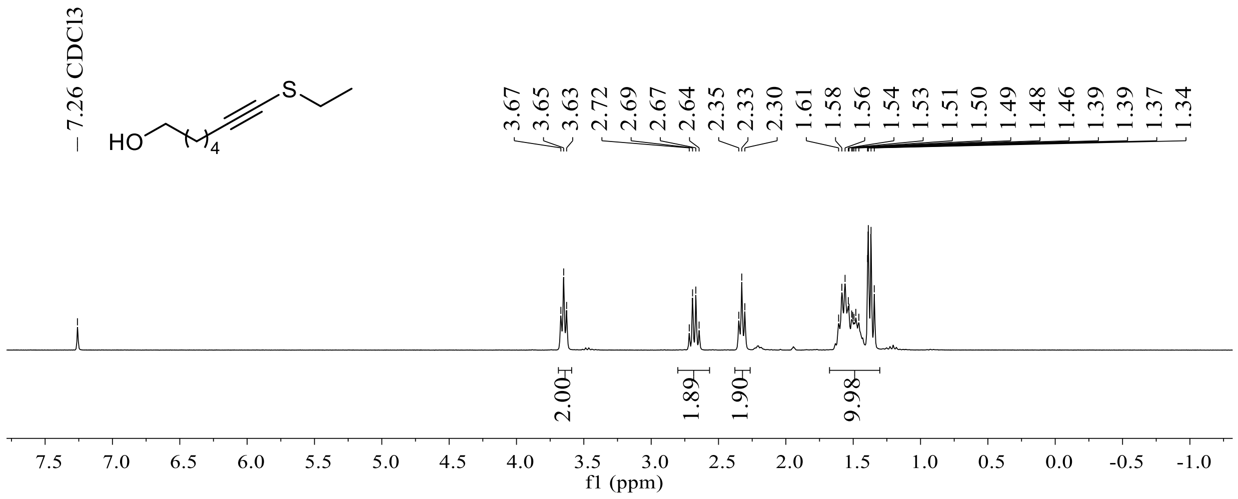

^{13}C (CDCl$_3$, 75 MHz)

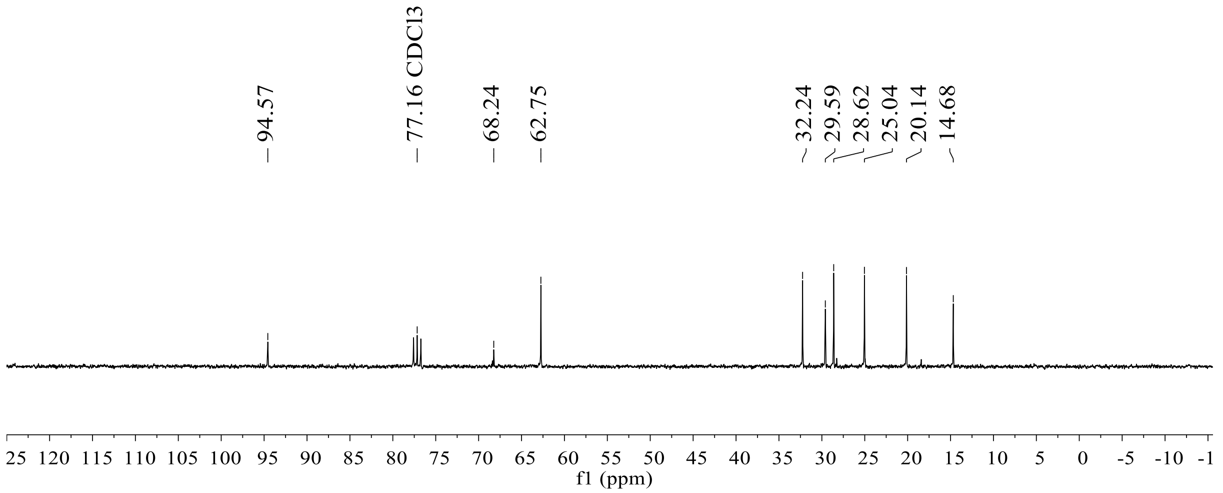

^{13}C-DEPT-135 (CDCl$_3$, 75 MHz)

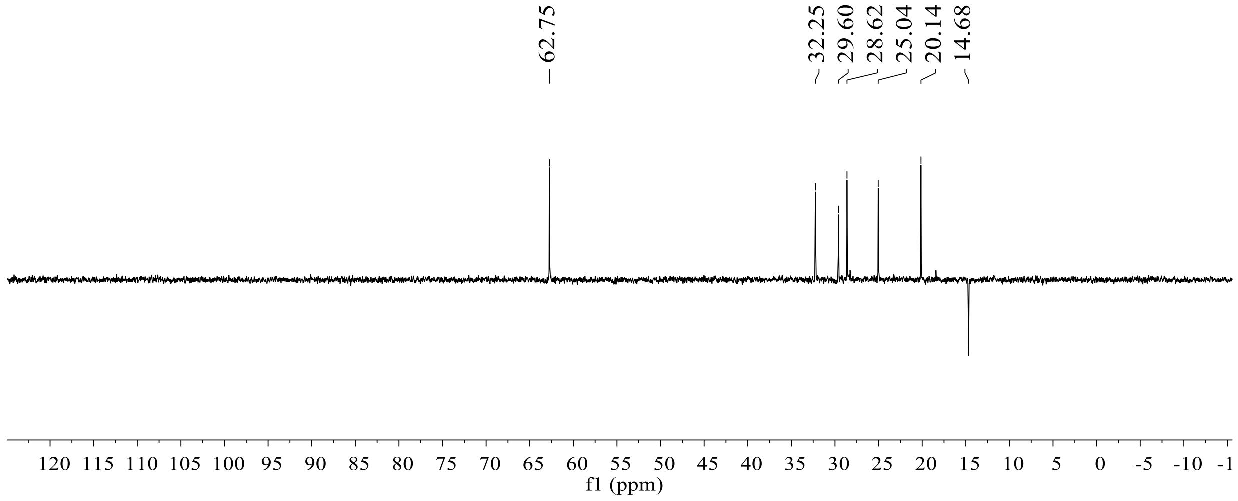

_N_-(6-(Diethylamino)-9-(2-(((7-(ethylthio)hept-6-yn-1-yl)oxy)carbonyl)phenyl)-3H-xanthen-3-ylidene)-N-ethylethanaminium chloride. (Rhodamine-thioalkyne) (2i)

^{1}H ($CDCl_3$, 500 MHz)

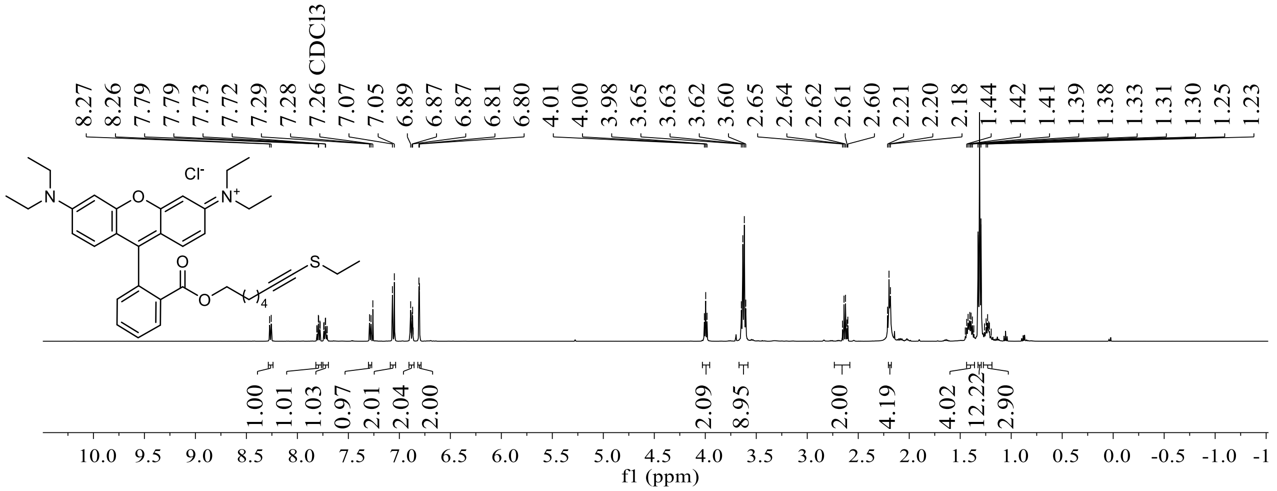

^{13}C ($CDCl_3$, 126 MHz)

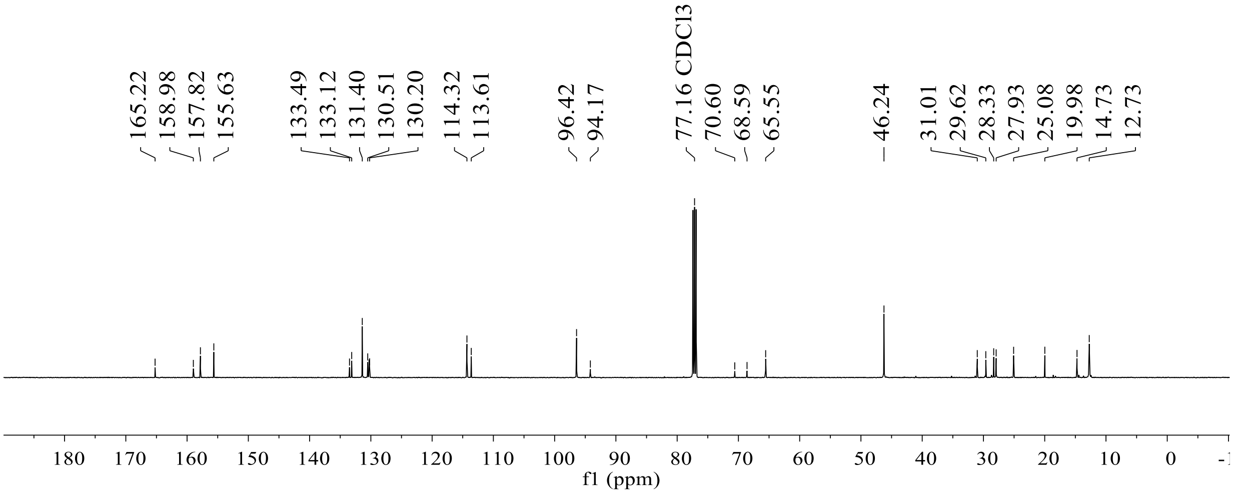

13C-DEPT-135 ($CDCl_3$, 126 MHz)

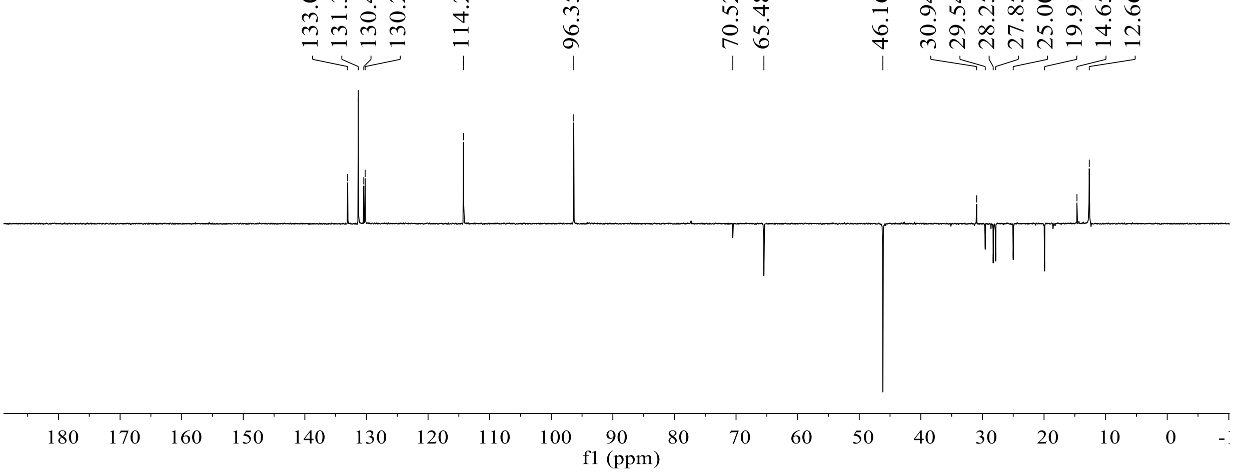

5-(Ethylthio)-4-phenyl-1-(p-methylbenzyl)-1H-1,2,3-triazole (3ba)

^{1}H ($CDCl_3$, 300 MHz)

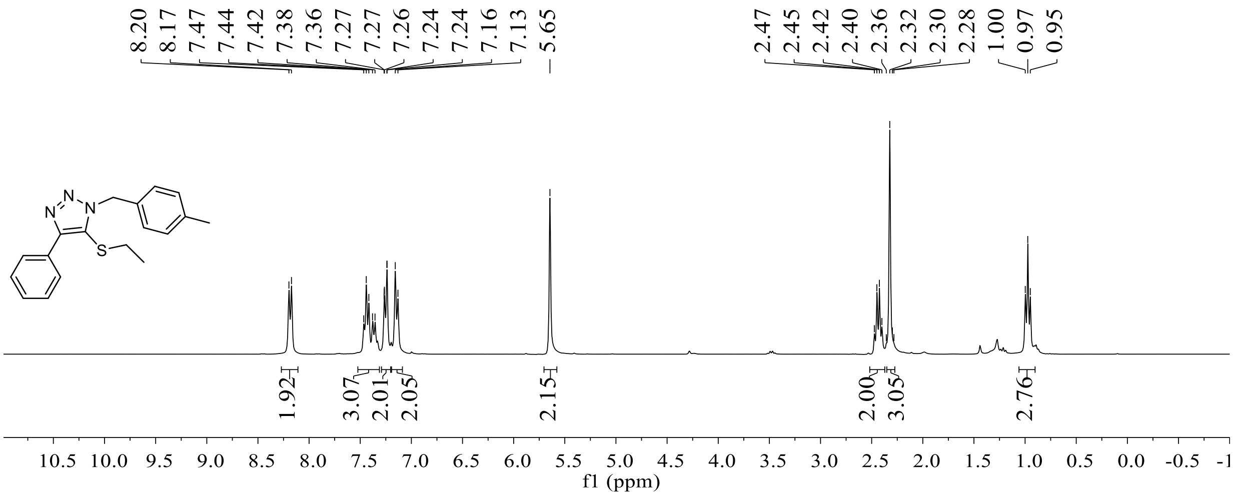

^{13}C ($CDCl_3$, 75 MHz)

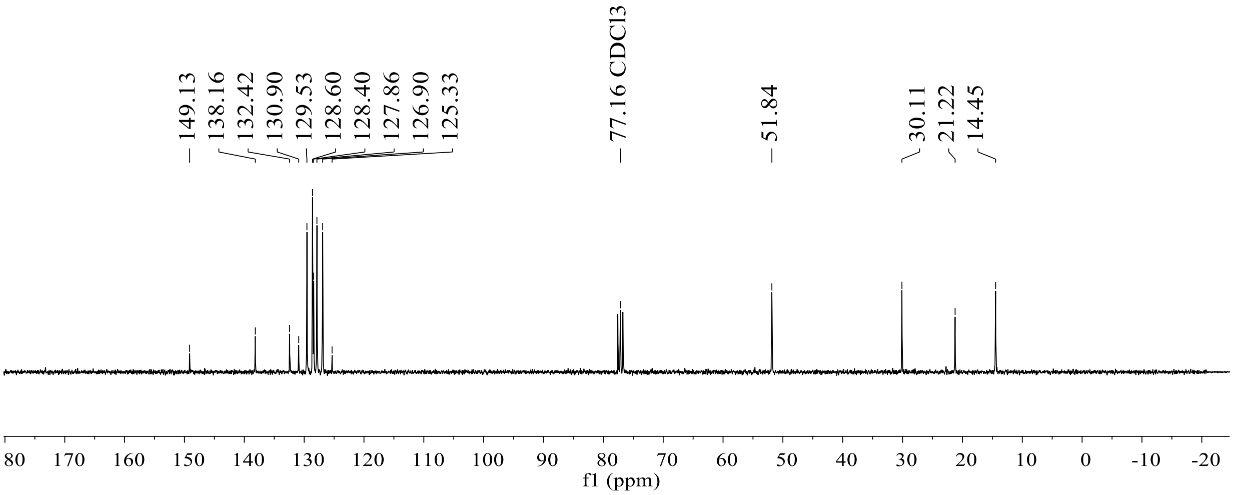

^{13}C-DEPT-135 ($CDCl_3$, 75 MHz)

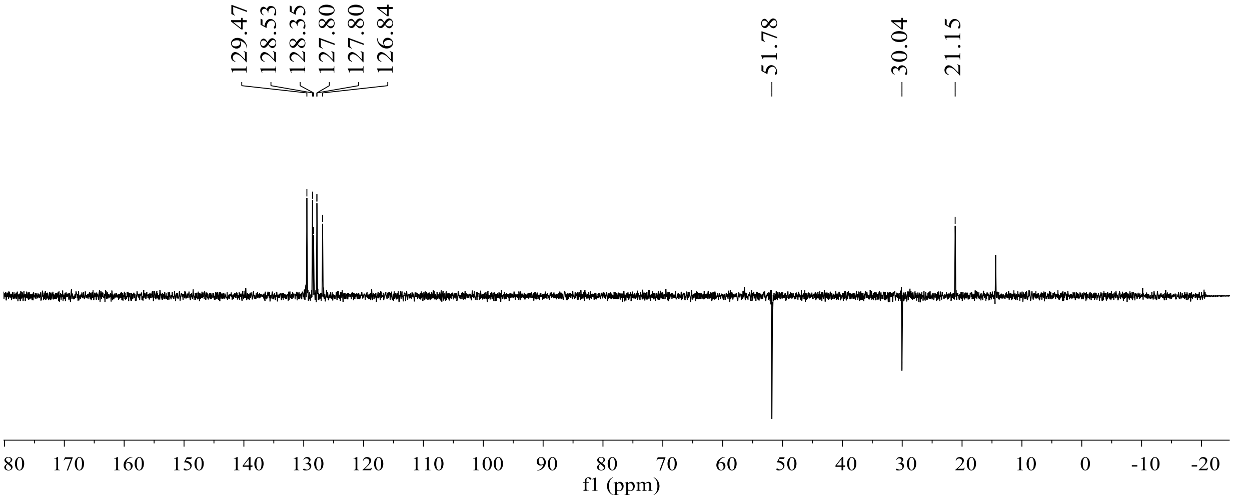

(4-(5-(Ethylthio)-4-phenyl-1H-1,2,3-triazol-1-yl)butyl)triphenylphosphonium bromide (3ca)

^{1}H ($CDCl_3$, 300 MHz)

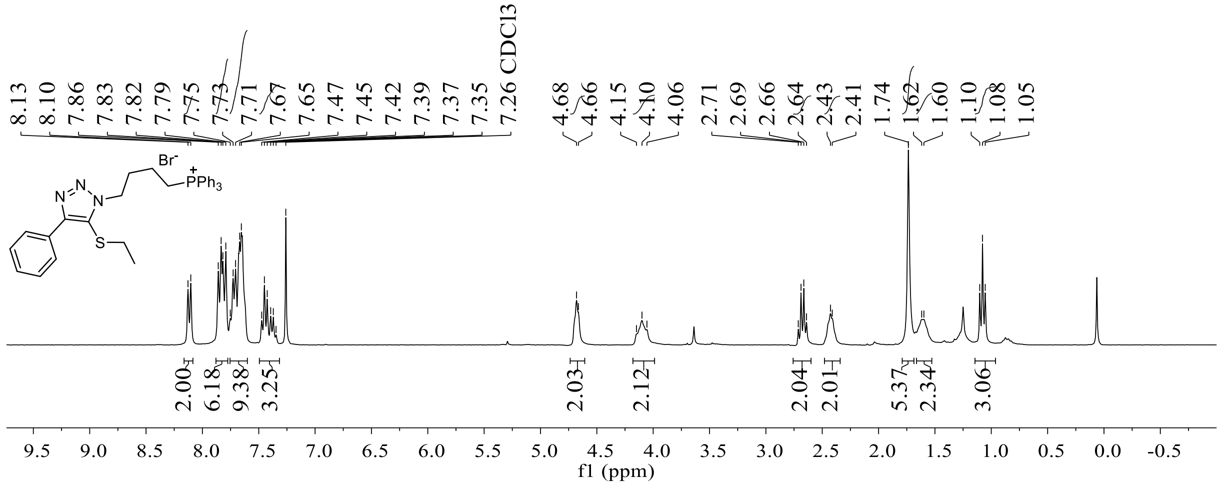

^{13}C ($CDCl_3$, 75 MHz)

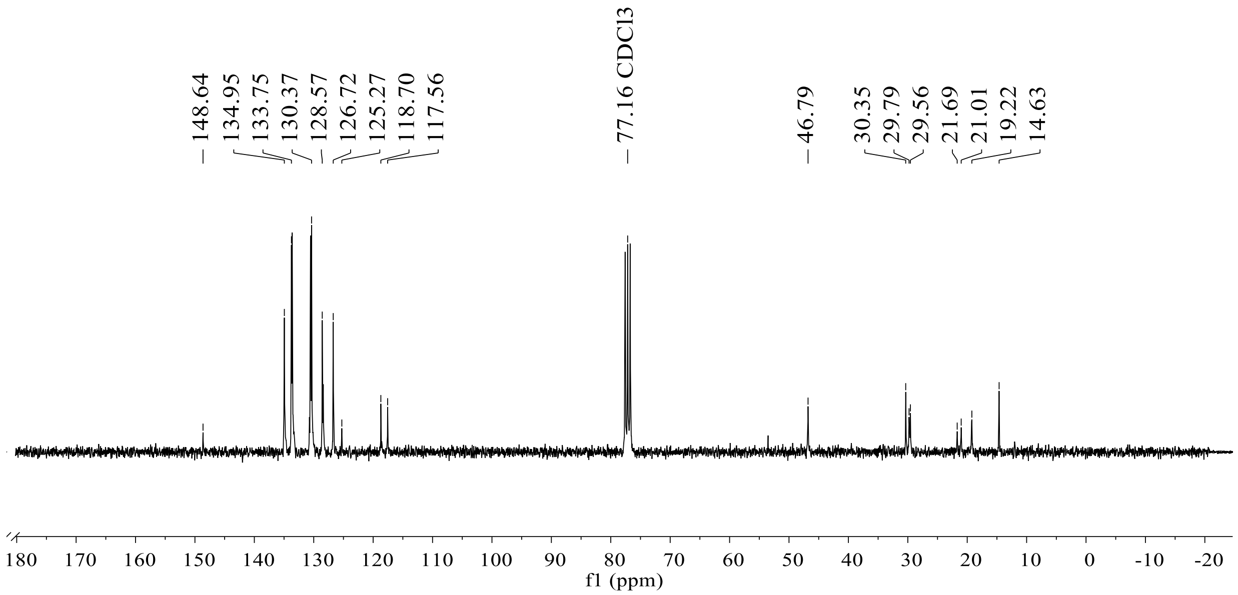

^{13}C-DEPT-135 ($CDCl_3$, 75 MHz)

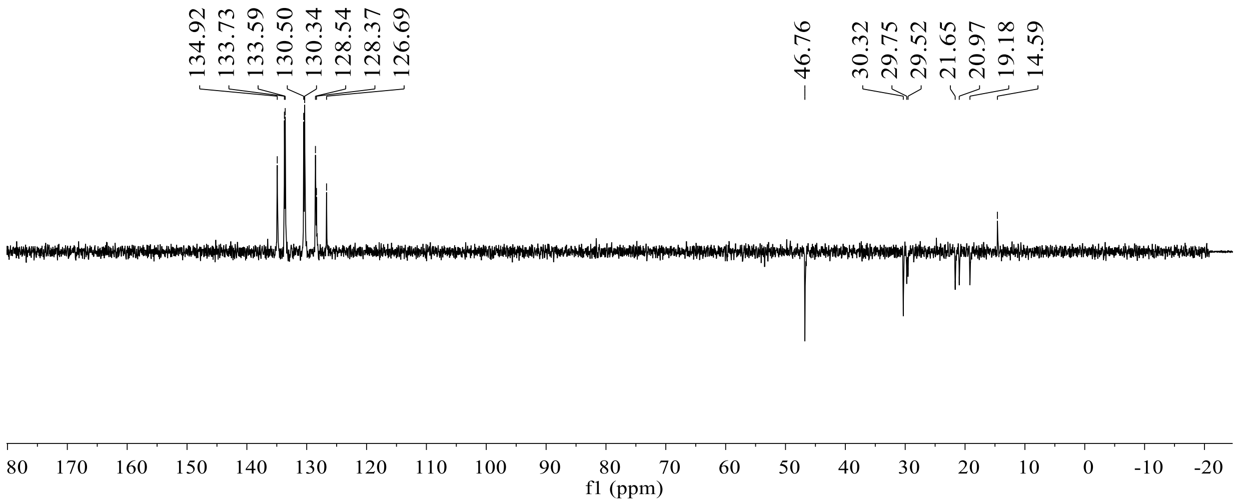

(3-((5-(Ethylthio)-4-phenyl-1H-1,2,3-triazol-1-yl) methyl)benzyl) triphenyl-phosphonium bromide(3da)

^{1}H (CDCl3, 300 MHz)

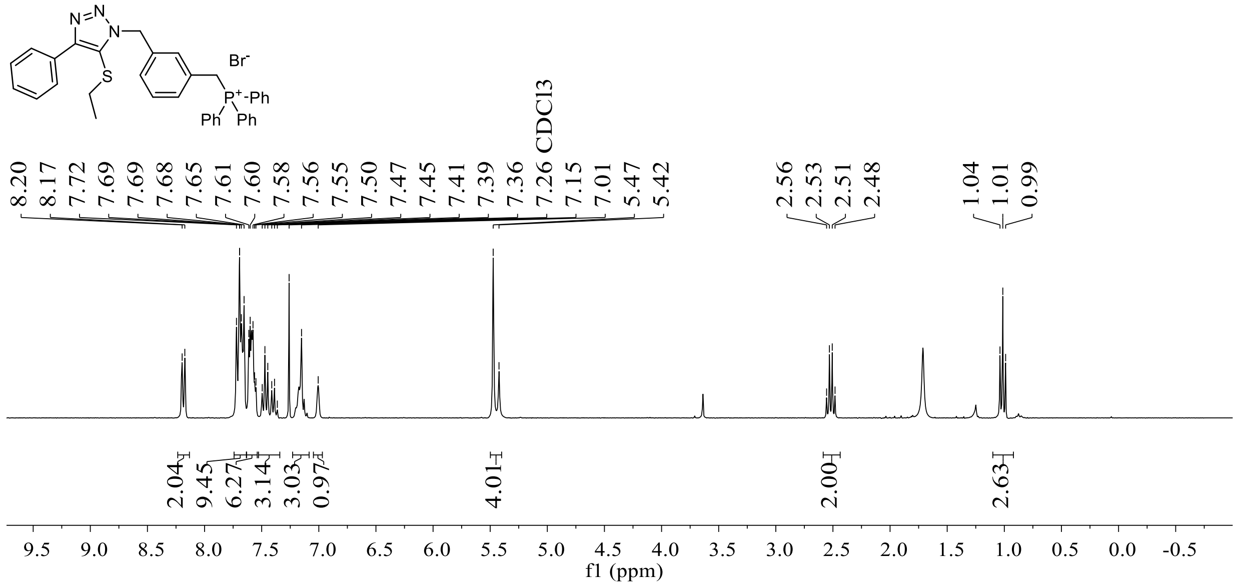

^{13}C ^{13}C-DEPT-135 (CDCl3, 75 MHz)

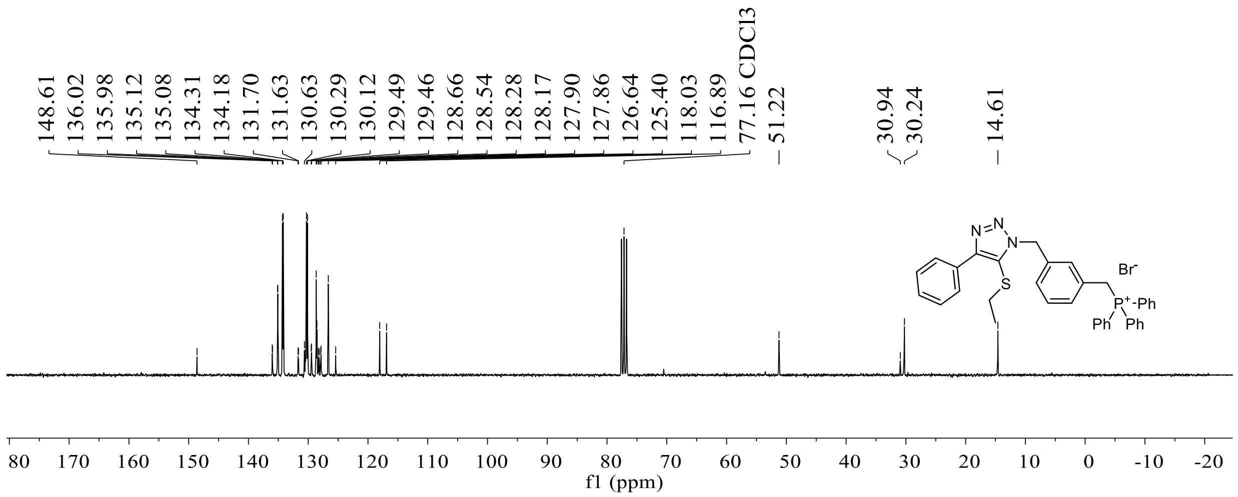

^{13}C-DEPT-135 (CDCl3, 75 MHz)

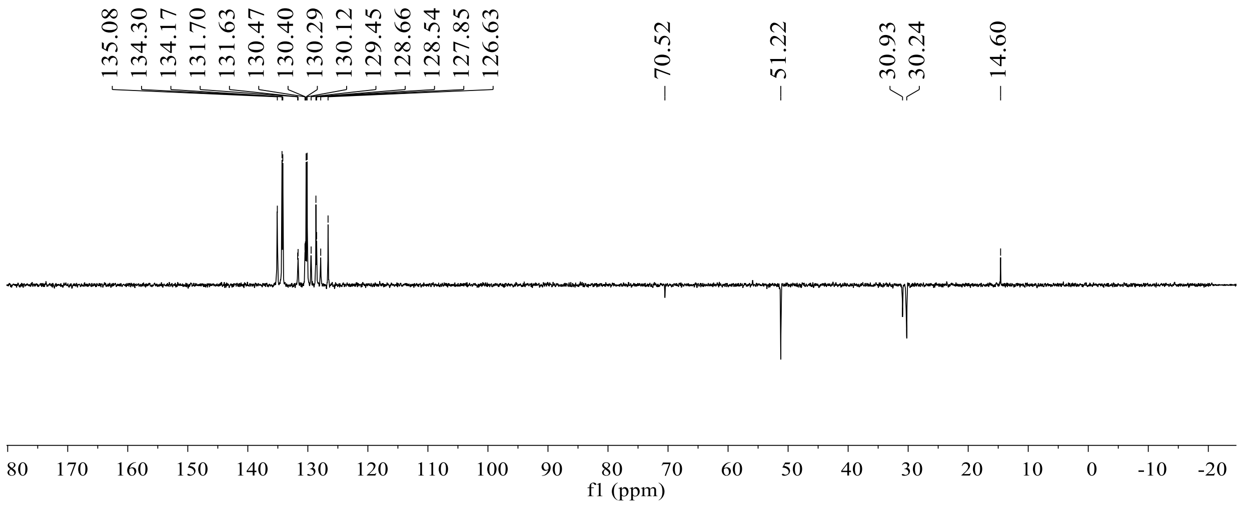

(4-(5-(*iso*Propylthio)-4-phenyl-1H-1,2,3-triazol-1-yl)butyl)triphenyl phosphonium bromide (3cd)

^{1}H ($CDCl_3$, 300 MHz)

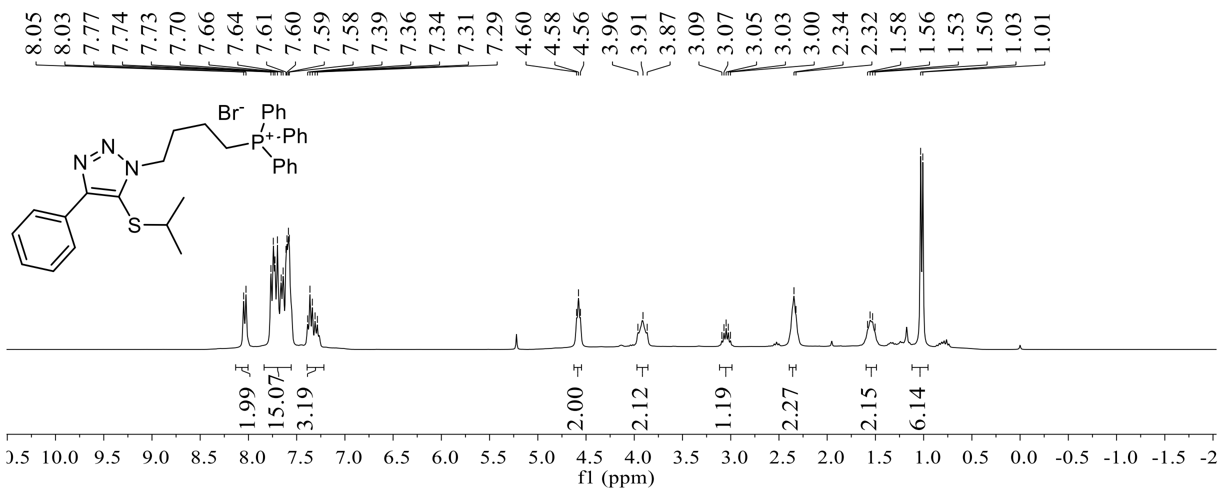

^{13}C ($CDCl_3$, 75 MHz)

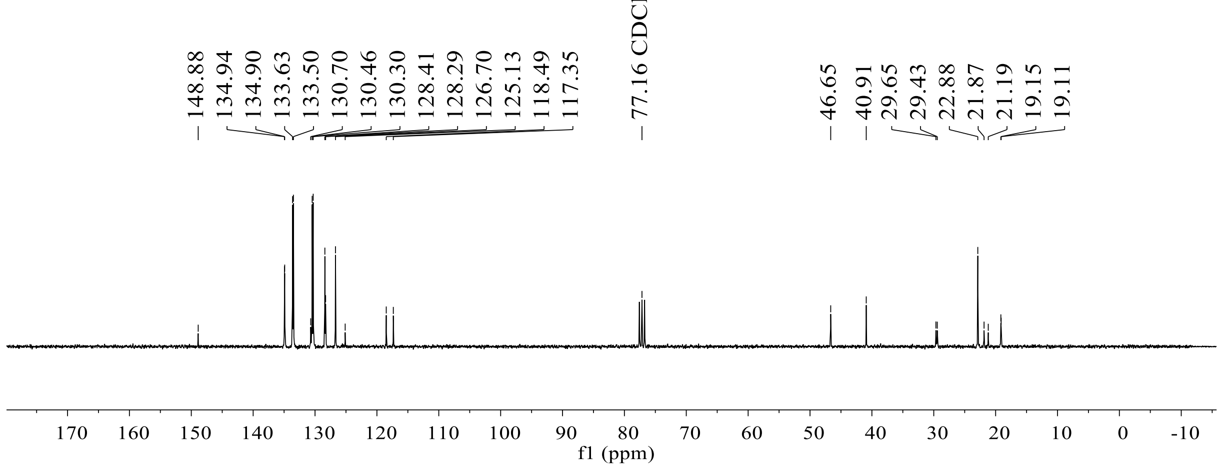

^{13}C-DEPT-135 ($CDCl_3$, 75 MHz)

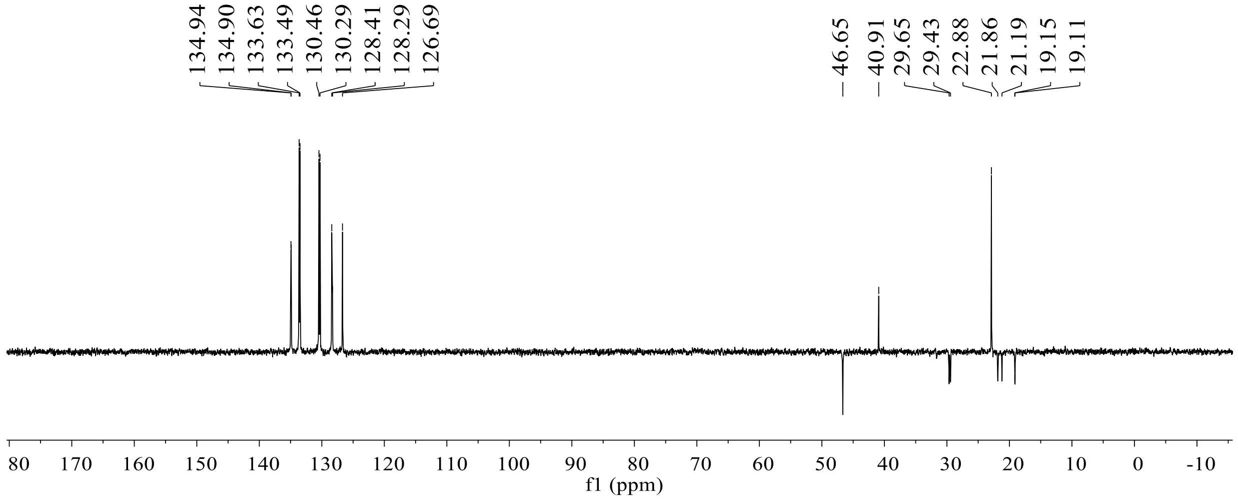
134.94
134.90
133.63
133.49
130.46
130.29
128.41
128.29
126.69
46.65
40.91
29.65
29.43
22.88
21.86
21.19
19.15
19.11
80 170 160 150 140 130 120 110 100 90 80 70 60 50 40 30 20 10 0 -10
f1 (ppm)

1-(4-Methylbenzyl)-5-(phenylthio)-4-(trimethylsilyl)-1H-1,2,3-triazole (3bb)

^{1}H ($CDCl_3$, 300 MHz)

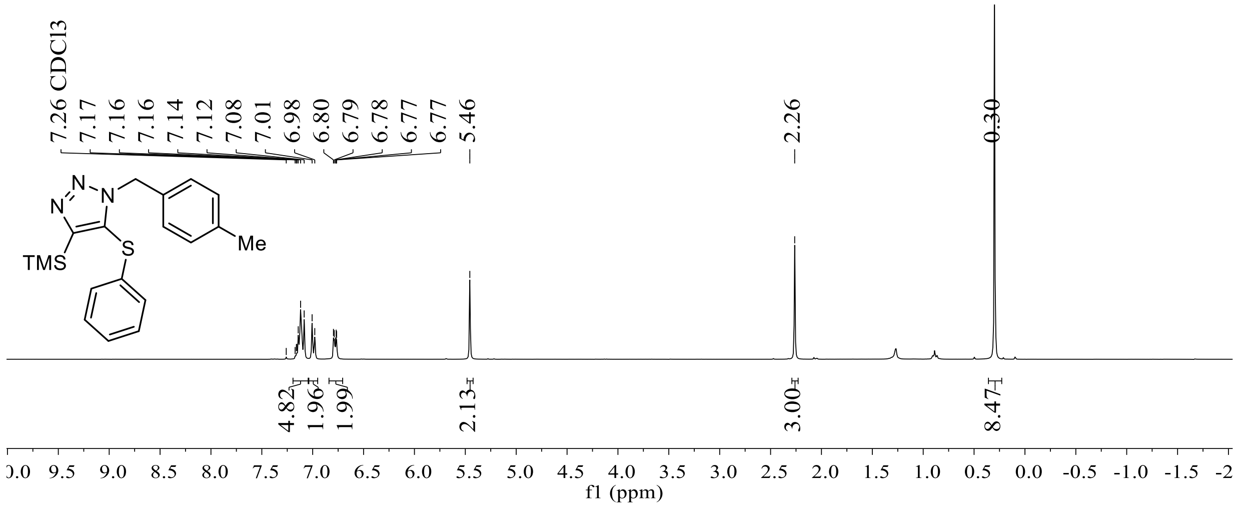

^{13}C ($CDCl_3$, 75 MHz)

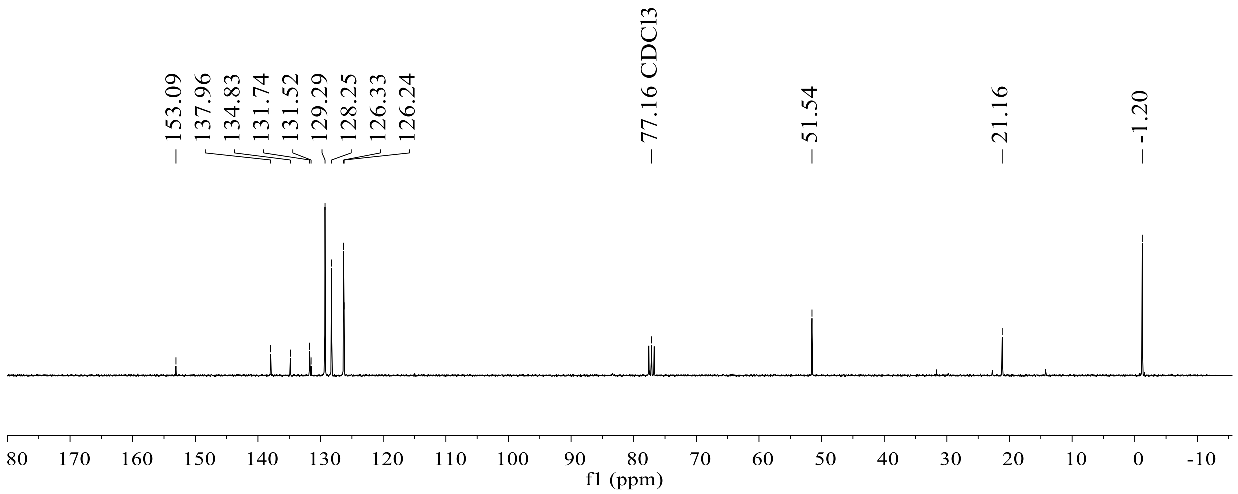

^{13}C-DEPT-135 ($CDCl_3$, 75 MHz)

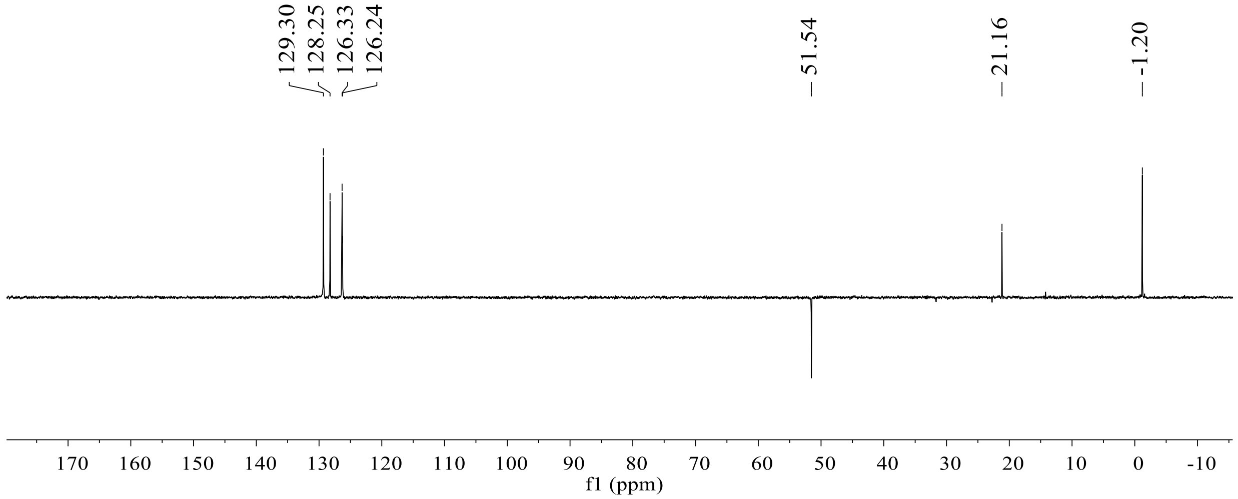

(4-(5-(Benzylthio)-4-(3-phenylpropyl)-1H-1,2,3-triazol-1-yl)butyl)triphenyl phosphonium bromide (3cc)

^{1}H (CDCl$_3$, 75 MHz)

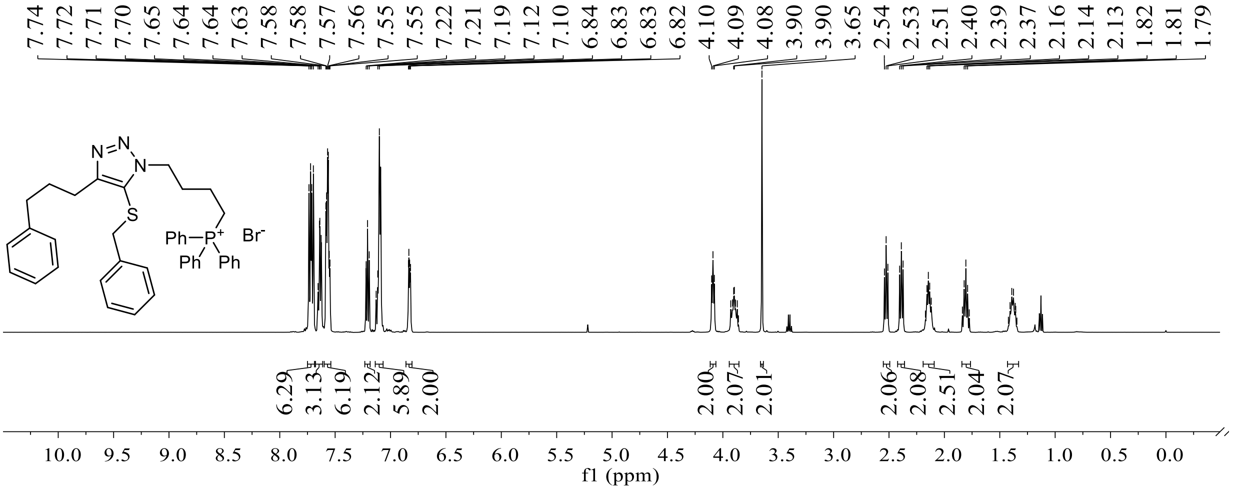

^{13}C (CDCl$_3$, 75 MHz)

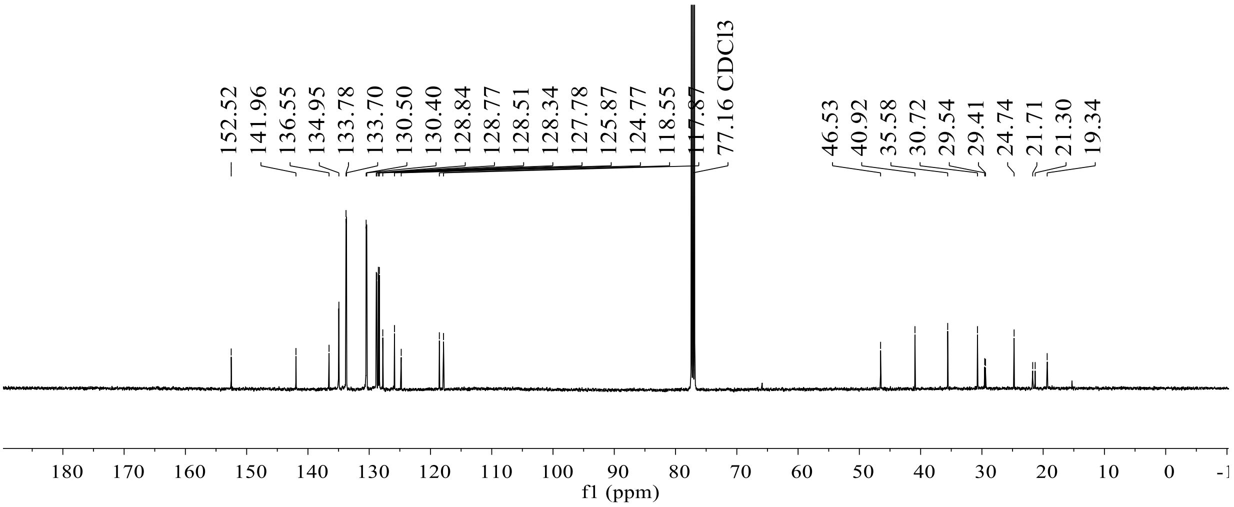

^{13}C-DEPT-135 (CDCl$_3$, 75 MHz)

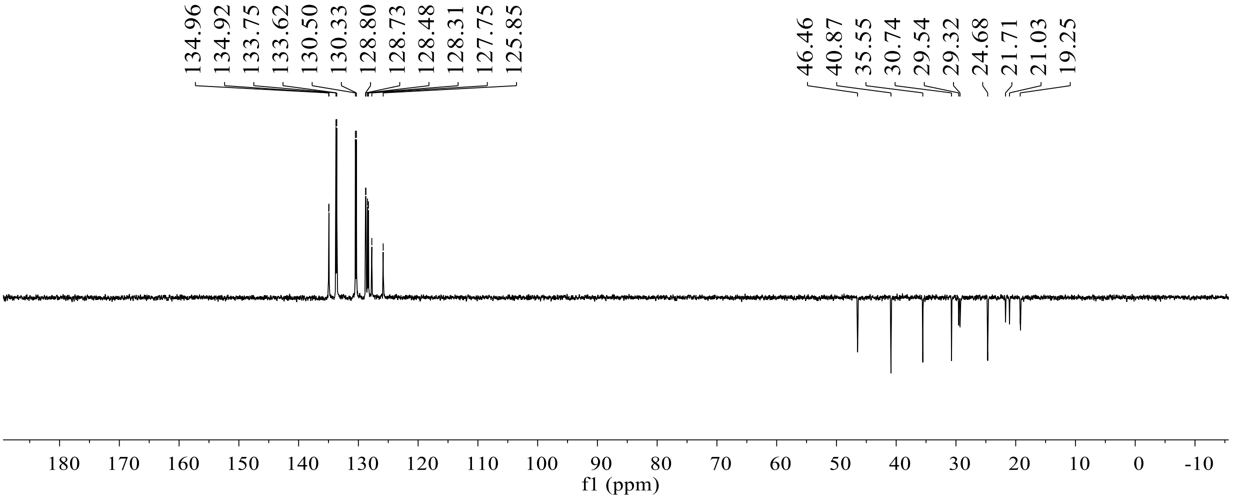

(S)-2-((((9H-Fluoren-9-yl)methoxy)carbonyl) amino)-6-(5-(ethylthio)-4-phenyl-1H-1,2,3-triazol-1-yl)hexanoic acid (3ea)

^{1}H (CDCl3, 500 MHz)

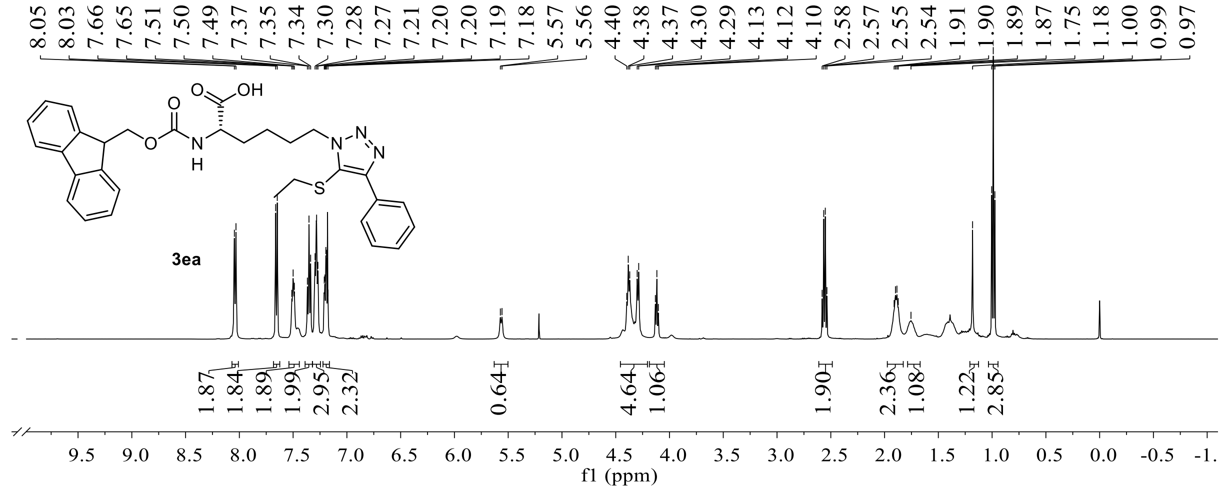

^{13}C-DEPT-135 (CDCl3, 126 MHz)

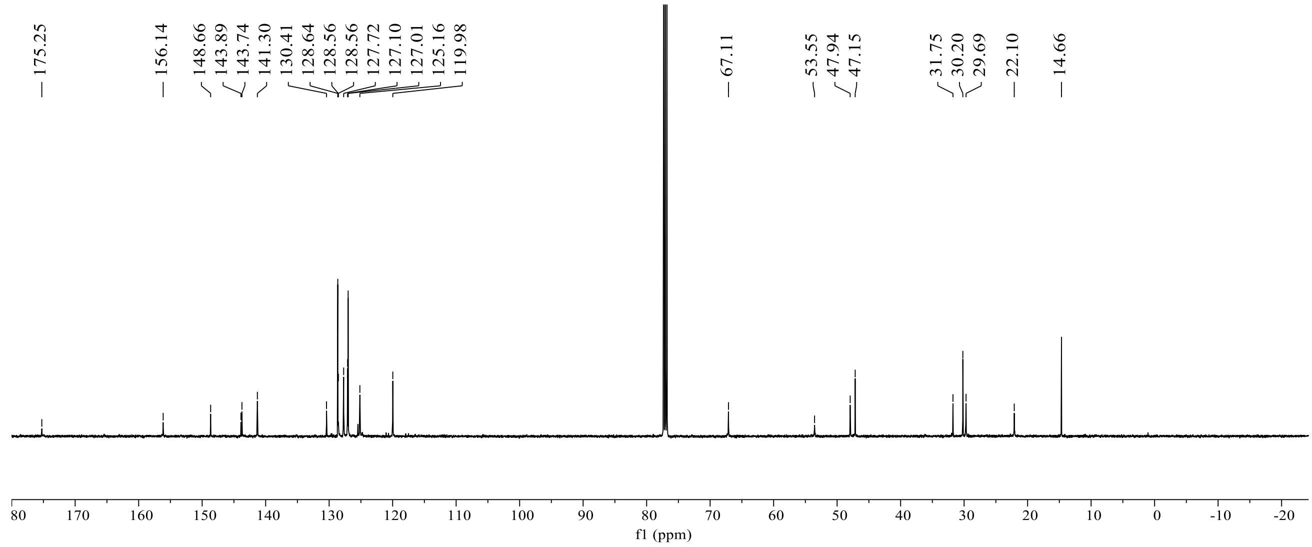

^{13}C-DEPT-135 (CDCl3, 126 MHz)

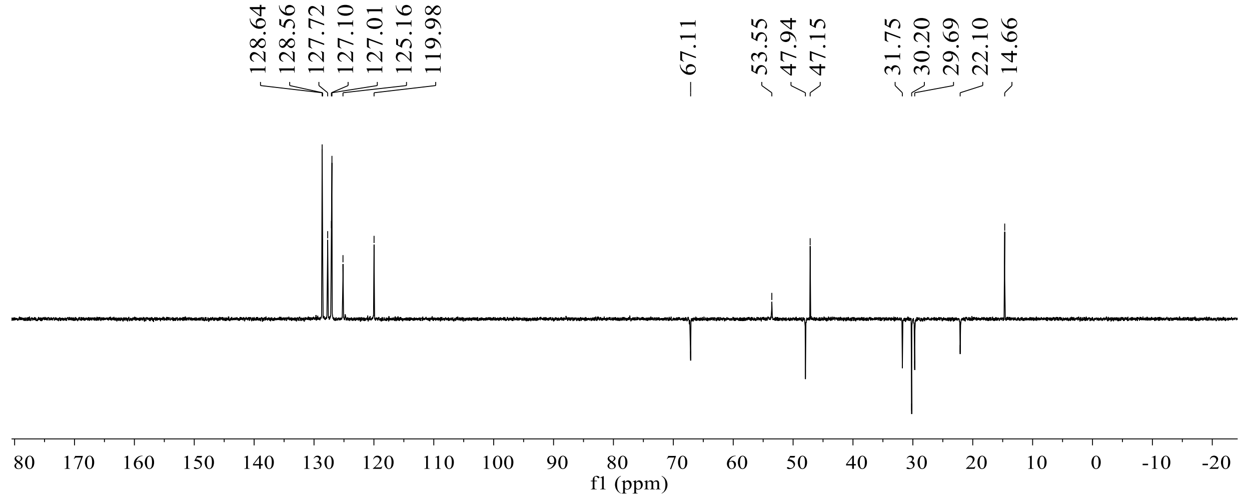

4-((5-(Ethylthio)-4-phenyl-1H-1,2,3-triazol-1-yl)methyl)-N,N-dimethylaniline (3fa)

^{1}H ($CDCl_3$, 300 MHz)

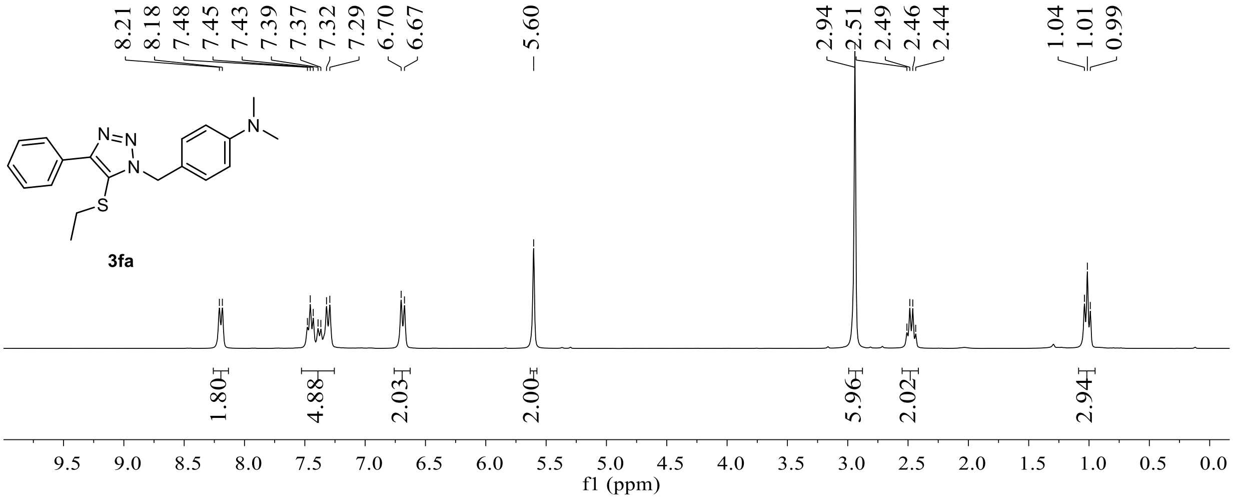

^{13}C ($CDCl_3$, 75MHz)

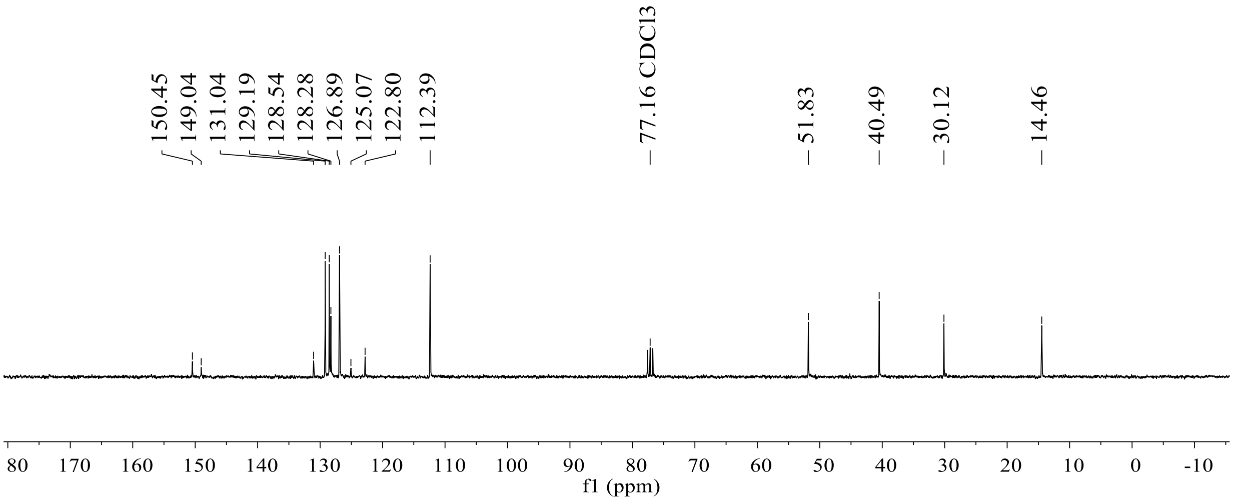

^{13}C-DEPT-135 ($CDCl_3$, 75MHz)

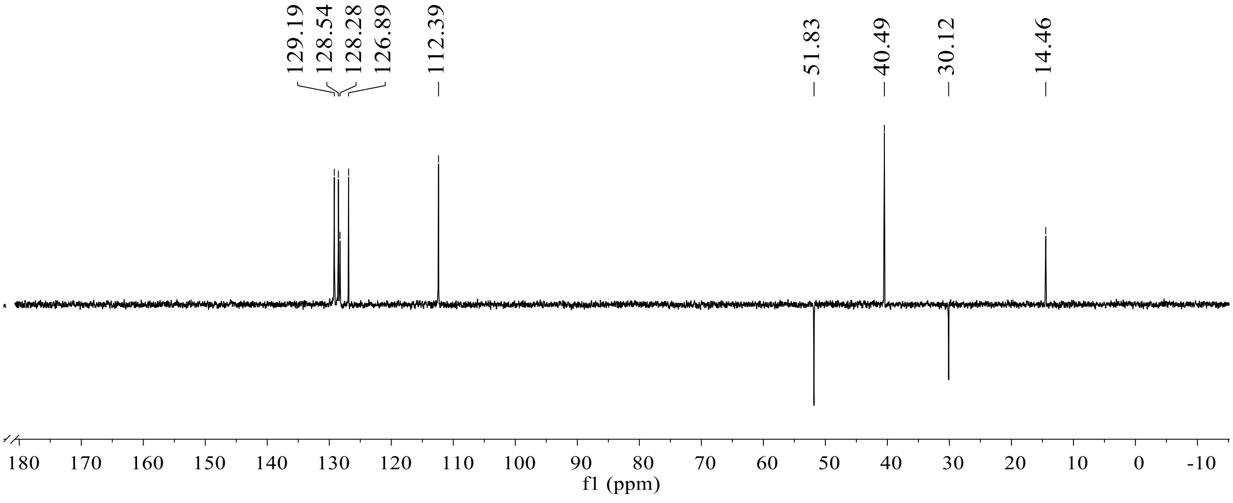

5-(1-(4-Methylbenzyl)-1H-1,2,3-triazol-4-yl)pentan-1-ol (3bh)

^{1}H CDCl₃, 300 MHz)

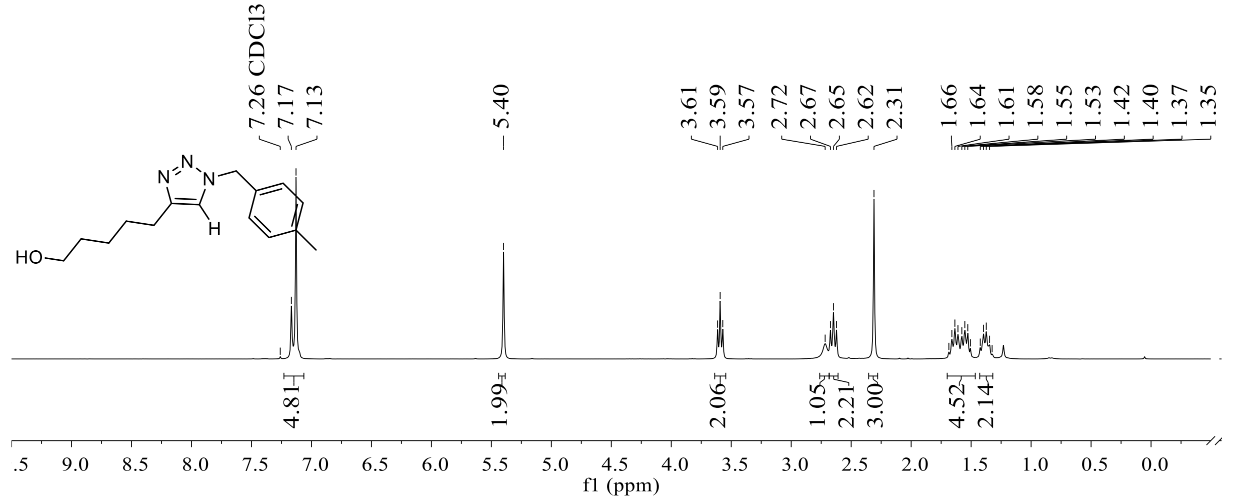

^{13}C (CDCl₃, 75MHz)

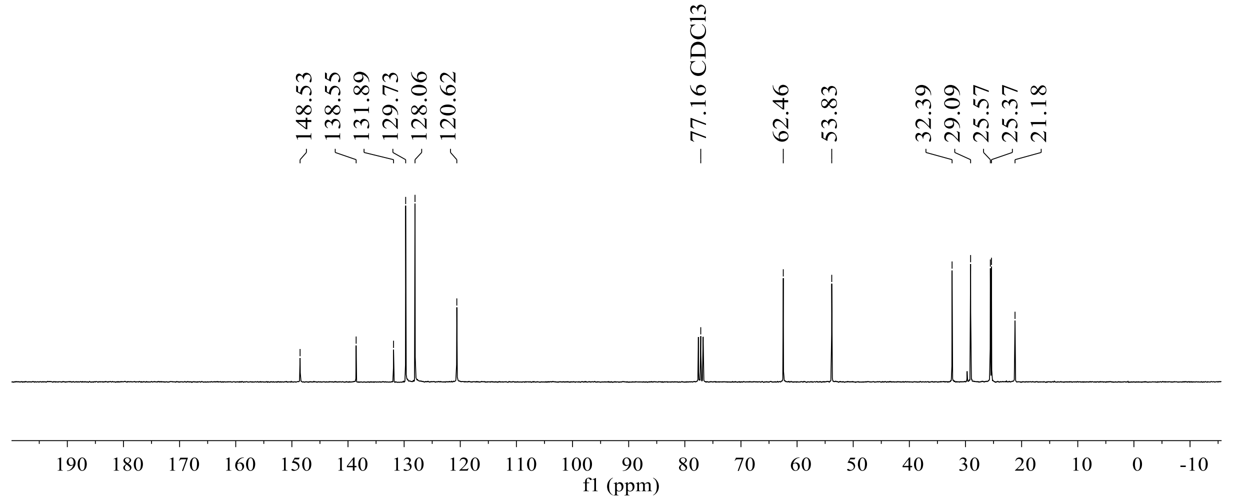

^{13}C-DEPT-135 (CDCl₃, 75MHz)

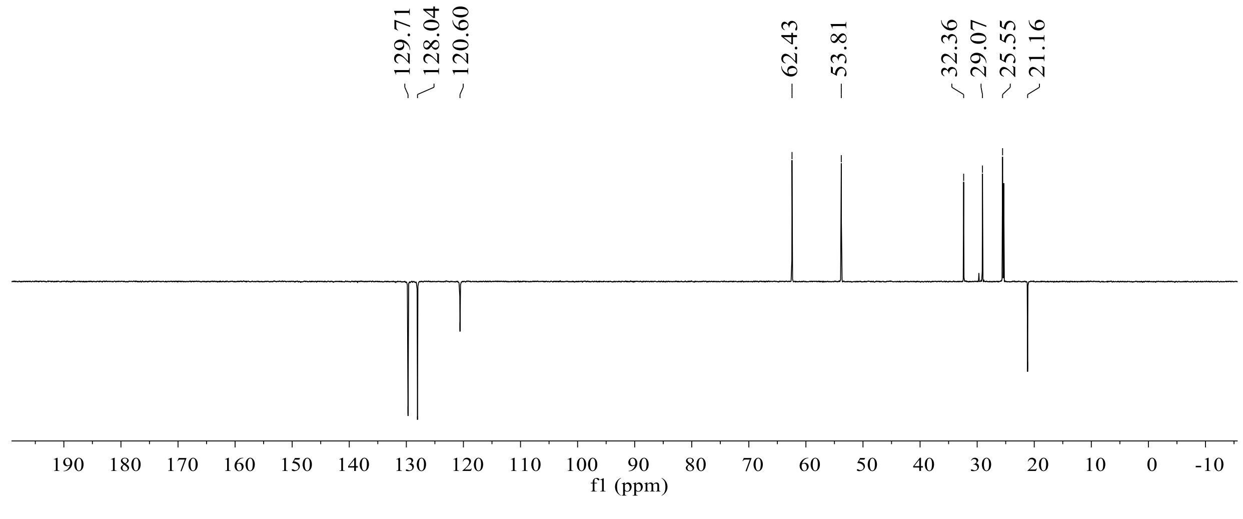

Supplement: Supplementary file 1 — Supplementary [file ANIE-60-16059-s001.pdf]
